# Fully automated registration of vibrational microspectroscopic images in histologically stained tissue sections

**DOI:** 10.1186/s12859-015-0804-9

**Published:** 2015-11-25

**Authors:** Chen Yang, Daniel Niedieker, Frederik Großerüschkamp, Melanie Horn, Andrea Tannapfel, Angela Kallenbach-Thieltges, Klaus Gerwert, Axel Mosig

**Affiliations:** 1 Department of Biophysics, CAS-MPG Partner Institute and Key Laboratory for Computational Biology, 320 Yueyang Road, Shanghai, 200031 China; 2 Department of Biophysics, Ruhr-University Bochum, Universitätsstraße 150, Bochum, 44780 Germany; 3 Institute of Pathology, Ruhr-University Bochum, Bochum, Germany, Bürkle-de-la-Camp-Platz 1, Bochum, 44789 Germany

**Keywords:** Multimodal image registration, fourier transform infrared microscopy, Spectral histopathology

## Abstract

**Background:**

In recent years, hyperspectral microscopy techniques such as infrared or Raman microscopy have been applied successfully for diagnostic purposes. In many of the corresponding studies, it is common practice to measure one and the same sample under different types of microscopes. Any joint analysis of the two image modalities requires to overlay the images, so that identical positions in the sample are located at the same coordinate in both images. This step, commonly referred to as image registration, has typically been performed manually in the lack of established automated computational registration tools.

**Results:**

We propose a corresponding registration algorithm that addresses this registration problem, and demonstrate the robustness of our approach in different constellations of microscopes. First, we deal with subregion registration of Fourier Transform Infrared (FTIR) microscopic images in whole-slide histopathological staining images. Second, we register FTIR imaged cores of tissue microarrays in their histopathologically stained counterparts, and finally perform registration of Coherent anti-Stokes Raman spectroscopic (CARS) images within histopathological staining images.

**Conclusions:**

Our validation involves a large variety of samples obtained from colon, bladder, and lung tissue on three different types of microscopes, and demonstrates that our proposed method works fully automated and highly robust in different constellations of microscopes involving diverse types of tissue samples.

**Electronic supplementary material:**

The online version of this article (doi:10.1186/s12859-015-0804-9) contains supplementary material, which is available to authorized users.

## Background

**Fig. 1:**
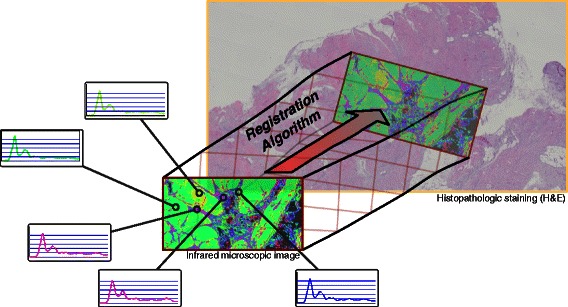
Problem overview. The FTIR image is a multi-spectral image where each pixel is represented by an infrared absorption spectrum (Five sample spectra shown). Typically, the FTIR microscopic image captures a small subregion of the complete H&E stained tissue section, turning the registration problem into a template matching problem

Comparing different microscopic images of one and the same sample obtained from different microscopic platforms is a major problem in many microscopic studies [1]. In spectral histopathology [2, 3], for example, it is common practice to first perform label-free microscopy using Fourier Transform Infrared (FTIR) microscopy of disease related tissue samples, and then apply classical Hematoxylin and Eosin (H&E) staining for a ground-truth annotation of the tissue sample by a pathologist. In different scenarios, a sample is first measured by Raman microscopy, and subsequently immunohistochemically or histopathologically labeled and imaged under a conventional light microscope [4, 5]. In yet another setting, the sample may be simultaneously studied using Raman and MALDI imaging [6]. In such procedures, a key step is to align the two images from both microscopic modalities as illustrated in Fig. 1, where the two images are overlaid in a way that each spot in the sample is located at the same pixel coordinate in both microscopic images. This may involve different types of geometric transformations such as translation, rotation, scaling, or in some cases even non-linear distortions. Finding the correct geometric transformation for an overlay is commonly known as *image registration* in the image processing literature.

More specifically, it is often the case that one microscope allows to capture a large area, e.g. encompassing a complete sample, whereas the second microscopy platform may only capture a smaller specific region of interest. Following common image processing terminology, this setting leads to a *template matching problem*.

Clearly, a reliable, efficient, and fully automated algorithmic approach suitable for template-matching registration tasks in different constellations of microscopes will mean a great simplification of data analysis in cross-microscopy studies and is thus the core motivation of this contribution.

For certain cross-modality microscopic image registration tasks, specific approaches have been proposed previously. Schaaff et al. [7] proposed a semi-automated approach for registering secondary ion mass spectrometry (SIMS) images against optical images. Their registration approach has been used recently by Bocklitz et al. [6] to register Raman microscopic images against mass spectrometric MALDI-TOF images. In another recent study [8], the authors have utilized an automated image registration approach for registering FTIR images of tissue microarray (TMA) cores against H&E images. However, these previously proposed methods are either not fully automated, or they are targeted towards specific sample conditions such as the registration of round-shaped TMA cores and thus have not been validated on a broad basis of different types of samples and microscopy platforms.

Image registration becomes more challenging when dealing with conventional histopathological thin-section slides, which cover relatively large areas of few square centimeters. The complete slide can be captured routinely and with high resolution as an H&E image using microscopes or specialized slide scanners. When dealing with vibrational microscopy, it is often the case that the spectral image only captures a small region of interest (ROI) of the sample, turning the registration problem into a template matching problem. Note that template matching in general is much more challenging than global registration. On the one hand, the search space of transformations to be explored is larger than for global registration, thus making template matching computationally more expensive. On the other hand, the target function needs to be much more specific towards the correct registration position, as a larger search space will also leave more space for false registration positions.

Our contribution deals with the problem of registering a vibrational microspectroscopic image against a histopathologically H&E stained image in a template matching setting, as illustrated in Fig. 1. The vibrational microspectroscopic image is a hyperspectral image where each pixel is represented by either an infrared absorption spectrum acquired by FTIR spectroscopy or an emission spectrum acquired by a *Coherent Anti-Stokes Raman Spectroscopy* (CARS) microscope. Each pixel spectrum is represented as a high dimensional vector of optical absorptions at several hundred wavenumbers, which overall represent the biochemical status of the sample at the corresponding pixel location. The conventional histopathological image is obtained a light microscope after H&E staining of the sample, yielding an image in RGB color space.

In some specific cases, it has been demonstrated that a *foreground-background* separation can be utilized for registration. In both the H&E image and the spectral image, it is relatively easy to identify areas not covered by sample, which will be regarded as background, while all other positions in the image will be regarded as foreground. This leads to binary versions of both the H&E and the spectral image, which can be used for registration. In some cases such as individual TMA cores, foreground-background separation is morphologically informative (e.g. due to the structure of cracks or holes in the sample), so that it will be a sufficient basis for registration [8]. Under these circumstances, registration can be achieved with relatively simple standard registration algorithms. In many cases, in particular when dealing with subregions of whole-slide images, foreground-background separation may not be sufficient, so that registration will require more advanced computational techniques on images that carry more information than merely a foreground-background segmentation. In fact, subimage registration in whole-slide-images constitutes the focus of our contribution.

### Introduction

**Vibrational Microspectroscopy and Spectral Histopathology** Several microscopy techniques have been established in recent years to capture spectra of molecular vibrations at high spatial resolution. Most notably, *Fourier Transform Infrared* (FTIR) microscopy allows to characterize optical absorption of biological samples through absorption spectra covering the infrared part of the optical spectrum. Each pixel thus is represented by an infrared spectrum, which is characteristic for the biochemical status of the sample at the corresponding pixel location at a spatial resolution of a few microns. It has been shown in many types of tissue including colon [3, 4], lung [2, 9], cervix [10] or skin [11] that the infrared spectra obtained by FTIR microscopy are highly characteristic to resolve tissue structure and identify areas affected by diseases such as cancer. The application of vibrational microspectroscopy for diagnostic purposes has also been termed *spectral histopathology* (SHP). SHP involves computational learning of spectral properties, which are usually obtained by overlaying histopathologically annotated H&E stained images with the corresponding FTIR microscopic image. This clearly involves an image registration step, which to date has in general been conducted manually.

Beyond FTIR microscopy, other types of vibrational microspectroscopy have been utilized for diagnostic and other purposes. Most notably, Raman and CARS microscopes allow to characterize biological samples based on optical emission spectra at a spatial resolution of several hundred nanometers. While Raman microscopy captures spectra along a broad spectral scale in a relatively time consuming process, CARS microscopy allows to capture selected parts of the spectrum, e.g. at few dozens of wavenumbers, at high speed, making it suitable for characterizing sufficiently large regions within tissue samples.

**Terminology** It is common practice in histopathology to capture the complete sample within one large H&E image. Conversely, the spectral image is often only captured within a relatively small subregion of the H&E image, thus turning the registration problem into a multimodal version of what is typically referred to as a *template matching problem*. We adapt the corresponding terminology and will treat the spectral image as the *template*, which will be considered as the *fixed* image, while the stained image will be the *moving image* undergoing transformation. The aim is to find a geometric *transformation* that maps the coordinates from the spectral image to the stained image. Since scale bars are typically available for both images, the H&E stained image can be rescaled to match the resolution of the FTIR image, so that the admissible class of transformations are *rigid motions* composed of a translation in *x* and *y* direction and a rotation. Sometimes, a small degree of scaling is required to adjust minor uncertainty about the scale bar information which may get lost during image acquisition for technical or other reasons.

**Area-based vs. feature based registration** Existing approaches to image registration can be classified into two categories according to their essential strategies [12], namely *area-based* (or intensity-based) approaches on the one hand and *feature-based* (or *landmark-based*) approaches on the other hand. The area-based strategy assigns a fitness to each possible transformation [13, 14]. Measuring the fitness of one specific transformation is accomplished by the means of a *scoring function* or *metric*, which in most cases takes into account all pairs of overlapping pixels between the two images. The feature-based strategy first extracts the salient structures, i.e., the features [15, 16], of an image and estimates the transformation by matching feature pairs [17, 18]. For registration across different modalities, however, it is generally challenging to obtain identical feature points across both types of images using state-of-the-art approaches to feature point identification, as indicated in Additional file 1: Figure S2. Area-based matching methods are usually considered more accurate since they use more available image information [19], although they can be slow in practice even when dealing with a simple transformation type. Computational efficiency issues become even more pronounced in our setting focused on template matching.

**Components of area-based registration** An area-based registration procedure usually contains four major components [13]: 1) A *metric* which is a similarity measure of how well two images overlap under a particular transformation. 2) The *geometric transformation* represents the spatial mapping of the coordinates of points from one image to the other. 3) The *interpolator* is used to evaluate transformed image values at non-grid positions. 4) An *optimizer* searches the optimization space defined by the parameters of the transformation to find the best transformation. In the sense of an optimization problem, the metric and the interpolator define the objective function that needs to be optimized; the transformation class defines the domain of definition; and the optimizer searches this domain for an optimal value of the objective function.

### Challenges in cross-modality image registration

Challenges in registering vibrational microspectroscopic images against their H&E stained counterparts arise from the inherently different traits of these two types of microscopy. Spectra obtained from FTIR, Raman or CAR microscopes, on the one hand, are highly specific for different tissue components and thus allow to resolve tissue structure reliably. On the other hand, vibrational spectra, in particular FTIR spectra, exhibit very low variability across different samples, individuals, or even FTIR microscopes of different manufacturers [3], making vibrational microspectroscopy an ideal tool for resolving tissue structure. However, vibrational microspectroscopic images are inherently high-dimensional, as each pixel is represented by a vector containing extinctions or emissions at usually several hundred wavenumbers. H&E images, on the contrary, are usually available as RGB images and thus low-dimensional in nature. Processing H&E images computationally is yet complicated by inhomogeneities resulting from tissue preparation and staining, leading to a very variable range of contrasts and other parameters covered within collections of H&E stained images. The two modalities also display tissue structure on different scales of resolution. While the pixel resolution of FTIR microscopic images is around 5 *μ*m, H&E stains can be measured with a resolution close to the diffraction limit of few hundred nm.

The major difficulty of the registration task is that the two modality signals are different in dimensionality and carry very different information. The colors in the H&E stained image, on the one hand, display the response of the tissue components to staining by hematoxylin, which tends to stain nuclei, and eosin, which stains cytoplasm. The absorptions captured in an FTIR spectrum as well as the emissions in a Raman or CARS spectrum, on the other hand, represent an integral snapshot of the biochemical components at a given pixel location. Clearly, the two signals represent information of inherently different origin, so that typical measures of correlation fail to reflect similarity relevant for registration appropriately. (Additional file 1: Figure S1).

Thus, the relationship between H&E staining colors and the spectra is generally enigmatic in the sense that diverged spectra do not necessarily mean bigger color differences in the image. In theory, two locations with identical spectrum can possibly have quite different staining patterns and vice versa even in a hypothetical scenario without noise or artifacts. While it has been demonstrated recently [20] that using an infrared image, the sample can “virtually stained” by inferring an image visually similar to a corresponding H&E image. However, similarity is in general not sufficient for registration (see Additional file 2: Figure S6), beside requiring tissue-specific training of regression classifiers.

### Overview of contributions

**Utilizing Presegmentations** A major novelty of our approach is to utilize discrete presegmentations of both the spectral and the H&E image obtained from clustering the pixel spectra or intensities. The only existing approaches reduce information in both modalities to binary foreground-background segmentation, which disregards most information contained in the images and thus may severely limit the robustness of registration. On the other hand, it is well-established that clustering unveils tissue structure in FTIR, Raman and CARS images relatively well in general, and is in fact commonly used for spectral image segmentation. While supervised or interactive [21, 22] approaches are known to achieve segmentations that display biologically relevant structures more accurately, such approaches require either prior knowledge or manual interactions, contradicting our goal of achieving a fully unsupervised registration. It is also known that hierarchical clustering represents tissue structure better than non-hierarchical approaches [21], but at the cost of significantly higher demands in running time and memory.

**Sparse Search Optimizer** Another challenge is constituted by the size of the images involved. Images of a complete H&E stained microscopy slide will comprise tens of millions of pixels even after a coarse reduction of resolution. Vibrational microspectroscopic images also tend to involve large amounts of data, as they involve hundreds or thousands of spectral components. Due to the large amounts of data involved, computational efficiency is a crucial issue, in particular for the optimization approaches that are part of corresponding registration approaches. To address this issue, we propose a *sparse search* optimization strategy.

**Self-registration based initial radius estimation** The sparse search optimizer requires initial coarse-level grid points in transformation space. We propose a novel approach to estimate an optimal distance between grid points based on self-registration of the template image.

## Methods

### Biological sample materials and preprocessing

**FTIR image acquisition and sample preparation** In total nine histological sections from colon, lung and bladder tissue were prepared following procedures established in previous work [3] by first taking FTIR microscopic images on an *Agilent Cary* microscope, and subsequently performing H&E staining using an *Olympus BX41* microscope. FTIR image spectra were preprocessed using the resonance Mie scattering correction procedure by Bassan et al. [23]. Subsequently, following [3], the wavenumber scale was adjusted.

For identifying background spectra, two different approaches were employed. The first approach utilized the procedure introduced in [3], where confounded spectra are identified by different layers of filtering and classification at the level of resonance Mie corrected spectra. All pixel spectra rejected on the grounds of these steps were regarded as *background* pixels, the remaining pixels as foreground. For some of the samples, background separation was conducted on the basis of raw uncorrected spectra. In this case, each pixel was assigned the integral absorption throughout the complete infrared spectrum. In the resulting intensity image, a cutoff intensity was automatically determined, so that all pixels exceeding this cutoff were considered foreground and the remaining pixels considered background. The latter approach in general identifies areas not covered by sample more accurately, but requires spectral information on two different levels, namely both the raw spectra and the resonance Mie scattering corrected versions for further processing. Note that the latter approach cannot be conducted on spectra after the resonance Mie scattering correction, as the resulting spectra appear implicitly normalized, so that background spectra exhibit a high degree of integral absorption.

For preprocessing the H&E images, a first step reduced the resolution of the H&E image to the resolution of the FTIR image using the *imresize* function of the *Matlab* image processing toolbox. The second step was to adjust color cast which is commonly observed in H&E staining images due to the variability of the H&E staining and image acquisition procedure. In order to eliminate color cast, we first estimate a color vector representing the background color which in theory should be pure white since no light is absorbed in the absence of the tissue by a simple thresholding (Additional file 1: Figure S3). Then we subtract this color vector in the Optical Density space [24], so that the color of the stained images are corrected. Finally, we set all colors close to the color vector to pure white which indicates the background.

**CARS image acquisition and sample preparation** CARS-microscopy was performed on a commercially available setup (TCS SP5 II CARS; Leica Microsystems, Heidelberg, Germany) based on a picosecond-pulsed laser system which generated two synchronized, colinearly aligned beams and using an inverted confocal microscope as described before [5]. To focus the laser beam, a HCX IRAPO L (25X/0.95 W; Leica Microsystems) objective was used. The typical pixel dwell time was 32 *μ*s per pixel, with 34 s per image, 1024 ×1024 pixels, a 300 *μ*m × 300 *μ*m area and a pixel resolution of 300 nm. The CARS intensity in the C-H stretching region from 2700 to 3000 cm ^−1^ was measured with a spectral resolution of 5 cm ^−1^ to obtain spectra. For details, we refer to the equivalent CARS setup described in [5].

**Fig. 2:**
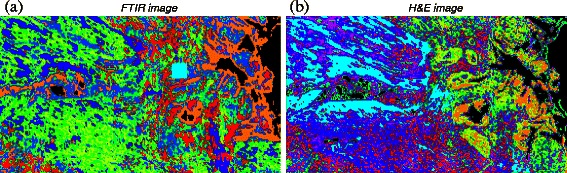
Index color images acquired by *k*-means clustering. **a** Index image of the spectral image using *k*=8; (**b**) index image part of the corresponding area in the stained image using *k*=10. Black color corresponds to background; the cyan square in the spectral image is caused by a measurement artifact. Obviously, no one-to-one correspondence between the color indices of the two images exists. The mutual information of these two images is 0.5145, while the entropies of two images are 3.1196 and 2.9664, respectively. Note the background segmentation is not perfect, e.g. in the spectral image, the orange segment should be identified as background

**Segmentation of images** We applied *k*-means clustering with the *k*-means++ seeding [25] scheme to obtain presegmentations of the FTIR spectral image. A corresponding presegmentation of the H&E image was obtained by clustering the three-dimensional RGB vectors of all foreground pixels. Examples of the resulting index color images of two corresponding FTIR and H&E regions are displayed in Fig. 2. Whenever not specified otherwise, we presegmented whole slide images into a default number of *k*=10 clusters, and smaller ROIs in the FTIR images into *k*=8 clusters.

### Registration strategy

We describe our registration approach following the four main components of an area-based registration procedure.

**Transformation and interpolation** In general, we allow registration under the group of *similarity transformations*, i.e., translation, rotation, and scaling to transform the moving image. Whenever applicable, this was further restricted. If precise scale bar information was available on both images, transformations were restricted to *rigid motions* (rotation and translation); in case rough but imprecise scaling information was available, the scaling factor was limited to a suitable interval close to a factor of 1. Sometimes, either a precise prior knowledge or a rough estimate of the rotation was available. In these cases, either pure translation or translation plus a restricted interval of rotations (e.g. ±30 degrees) was allowed. Some existing registration approaches are too slow to achieve registration under rigid motions in practice. Thus, whenever necessary, transformations were restricted to translations for efficiency reasons.

When dealing with geometric transformations on pixel images, an interpolation of the transformed moving image is conducted, as pixels in transformed coordinates overlap several neighbouring pixels of the original image. As our registration approach relies on index color images obtained from presegmentations, *nearest-neighbour interpolation* (i.e. taking the index color from the closest transformed pixel in the transformed image pixel coordinates) is the straightforward choice.

**Metric** We propose *restricted mutual information* as a registration metric closely related to the weighted version of mutual information proposed earlier [26]. This measurement serves as a universal metric for measuring nonlinear correspondence with the prior knowledge that certain index pairs, in our case indices representing background in each modality, should or should not match each other.

Conventional mutual information (MI) is an obvious choice in order to measure the nonlinear correlation between two index color images (where the index value itself does not contain information). Yet, when using mutual information in a template matching registration, we encounter the problem of *background attraction*, where MI tends to score highest in large segments representing image background not covered by sample (see Additional file 2: Figure S1). In order to utilize both the known foreground-background matching and the nonlinear correlation between signal segments, we introduce *restricted mutual information* that can incorporate certain prior knowledge into mutual information. Restricted mutual information is based on interpreting mutual information as a matching score that uses the observed joint probability distribution as scoring scheme (see Additional file 2: Section A.1). Under this interpretation, we modify mutual information to incorporate prior knowledge about matching indices between the two images by adjusting the joint probability estimated from data. In our case, this adjustment is based on the assumption that indices *X*=0 and *Y*=0 represent background pixels in both the spectral and the H&E image.

Now, restricted mutual information adjusts the probabilities *p*(*i*,*j*) according to background information through an adjustment factor *α* through defining

$$\begin{array}{@{}rcl@{}} P_{\alpha}(X | Y = 0) &= &(1 - \alpha) \, P(X | Y = 0), \\ P_{\alpha}(Y | X = 0) &= &(1 - \alpha) \, P(Y | X = 0), \\ P_{\alpha}(X = 0, Y = 0) &= &(1 - \alpha) \, P(X = 0, Y = 0) \quad\text{and}\\ && + \ \alpha \, P(X = 0 \cup Y = 0), \end{array} $$

where *P*(*X*=0∪*Y*=0)=*P*(*X*=0)+*P*(*Y*=0)−*P*(*X*=0,*Y*=0), and we conveniently define *p*_*α*_(*i*,*j*):=*P*_*α*_(*X*=*i*,*Y*=*j*).

Here, the adjustment factor *α* is treated as a parameter of the registration pipeline. We denote the modified joint probability as *P*_*α*_(*X*,*Y*) as our restricted scoring scheme that incorporates our prior knowledge. Then joint entropy indicates the quality of the matching and can be defined as

$$\begin{array}{@{}rcl@{}} H_{\alpha}(X,Y) & = &-\frac{\log\prod_{i=1}^{m}P_{\alpha}(X=A(i),Y=B(i))}{m}\\ & = & -\sum_{x\in\mathscr{X}}\sum_{y\in\mathscr{Y}} p(x,y) \log p_{\alpha}(x,y), \end{array} $$

so that we can define *restricted mutual information* as

$$ I_{\alpha}(X,Y) = H(X) + H(Y) - H_{\alpha}(X, Y). $$

For details regarding restricted mutual information, we refer to Additional file 2: Section A.1.

**Optimizer** A main challenge when dealing with subregion matching is that the space of potential transformations is very large, so that an exhaustive search that assesses the target function for essentially every possible transformation will be prohibitively slow. To address this issue, in particular in the light of large image sizes in histopathology applications, we developed a *sparse search optimizer* which in practice requires only a small part of the search space to be evaluated.

**Fig. 3:**
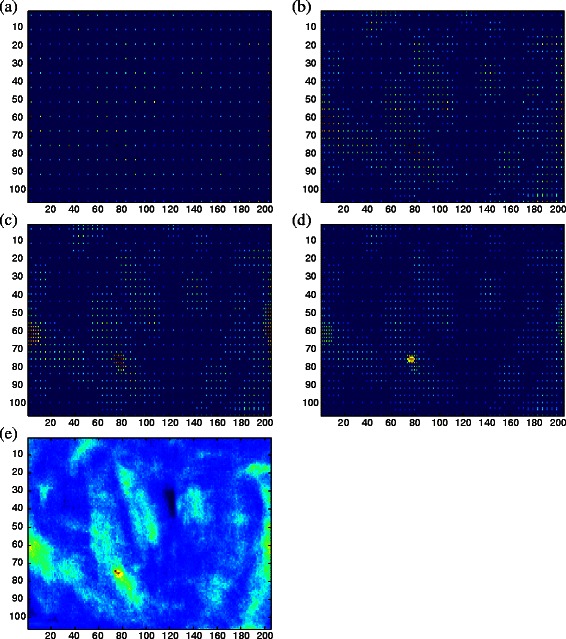
Sparse search under 2D translation. The optimizer (fully described in Additional file 2: Section A.2) restricts the search space to all translations indicated by non-dark-blue grid points. **a** Search space under initial radius *r*=*r*_0_=8 and threshold *ν*_thresh_=0.6 times the maximal intensity; (**b**) Search space after second iteration with *r*=4 and *ν*_thresh_=0.6·0.5; (**c**) third iteration with *r*=2 and *ν*_thresh_=0.6·0.5^2^; (**d**) final iteration with *r*=1, *ν*_thresh_=0.6·0.5^3^. Panel (**e**) shows the complete score map of all possible translations. The efficiency of the optimizer results from the fact that only few points of the complete score map need to be evaluated. While in this example, the grid radius *r* is treated identical for all parameters of the transformation, we generally compute a separate radius for each parameter using self-registration based initial radius estimation

The sparse search optimizer follows a straightforward approach that is illustrated in Fig. 3 and detailed in Additional file 2: Section A.2. The algorithm successively computes subsets of equidistant grids with radius *r* decreasing in each iteration. In the next level of detail, only those grid points are kept which have a high-scoring neighbour under target function *f* in the previous level.

A crucial step in the sparse search procedure is the choice of the initial distance *r*_0_ between grid points on the coarsest grid. If *r*_0_ is too large, the optimizer will fail to detect the optimal solution. If, on the other hand, *r*_0_ is too small, the efficiency advantage diminishes. In order to avoid introducing *r*_0_ as a parameter, we propose a novel and fully automated approach to determine *r*_0_ through *self-registration* of the moving image against itself. Performing this self-registration under one parameter of the transformation space yields a peak in the target function under the neutral transformation. As detailed in Additional file 2: Section A.2.2, the width of this peak can be utilized to estimate *r*_0_.

The sparse search procedure can be seen as a generalization of the image pyramid [27], which is a commonly employed multi-scale image representation strategy to reduce the running time of registration procedures. In practice, however, it is hard to use more than two or three levels of a pyramid before important structures of the image get lost [28]. The sparse search optimizer avoids this problem by decomposing the search space rather than the image itself. This allows a much higher degree of “sub-sampling” as no image information for the metric will be lost. With sparse search, we utilize image information on a lower level while searching the transformation space on a higher level.

## Results and discussion

We extensively evaluated our registration approach in mainly three scenarios. First, we assessed its performance in *subimage registration:* We registered FTIR images displaying subregions of histopathological slides, which were captured completely in the H&E image. Second, we performed *whole-spot- registration* of tissue microarrays. As a third scenario, we demonstrate the performance of our registration method in the registration of a Coherent Anti-Stokes Raman (CARS) microscopically imaged region of interest within an H&E image of the same sample. A fourth and less extensive assessment has been conducted for full-slide registration, where both an H&E and an FTIR image are given for a complete microscopic slide (samples S6–S9). As it turns out, in this scenario our proposed method works reliably, as well as conventional registration approaches implemented in standard toolboxes such as the Matlab *imregister* function or the ITK *Insight Segmentation and Registration Toolkit* [13] (data not shown).

In the scenarios described above, we evaluated four different registration approaches:

- *RMI/sparse*: We used restricted mutual information between clustering-based presegmentations as described above in combination with our newly proposed sparse search optimizer.
- *Binary RMI/sparse*: We used restricted mutual information in combination with binary foreground/background segmentations and the sparse search optimizer.
- *Binary MS/gradient*: We used a mean-squares metric on binary foreground-background segmentations in combination with a gradient optimizer, which essentially corresponds to the approach proposed in [8].
- *Binary MS/evo*: We assessed registrations under the binary sum-of-squares metric in combination with an evolutionary optimizer.

Two further approaches utilizing virtual staining (*VS/feature* and *VS/xcorr*) are described and validated in Additional file 2: Section A.3.

Table 1 shows an overview of the samples available for evaluation. For samples (S6–S9), both the spectral image and the stained image are acquired as whole-slide images. As the availability of whole-slide FTIR images is rather exceptional due to the high demand in measurement time and memory, we randomly selected large numbers of subregions of the whole-slide FTIR image to assess our subregion registration approach. A correct reference registration for these randomly selected subregions can be obtained conveniently by registering the whole-slide FTIR image against its H&E counterpart. For the remaining five samples (S1–S5), spectral images were limited to several ROIs of each respective sample. Each stained image is resized according to the resolution of the spectral image and the actual size of the tissue. For samples S1 and S2, manual preregistrations were available as ground truth. For samples S3–S5, it was possible to unambiguously judge each registration position as correct or incorrect by visual inspection.

**Table 1:** Overview of the samples used for validation

Id	Type	Size (pixels)		Number and sizes of spectral images
S1	Colon	(1915×994)	7	(336×128), (240×128), (240×128), (240×128), (240×128), (384×96), (288×160)
S2	Colon	(2402×1872)	3	(240×160), (192×128), (288×128)
S3	Colon	(1203×987)	6	(288×128), (288×160), (192×128), (288×128), (192×32), (192×32)
S4	Colon	(2666×1720)	5	(288×128), (336×128), (96×488), (288×128), (96×488)
S5	Bladder	(319×257)	1	(192×192)
S6	Bladder	(3000×3615)	1	(2816×2432)*(whole slide)*
S7	Lung	(5334×4000)	1	(3840×3712)*(whole slide)*
S8	Colon	(3946×4200)	1	(2304×2688)*(whole slide)*
S9	Colon	(4500×3551)	1	(4096×3804)*(whole slide)*
S10–S15	Lung	(1392×886)– (2491×2227)	2–5	(212×354)– (618×237)
S16–S28	Bladder	(1639×947)– (2985×1676)	3	(212×354)– (618×237)
TMA	Colon		56 cores	∼(375×375)*(approx. size of one spot)*
C1	Lung	(825×825)	1	(1024×1024)
C2	Lung	(1473×1845)	1	(1024×1024)

Spectral images for samples S1–S28 and TMA are available as FTIR images, while spectral images for samples C1 and C2 are CARS microscopic images. For samples S1–S28 and TMA, the resolution of the H&E images has been adjusted to approximately match the resolution of the corresponding FTIR images. Note that for the CARS samples C1 and C2, the spatial resolution of the CARS images is higher than the resolution of the H&E images, so that the CARS subregions involve significantly more pixels than the H&E images, which cover a much larger area. This difference in resolution for CARS is resolved by taking into account scaling during registration. Full details on samples S10–S28 are provided in Additional file 1: Table S1

If not explicitly stated otherwise, all evaluation results refer to default parameters which were set as follows. The number of clusters was set to ten in both the stained and the spectral image whenever registering full images (or complete TMA cores). Whenever dealing with a template matching scenario, the stained images were segmented into ten clusters, while the smaller spectral image was segmented into eight clusters. As it turns out, our approach is robust across a large range of cluster number values (see Additional file 1: Table S3). The optimizer involves two parameters *k*_0_ and *δk* (see Additional file 2: Figure S3), which were set to 0.6 and 0.5 respectively, and the initial radius *r*_0_ was estimated based on self- registration as described above. The adjustment factor for restricted mutual information was set to *α*=0.25, which is within a range observed to be robust across a large set of samples (Additional file 1: Table S4). The default limits of the scaling factor are [0.8,1.2], and rotations were limited to [−180,180] for full registration and [−30,30] for template matching.

### FTIR subregion registration

**Fig. 4:**
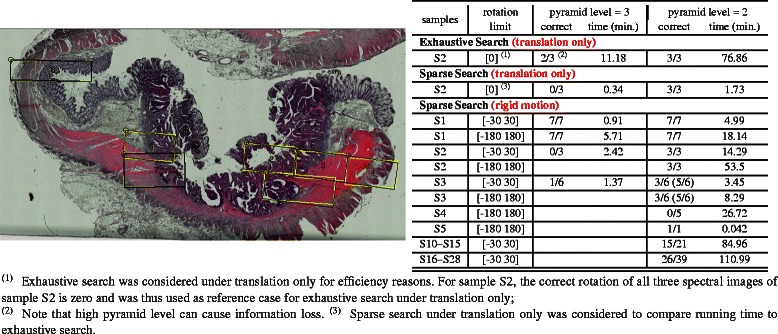
*Left*. Registration result of seven regions-of-interest in Sample S1 using *RMI/sparse*. Yellow rectangles indicate the manually registered location, black rectangles indicate the registration result. *Right*. Assessment of registration accuracy of regions of interest in samples S1–S5. The high running time for exhaustive search on sample S2 despite limiting transformations to translation only demonstrates the need for improved search methods

We validated our approach on the nine whole-slide samples S1–S9 listed in Table 1. In five cases (S1–S5), the FTIR image was captured for one or few regions of interest as subregions of the complete slide. For the remaining cases, the complete slides were captured in the FTIR image. In the latter case, we extracted a large number of random cutouts of two different sizes (320×180 and 640×360 pixels) at different positions and small angels of rotation and reconstructed the positions of these random cutouts using our registration procedure. Corresponding registration results are shown in Fig. 4, indicating both the robustness of RMI/sparse compared to MS/gradient as well as the efficiency advantage of the sparse search optimizer: while exhaustive search exceeds acceptable running times under rigid motions and can thus be evaluated only under translations, the registration times for the sparse search optimizer under rigid motions are within the order of minutes for each ROI to be registered.

The registration accuracies were measured through regression errors as shown in Additional file 2: Section A.2.3. Corresponding errors of manual registration and our *RMI/sparse* approach are compared in Additional file 1: Table S2. As it turns out, the results of automated registration can be considered more accurate than manual registration when defining registration accuracy based on regression errors (also demonstrated in Additional file 1: Figure S4).

**Fig. 5:**
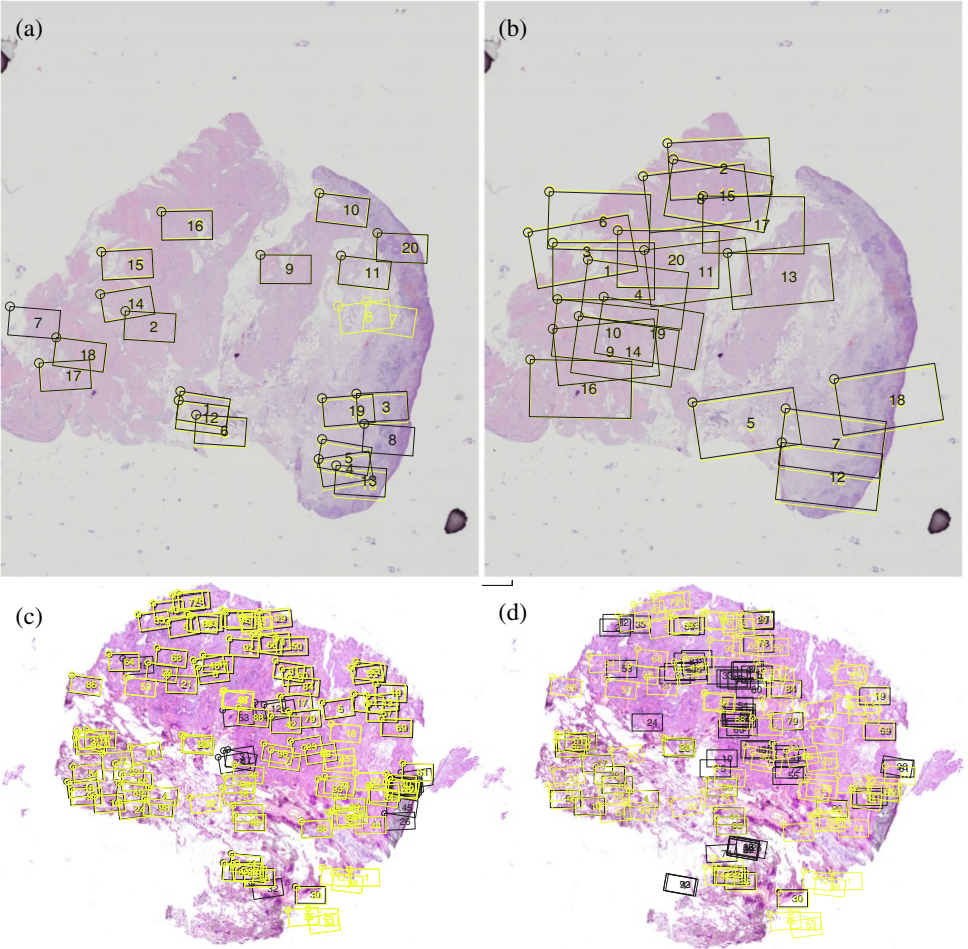
Results of subregion registration of random cutouts. Yellow rectangles indicate the ground truth registration location, black rectangles indicate the registration result. Results of subregion registration of random cutouts in (**a**) cutouts of size 180×320 pixel spectra in sample S6; (**b**) cutouts of size 360×640 pixel spectra in S6. Panel (**c**) shows Subregion registration using of our pipeline (84 regions registered correctly). Panel (**d**) shows registration results of the *binary MS/evo* approach for the same 100 random cutouts (32 regions registered correctly). A corresponding comparison involving further methods and random cutouts of three samples is shown in Additional file 2: Table S1

For sample S7, we extracted 100 such random cutouts (320×180 in size) and compared the performance of our approach with the *MS/gradient* approach proposed previously for registering TMA cores. As it turns out, our *RMI/sparse* approach registers 84 out of these 100 regions correctly, whereas the *binary MS/gradient* approach registers only 32 cores correctly, see Fig. 5. *RMI/sparse* also turns out to be by far more robust than feature based approaches or correlation-based approaches using virtual staining, as shown in Additional file 2: Figure S7 and Additional file 2: Table S1.

To assess robustness, we examined the results under different numbers of clusters used in preprocessing and under varying the adjustment factor *α* of the restricted mutual information. An evaluation on 16 subregions in four samples shows that registration works reliably for an adjustment factor *α* in the range between 0.2 and 0.4 (Additional file 1: Table S4). Also, *RMI/sparse* works reliably under a large range of values for *k* around the default choice of *k*=10 (Additional file 1: Table S3).

### Registering tissue microarray cores

**Fig. 6:**
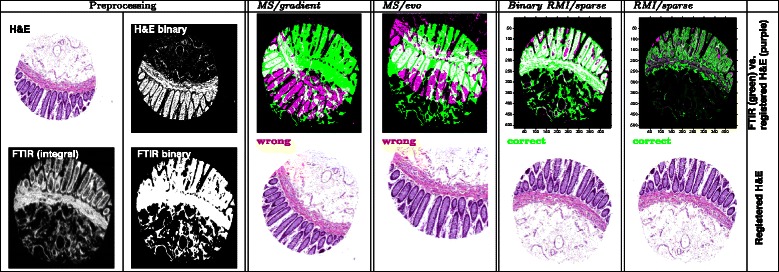
Registration result of different methods for a single tissue microarray spot. The spot diameter is roughly 1.5 mm, and registration fails for both MS/gradient and MS/evo, but works successfully for our newly proposed Binary RMI/sparse and RMI/sparse. Results on all 56 TMA spots are summarized in the text and detailed in Additional file 3. In the first row of columns 3–6, green indicates foreground area in the FTIR image and purple indicates foreground area in the H&E image, so that white areas indicate foreground overlap between the registered images

We considered 56 cores captured as both FTIR and H&E image from one TMA. Among the 56 tissue microarray spots under consideration, the *binary MS/gradient* approach produced 15 wrong registrations. Replacing the gradient optimizer by an evolutionary optimizer in *binary MS/evo* yielded 3 wrong results (Additional file 3). We compared these results with our approaches *RMI/sparse* with *k*=10 clusters and *binary RMI/sparse*. In both scenarios, one out of the 56 cores was registered incorrectly. A detailed result for one sample spot is shown in Fig. 6. For detailed results we refer to Additional file 3.

These results detailed in Additional file 3 suggest that for the registration of TMA cores in general a relatively simple approach is viable on the grounds of a binary foreground-background segmentation. However, the more sophisticated target function and optimizer utilized in our proposed *RMI/sparse* framework (55 out of 56 correctly registered) yields correct registrations more reliably than the previously proposed *binary MS/gradient* (41 out of 56 correctly registered).

### CARS subregion registration

We applied our *RMI/sparse* registration pipeline on a subregion registration of a spectral image obtained by a CARS microscope with the corresponding H&E image of the same sample. Due to the high spatial resolution of CARS, the region of interest contained in the spectral image is much smaller, covering an area of roughly 300 *μm* squared with 1024×1024 pixel spectra. For CARS spectra, it is in general difficult to identify spectra belonging to positions not covered by sample and thus representing substrate only. H&E images were available with a pixel resolution of 2 *μm* and cover an area several times larger than the ROI measured by CARS. Registration based on a binary foreground-background segmentation is not possible in this case for two reasons — first, due to the lack of a systematic approach to identify background spectra in CARS images; and second, due to the small size of the ROIs which may in fact be completely covered by sample and thus not contain any background positions at all.

**Fig. 7:**
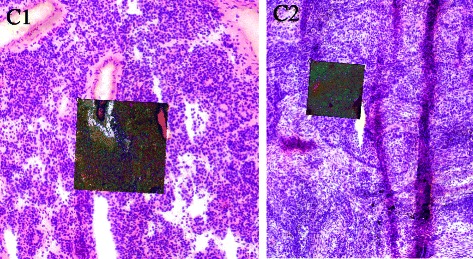
Registration of the four CARS microscopically captured regions of interest within H&E stains of the same respective sample obtained from lung tissue

The spectrally measured ROI needed to be registered against a region several times larger in the H&E image (3364×2876 pixels). The index image of the spectral image is obtained from performing *k*-means clustering on the image spectra without further preprocessing. The result of registration under similarity transformations (i.e., translation, rotation, and scaling) is displayed in Fig. 7, illustrating the successful registration result.

## Conclusion

We have demonstrated that our proposed *RMI/sparse* approach allows robust, reliable and efficient registration even in the scenario of matching subregions and across different microscope constellations involving vibrational microspectroscopic images. The robustness of our approach across different sample types, different microscopes, as well as changes in the parameters involved, provides strong support that our method will be applicable in a straightforward manner to data collected in other labs and studies. This significantly simplifies the application of vibrational microspectroscopy not just in the context of spectral histopathology, where registration is an elementary step for training spectral classifiers on a new tissue type.

Achieving both efficiency and robustness required a fair degree of adapting or improving existing approaches towards image registration. In this context, there are several novelties in our contribution. First, we could demonstrate that performing registration using clustering-based presegmentations of the image modalities involved is an important *key for reliable registration*. This simple but crucial observation may in fact be helpful if not indispensable for registration scenarios involving other, in particular multispectral or hyperspectral microscopy modalities. A second contribution from the computational side is to utilize restricted mutual information as a registration metric. Thirdly, as a further methodological contribution, we could demonstrate that sparse grid based optimizers are *key towards efficiency* when dealing with high resolution, tissue sample sized data. While the sparse search optimizer itself is straightforward, our self-registration based estimation of the initial grid radius is *key to facilitate fully automated registration* without requiring users to adjust parameters. In summary, achieving the three-fold goal of a robust, efficient and fully automated method required the extension of existing registration approaches.

These methodological contributions promise to be of value beyond the scenarios investigated in our contribution. Considering trends towards further diversification of microscopy technology along with a simultaneously increasing trend to study samples across different microscopy platforms, it is well conceivable that new registration challenges will emerge. For such settings, a conceptual lesson learned from our work is that all components of a registration approach – preprocessing, registration metric, optimizer and interpolator – need to go hand in hand to achieve the required degree of reliability. This will hold in particular for settings dealing with the registration of samples from consecutive sections rather than using the identical sample, which will exhibit non-linear differences in morphology. The same also holds for registration problems dealing with advanced alignments of three-dimensional data sets, which have gained relevance in studying large-scale neural systems such as whole brain images [29, 30] or atlases of model organisms [31].

Recently, several other concrete scenarios dealing with a combination of two microscopy techniques applied to one and the same sample have been investigated. In fact, such scenarios have been explicitly recognized as a potential source of improved understanding of processes in biological samples [1]. Whenever two microscopy platforms are involved, registration immediately becomes of relevance, as the sample will need to be moved physically from one microscope to the other. Reliable registration algorithms greatly simplify experimental protocols, which otherwise need to introduce physical landmarks on the sample, which raises technical and experimental problems. Correspondingly, Masyuoko et al. [1] conclude that “for maximal utilization of multimodal imaging data it is crucial to develop efficient solutions for cross-platform sample registry”. Our results strongly suggest that our *RMI/sparse* registration framework will allow reliable registration results in other constellations of microscopes for studies working across different microscopy platforms. In fact, our approach works on the level of index images obtained by rough clustering based presegmentations, which are relatively easy to obtain for other currently popular microscopic platforms, in particular multi-spectral and hyperspectral ones, such as Raman microscopy and MALDI imaging [6].

## Ethics, consent and permissions

The present study was in accordance with the ethical standards of the Committee on Human Experimentation of the University of Bochum Register Nrs. 4073-11, 3674-10, and 4552-12.

## Availability

An implementation of our approach can be downloaded from http://www.bioinf.rub.de/software/index.html.en and https://github.com/yangchen-picb/SPECREG.

## Additional files

Additional file 1
**Supplementary Figures and Tables.** **Table S1.** Complete overview of the samples used for validation. **Figure S1.** Correlation between FTIR spectra and staining colors measured by the rank correlation of the difference map. **Figure S2.** Feature detection of images using the SIFT detector. **Figure S3.** Color correction of H&E stained images. **Table S2.** Registration Accuracies. **Table S3.** Registration robustness under varying numbers of clusters used for computing presegmentations. **Table S4.** Registration robustness under varying adjustment factor *α*. **Figure S4.** Registration is more accurate than manual registration. (PDF 3584 kb)

Additional file 2
**Algorithmic Details.** **Figure S1.** Restricted Mutual Information. **Figure S2.** Sparse Search Optimizer and Initial Radius Estimation. **Figure S3.** Virtual Staining based Registration. (PDF 4618.24 kb)

Additional file 3
**Tissue Microarray Registration Results.** Detailed registration results of 56 FTIR vs. H&E TMA cores. (PDF 46387.2 kb)

## Abbreviations

FTIR: Fourier transform infrared
H&E: Hematoxylin an eosin
CARS: Coherent anti-stokes raman Spectroscopy
MALDI: Matrix-assisted laser desorption/ionization
MALDI-TOF: Matrix-assisted laser desorption/ionization-time of flight
TMA: Tissue microarray
SIMS: Secondary ion mass spectrometry
ROI: Region of interest
SHP: Spectral histopathology
MI: Mutual information
RMI: restricted mutual information
MS: Mean-square
VS: Virtual staining
