# Supplementary material for: Fully automated registration of vibrational microspectroscopic images in histologically stained tissue sections
Source: BMC Bioinformatics. 2015 Nov 25;16:396. doi: 10.1186/s12859-015-0804-9 (PMC4659215; doi:10.1186/s12859-015-0804-9)
Supplement: Additional file 1 — Supplementary Figures and Tables. Table S1. Complete overview of the samples used for validation. Figure S1. Correlation between FTIR spectra and staining colors measured by the rank correlation of the difference map. Figure S2. Feature detection of images using the SIFT detector. Figure S3. Color correction of H&E stained images. Table S2. Registration Accuracies. Table S3. Registration robustness under varying numbers of clusters used for computing presegmentations. Table S4. Registration robustness under varying adjustment factor α. Figure S4. Registration is more accurate than manual registration. (PDF 3584 kb) [file 12859_2015_804_MOESM1_ESM.pdf]

Registration of Infrared Microscopic Images in Histologically Stained Tissue Sections using Restricted Mutual Information and Sparse Search

Additional File 1

Supplementary Figures

Chen Yang, Daniel Niedieker, Frederik Großerueschkamp,
Melanie Horn, Angela Kallenbach-Thieltges,
Klaus Gerwert, Axel Mosig

Supplementary Table S1. Complete overview of the samples used for validation. Spectral images for samples S1–S28 and TMA are available as FTIR images, while spectral images for samples C1 and C2 are CARS microscopic images. For samples S1–S28mayerich2015stain and TMA, the resolution of the H&E images has been adjusted to approximately match the resolution of the corresponding FTIR images. Note that for the CARS samples C1 and C2, the spatial resolution of the CARS images is higher than the resolution of the H&E images, so that the CARS subregions involve significantly more pixels than the H&E images, which cover a much larger area. This difference in resolution for CARS is resolved by taking into account scaling during registration.

id	type	size (pixels)	number and sizes of spectral images	
S1	colon	(1915×994)	7	(336×128), (240×128), (240×128), (240×128), (240×128), (384×96), (288×160)
S2	colon	(2402×1872)	3	(240×160), (192×128), (288×128)
S3	colon	(1203×987)	6	(288×128), (288×160), (192×128), (288×128), (192×32), (192×32)
S4	colon	(2666×1720)	5	(288×128), (336×128), (96×488), (288×128), (96×488)
S5	bladder	(319×257)	1	(192×192)
S6	bladder	(3000×3615)	1	(2816×2432) *(whole slide)*
S7	lung	(5334×4000)	1	(3840×3712) *(whole slide)*
S8	colon	(3946×4200)	1	(2304×2688) *(whole slide)*
S9	colon	(4500×3551)	1	(4096×3804) *(whole slide)*
S10	lung	(1379×1535)	2	(511×227), (339×228)
S11	lung	(2491×2227)	5	(618×237), (417×284), (431×283), (391×381), (449×260)
S12	lung	(1505×1283)	3	(367×215), (357×182), (212×354)
S13	lung	(2798×1280)	5	(591×212), (599×191), (522×223), (655×182), (429×324)
S14	lung	(1392×886)	4	(422×166), (381×151), (348×156), (353×155)
S15	lung	(1520×1347)	2	(439×175), (227×249)
S16	bladder	(1961×1488)		(333×113), (417×132), (368×166)
S17	bladder	(2985×1676)		(414×340), (505×291), (646×259)
S18	bladder	(1450×1343)		(455×194), (512×210), (157×485)
S19	bladder	(1599×1688)		(438×196), (513×190), (495×177)
S20	bladder	(1816×1247)		(337×161), (402×196), (495×191)
S21	bladder	(1639×947)		(421×151), (444×129), (480×124)
S22	bladder	(1569×1391)		(414×136), (461×163), (392×183)
S23	bladder	(1864×1682)		(488×194), (454×266), (307×576)
S24	bladder	(1760×1352)		(516×205), (564×230), (388×182)
S25	bladder	(1794×1935)		(602×196), (578×223), (494×205)
S26	bladder	(1999×2103)		(373×544), (714×272), (677×264)
S27	bladder	(1804×1673)		(545×192), (546×220), (493×278)
S28	bladder	(1844×995)		(392×144), (495×173), (434×180)
TMA	colon		56 cores	$\sim (375 \times 375)$ *(approx. size of one spot)*
C1	lung	(825×825)	1	(1024×1024)
C2	lung	(1473×1845)	1	(1024×1024)

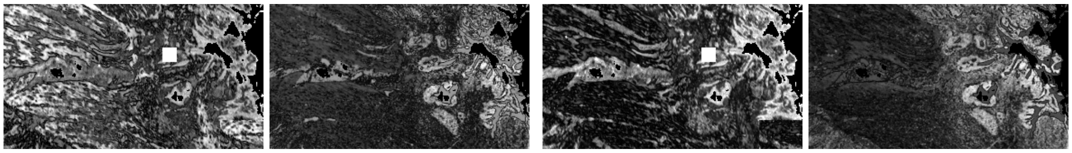

(a) spearman $\rho = 0.1662$ (b) spearman $\rho = 0.2773$

Supplementary Figure S1. *Correlation between FTIR spectra and staining colors measured by the rank correlation of the difference map (signal distances to a reference signal).* (a) Difference map to the mean spectra (left) and mean color (right); (b) difference map to the spectra/color of a corresponding point.

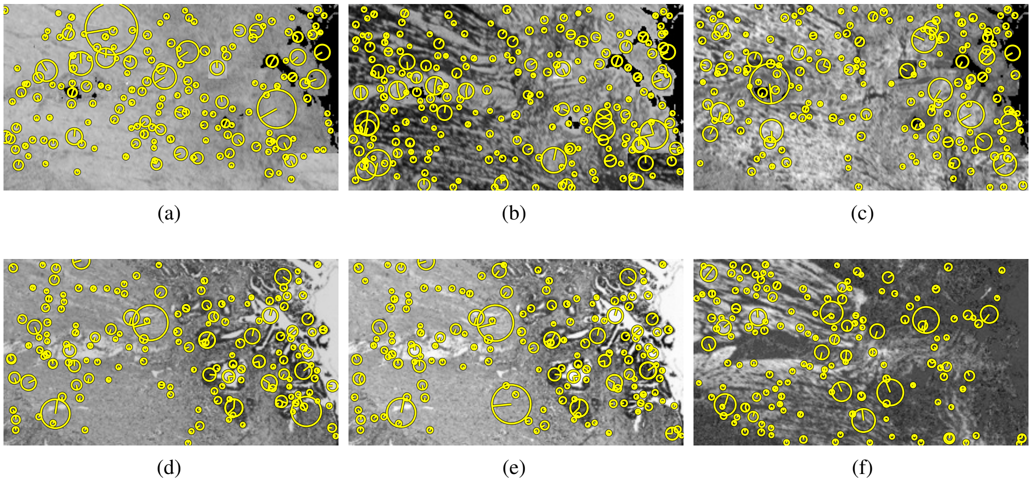

Supplementary Figure S2. *Feature detection of images using the SIFT detector [1] (difference of Gaussians).* (a) The most informative channel of the spectral image; (b) the first principal component of the spectral image; (c) the second principal component of the spectral image; (d) gray-scale stained image; (e) the first principal component of the stained image; (f) the second principal component of the stained image. The correspondence between feature points in the spectral image (top row) and feature points in the stained image (bottom row) is barely recognizable. Consequently, registration algorithms based on feature points will likely fail to yield correct registration results, motivating our choice to investigate area- based rather than feature-based approaches.

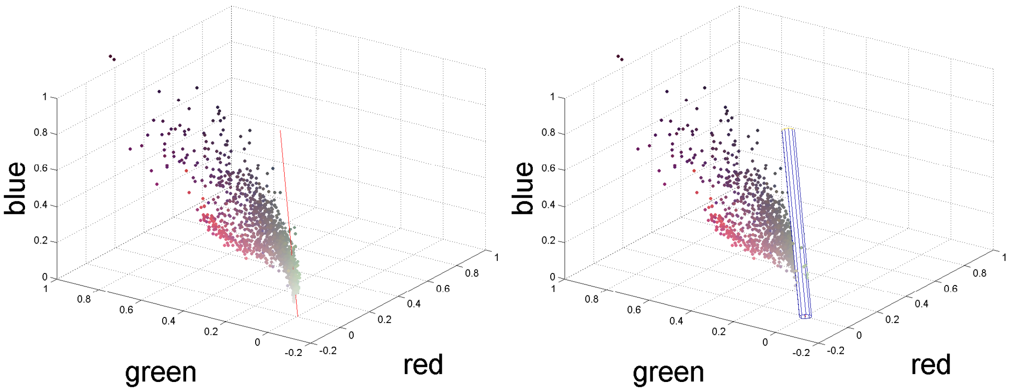

(a)

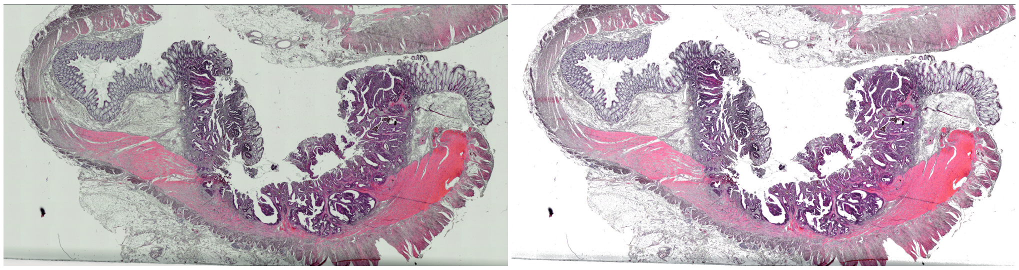

(b)

Supplementary Figure S3. *Color correction of the H&E stained image.* (a) Randomly sampled pixel color in optical density space [2]. The red line (left image) indicates the color vector identified as background, while the cylinder (right image) indicates the corresponding threshold used for separating background. (b) The stained image before (left) and after (right) color correction.

Supplementary Table S2. *Registration Accuracy.* Mean regression distance error (RMDE, refer to Supplement A.2.3) tends to be slightly lower for automated registration using our pipeline (middle column) compared to manually obtained ground truth (left column), suggesting that registration results obtained from our pipeline are more accurate than manual registration.

samples	RMDE		TMDE
	manual	pipeline	(pixels)
S1-1	45.43	42.02	3.96
S1-2	59.84	52.49	2.84
S1-3	33.52	33.09	5.52
S1-4	37.24	35.05	5.52
S1-5	42.01	38.12	5.26
S1-6	36.93	**38.27**	0.88
S1-7	37.04	36.85	1.21
S2-1	26.04	25.62	1.68
S2-2	28.75	27.14	0.85
S2-3	30.17	28.28	0.73

samples	TMDE (pixels) when number of clusters of pre-segmented stained image =															
	5	6	7	8	9	10	11	12	13	14	15	16	17	18	19	20
S1-1	0.66	1.16	0.88	0.66	0.66	0.00	1.16	0.70	0.82	0.66	0.75	0.82	0.95	0.75	0.95	0.95
S1-2	0.75	0.00	0.75	0.76	0.00	0.76	1.50	0.00	0.76	0.66	0.66	0.66	0.66	0.66	0.66	0.66
S1-3	1.41	1.00	0.00	1.41	1.30	1.91	1.91	1.41	1.41	2.92	2.58	1.62	2.92	3.52	1.62	1.62
S1-4	0.76	3.22	0.84	0.00	2.25	0.00	0.00	0.00	0.00	1.00	0.00	0.84	0.00	0.84	0.84	0.84
S1-5	1.06	2.11	2.11	2.11	0.75	0.75	0.84	1.63	1.06	1.78	1.78	2.36	1.63	1.78	1.63	0.75
S1-6	1.41	3.72	1.79	2.58	1.79	2.74	2.74	2.74	2.74	2.95	**1561.20**	2.74	2.74	2.95	2.74	2.74
S1-7	0.00	0.00	0.00	0.00	0.85	0.00	0.00	0.85	0.85	0.85	0.85	0.00	0.00	0.85	0.85	0.85
S2-1	2.32	2.32	2.32	0.00	0.00	0.00	0.00	0.00	3.60	0.00	0.00	0.00	0.00	0.00	0.00	0.00
S2-2	2.05	1.98	1.98	0.00	0.00	0.00	0.00	0.00	0.00	0.00	0.00	0.00	0.00	0.00	0.00	0.00
S2-3	0.00	4.01	3.15	0.00	0.00	0.00	0.00	0.00	0.00	0.00	**293.18**	0.00	1.53	1.58	0.00	0.00
S3-1	**622.99**	**625.41**	0.90	2.22	2.22	1.53	2.69	0.95	1.83	0.95	1.99	0.95	0.90	0.95	0.89	0.89
S3-2	**387.48**	0.81	**615.32**	0.81	0.81	0.81	0.00	1.53	0.90	1.16	0.00	0.95	1.41	0.00	0.89	0.81
S3-4	0.00	1.41	0.00	0.00	1.49	1.43	0.00	0.91	1.41	0.95	1.41	0.00	0.00	0.00	0.89	1.60
S3-5	**309.12**	**302.70**	0.00	2.83	1.38	1.79	2.83	1.38	0.85	4.50	**118.09**	0.00	**735.46**	**116.03**	**322.88**	**752.76**
S3-6	**113.31**	**361.97**	1.00	1.49	0.00	2.63	3.44	0.72	1.49	**114.69**	1.00	0.94	2.83	3.92	**115.74**	**80.37**
S5-1	0.00	0.00	0.00	0.00	0.00	0.00	0.00	0.00	0.00	0.00	0.00	0.00	0.00	0.00	0.00	0.00

Supplementary Table S3. *Registration robustness under varying numbers of clusters used for computing presegmentations.* Small deviations in terms of the TMDE (refer to Supplement A.2.3 for a definition) indicate deviations of only few pixels from a generally correct registration result. Bold face entries indicate positions that produce generally wrong results. Note that for the range of $8 \leq k \leq 13$ many clusters, registration is correct in all 16 cases.

Supplementary Table S4. *Registration robustness under varying adjustment factor* α. Small deviations in terms of the TMDE (refer to Supplement A.2.3 for a definition) close to 0 indicate deviations of only few pixels from a registration result that can be considered correct in general. Bold face entries indicate positions that produce results to be considered principally wrong. Note that for the range of $.2 \leq \alpha \leq .4$, registration is correct in all 16 cases.

samples	TMDE (pixels) when adjustment factor =					
	0	0.1	0.2	0.3	0.4	0.5
S1-1	1.54	0.75	0.66	0.66	0.66	0.66
S1-2	0.27	0.27	0.71	0.13	0.13	0.71
S1-3	1.29	2.25	0.88	0.88	0.13	2.02
S1-4	0.27	0.27	0.87	0.91	0.87	0.87
S1-5	0.84	0.82	0.13	0.84	0.84	0.84
S1-6	0.27	2.55	3.35	2.55	3.79	6.10
S1-7	0.00	0.00	0.00	0.00	0.00	0.00
S2-1	**685.84**	**687.46**	0.00	0.00	0.00	0.00
S2-2	0.00	0.00	0.00	0.00	0.00	0.00
S2-3	**311.05**	0.00	0.00	0.00	2.35	**305.36**
S3-1	**632.39**	0.00	0.00	0.89	0.00	**564.83**
S3-2	**678.38**	0.00	1.00	0.81	0.81	1.41
S3-4	**654.19**	**640.75**	0.00	0.00	2.29	2.24
S3-5	0.00	0.00	0.00	0.00	1.85	3.50
S3-6	**922.22**	1.00	1.00	1.41	0.00	0.72
S5-1	0.00	0.00	0.00	0.00	0.00	0.00

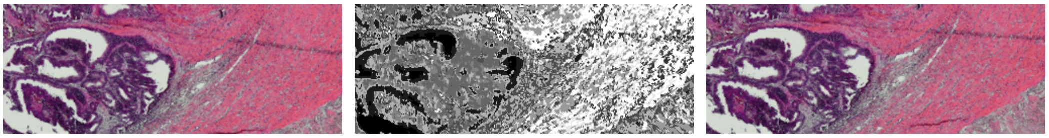

(a) (b) (c)

Supplementary Figure S4. *Registration is more accurate than manual registration.* (a) Cutout of the registered region within the stained image according to manual ground-truth registration; (b) the index image of the spectral image to be registered; (c) the cutout image of the registered region within the stained image according to automated registration based on our newly proposed method (*RMI/sparse*).

References

[1] David G. Lowe. Distinctive Image Features from Scale-Invariant Keypoints. *International Journal of Computer Vision*, 60(2):91–110, November 2004.

[2] Marc Macenko, Marc Niethammer, J. S. Marron, David Borland, John T. Woosley, Charles Schmitt, and Nancy E. Thomas. A method for normalizing histology slides for quantitative analysis. In *2009 IEEE International Symposium on Biomedical Imaging: From Nano to Macro*, pages 1107–1110. IEEE, June 2009.
