# Supplementary material for: Fully automated registration of vibrational microspectroscopic images in histologically stained tissue sections
Source: BMC Bioinformatics. 2015 Nov 25;16:396. doi: 10.1186/s12859-015-0804-9 (PMC4659215; doi:10.1186/s12859-015-0804-9)
Supplement: Additional file 2 — Algorithmic Details. Figure S1. Restricted Mutual Information. Figure S2. Sparse Search Optimizer and Initial Radius Estimation. Figure S3. Virtual Staining based Registration. (PDF 4618.24 kb) [file 12859_2015_804_MOESM2_ESM.pdf]

Fully Automated Registration of Vibrational Microscopectroscopic Images in Histologically Stained Tissue Sections

Additional File 2

Chen Yang, Daniel Niedieker, Frederik Großerüschkamp,
Melanie Horn, Angela Kallenbach-Thieltges,
Klaus Gerwert, Axel Mosig

Supplement A.1 Restricted Mutual Information

A.1.1 Mutual Information

Due to its ability to capture nonlinear dependencies between random variables, mutual information as an information theoretic measure of correlation is particularly suitable and well-established for the registration of images across different image modalities [4]. Several variants of mutual information measures have been proposed in the context of image registration, most notably Viola-Wells mutual information [6] and Mattes mutual information [2]. In our setting we employ discrete mutual information in the spirit of Mattes mutual information, where only indices of segments obtained from an unsupervised presegmentation of the images are directly used rather than binning a continuous random variable.

Utilizing discrete mutual information between the index images as our similarity measure, we capture the hidden correlation between the FTIR spectral image and H&E stained image to some extent. However, the precise relationship remains unknown, causing the correlation between the two index images to be rather weak in general (Figure 2 in the main manuscript).

When using this weak correlation in a template-matching-like registration as our similarity measure, we encounter the problem of *background attraction* exemplified in Figure S1. It can in fact be observed that background regions generally achieve a relatively high, often globally maximal, mutual information during template matching. This is mainly because the correlation between two images in the true transformation is relatively weak, while a large plain region (which is a common characteristic of background regions) can significantly reduce the joint entropy, making the mutual information bigger than the regions that are totally uncorrelated. This phenomenon is particularly prominent when employing image pyramid schemes [5], where the mutual information at higher pyramid levels is strongly affected by this phenomenon.

Background attraction prohibits correct registration results in many cases for mainly two reasons. First, sometimes mutual information achieves its maximum value at the wrong transformation, leading to false registration positions (Figure S4a). Second, whenever not using exhaustive search to determine the transformation that maximizes mutual information, the optimizer may be trapped in a local, but not global optimum. Note that background regions typically yield large segments in clustering based presegmentations, and thus are particularly prone to produce local maxima of large width in the scoring landscape. Thus, even if the global maximum is at the correct position, many types of optimizers may easily get trapped in the wide local maxima of background regions. Even if the optimizer can finally escape, it will require a large number of evaluations around the false optimum, with consequently severe impact on the running time.

To address these problems, we introduce the concept of *restricted mutual information* for our metric, which explicitly deals with background in both the FTIR and the H&E image.

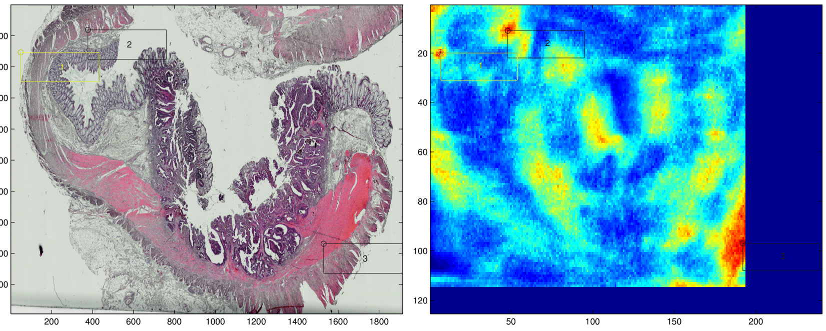

(a) (b)

Supplementary Figure S1. *Illustration of the background attraction phenomenon under 2D translation using pyramid level 3.* (a) The three rectangular boxes indicate three transformations that yield high mutual information; (b) the heat map of mutual information in transformation space.

A.1.2 Background as extra information in registration

Background in microscopic tissue sections can generally be referred to as all those areas of the microscopy slide that are not covered by tissue. Clearly, background areas in the two images should be matched to each other in a correct transformation, and matching foreground in one image with background in the other image should be avoided for correct registration results. For some extreme cases, background information alone can be sufficient for registration.

In this context, note that for both the FTIR image and H&E stained image, a corresponding foreground-background segmentation can typically be obtained easily as described in the main manuscript. With the help of background information, we can efficiently filter out the false transformations and also avoid background attraction. In a simplistic manner, this could for instance be achieved through hard thresholds on the overlap between foreground and background parts of the images. As shown in Figure 2 in the main manuscript, however, the segmentation of both the stained image and spectral image can be inaccurate. Thus, we seek to incorporate explicit background information into a mutual-information based metric in a more systematic manner in the following subsection.

A.1.3 A matching perspective of mutual information

In order to incorporate background information into a mutual information scheme, we take a perspective on mutual information that slightly differs from the conventional information theoretic interpretation. While originating from coding theory, mutual information as used in image registration can be considered *the amount of information that one image provides about the second image* or *the amount by which the uncertainty about one image decreases when the second image is given*, and can thus be regarded as a meaningful measure of image resemblance. On an intuitive level, our adapted version of mutual information relies on a slightly different perspective, namely consid-

ering mutual information as a measure of *how good the two images match each other*.

To explain this, we follow the definition of the discrete entropy [1]

$$H(X) \quad = \quad -\sum_{x \in \mathscr{X}} p(x) \log p(x), \tag{1}$$

where $p(x) = P(X = x), x \in \mathscr{X}$ is the probability mass function of a discrete random variable X with alphabet $\mathscr{X}$.

Suppose the distribution of X, $P(X = x)$ is estimated by a sample vector A containing m samples. In the scenario of image registration, A is an index image comprising m pixels and $|\mathscr{X}|$ many index values, so that the sample vector A is a mapping $A\colon [1 : m] \to \mathscr{X}$. With the sample vector A, the estimated probability of an empirical distribution of X is

$$P(X = x) \quad = \quad \#\{i \in [1 : m] \mid A(i) = x\}/m. \tag{2}$$

Combining (1) and (2), the entropy can be rewritten as

$$\begin{aligned} H(X) &= -\frac{\sum_{i=1}^{m} \log P(X = A(i))}{m} \\ &= \frac{-\log \prod_{i=1}^{m} P(X = A(i))}{m} \end{aligned} \tag{3}$$

In other words, entropy can be interpreted as the *average negative log likelihood* of the sample assuming it comes from the empirical distribution estimated by the sample itself.

Following the same deduction as (3), we can now rewrite joint entropy as

$$\begin{aligned} H(X,Y) &= -\sum_{x \in \mathscr{X}} \sum_{y \in \mathscr{Y}} p(x,y) \log p(x,y) \\ &= \frac{-\log \prod_{i=1}^{m} P(X = A(i), Y = B(i))}{m}, \end{aligned}$$

when introducing a second random variable $Y = (P, \mathscr{Y})$ with the sample vector $B\colon [1 : m] \to \mathscr{Y}$.

Here, the logarithms of the probabilities

$$p(x,y) = P(X = A(i), Y = B(i))$$

can be regarded as a matching score of the pair $(A(i), B(i))$, so that the values $\log p(x,y)$ are the main contributors to the score in our matching based perspective on mutual information. In other words, the joint distribution is a “scoring scheme” for all pairs of jointly observed index values. The joint entropy is the negative matching score (or penalty function) of two vectors, so that two perfectly matched vectors always have minimum joint entropy, i.e., zero – even in the case of two plain white images. In other

words, the joint entropy corresponds to the matching score using the scoring scheme which is estimated by the matching itself (i.e., the empirical distribution).

Now, we consider the mutual information

$$\begin{aligned} I(X,Y) &= \sum_{x\in\mathscr{X}}\sum_{y\in\mathscr{Y}} p(x,y)\log\frac{p(x,y)}{p(x,y)} \\ &= H(X)+H(Y)-H(X,Y), \end{aligned} \tag{4}$$

where we can regard the entropy or uncertainty measures $H(X)$ and $H(Y)$ as matching difficulties, and $-H(X,Y)$ as matching quality.

Taking a Bayesian perspective, we define the event M as A *and* B *are matched*:

$$\begin{aligned} P(M|A,B) &= \frac{P(A,B|M)P(M)}{P(A,B)} \\ &\propto \frac{P(A,B|M)}{P(A)P(B)} \\ &= \frac{\prod_{i=1}^{m} P(X=A(i),Y=B(i))}{\prod_{i=1}^{m} P(X=A(i))\prod_{i=1}^{m} P(Y=B(i))} \\ &= \prod_{i=1}^{m}\frac{P(X=A(i),Y=B(i))}{P(X=A(i))P(Y=B(i))}. \end{aligned} \tag{5}$$

The logarithm of (5) is the mutual information (4). This means mutual information can be seen as the matching probability of two sample vectors.

In order to adapt mutual information towards a restricted version suitable to infrared-H&E image registration, we view mutual information as a substitution scoring scheme in the sense of nucleotide or amino acid substitution schemes for biological sequence alignments. Standard mutual information as a scoring scheme then is based on the observed probabilities, which are all obtained from the observed data. In this sense, mutual information works as follows: the more frequently a co-occurring index pair occurs, the higher this pair will be scored, which finally yields the nonlinear correspondence scheme of mutual information. The more frequently a single value occurs in one vector, the lower the score of a pair involving this value will get, thus avoiding a high score for a random coincidental match.

A.1.4 Restricted Mutual Information

By taking a matching perspective on mutual information as described above, we now introduce the restricted version of mutual information to facilitate improved infrared-H&E registration. To this end, note that whenever we want to reward a frequently occurring matching index pair, we may lose prior knowledge that certain index pairs should be rewarded separately. In the case of infrared-H&E registration, this is typically the case for background positions: In the presegmentation of both the H&E and the infrared image, background is clearly identifiable, and for obvious reasons background in the infrared image should be matched with background in the H&E image. Thus, we will now adapt the scoring scheme/joint probability to achieve this. After the

joint probability has been estimated by the samples, we assume that positions $A(i) = 0$ in the infrared $B(i) = 0$ in the H&E image represent background. Correspondingly, we can increase the matching score for the probability $P(X = 0, Y = 0)$ while proportionally reducing the probabilities $P(X = 0, Y \neq 0)$ and $P(X \neq 0, Y = 0)$ by introducing an adjustment factor α as follows:

$$\begin{aligned}
P_\alpha(X|Y=0) &= (1-\alpha)\,P(X|Y=0) \\
P_\alpha(Y|X=0) &= (1-\alpha)\,P(Y|X=0) \\
P_\alpha(X=0,Y=0) &= (1-\alpha)\,P(X=0,Y=0) \\
&\quad + \alpha\,P(X=0 \cup Y=0)
\end{aligned}$$

where $P(X = 0 \cup Y = 0) = P(X = 0) + P(Y = 0) - P(X = 0, Y = 0)$.

Note that theoretically, the adjusted joint probability (i.e. the scoring scheme) could be estimated by ground truth given a large number of registered samples. However, here we treat the adjustment factor as a parameter of the registration pipeline that can be kept constant as $\alpha = .25$. We denote the modified joint probability as $P_\alpha(X, Y)$ as our restricted scoring scheme that incorporates our prior knowledge.

The joint entropy indicating the quality of the matching now becomes

$$\begin{aligned}
H_\alpha(X,Y) &= -\frac{\log \prod_{i=1}^{m} P_\alpha(X = A(i), Y = B(i))}{m} \\
&= -\sum_{x \in \mathscr{X}} \sum_{y \in \mathscr{Y}} p(x,y) \log p_\alpha(x,y),
\end{aligned}$$

and we define

$$I_\alpha(X,Y) = H(X) + H(Y) - H_\alpha(X,Y).$$

We will refer to $I_\alpha(X, Y)$ as the *restricted mutual information*. This measurement serves as a universal metric for measuring nonlinear correspondence with the prior knowledge that certain index pairs should or should not match each other.

Supplement A.2 Sparse Search Optimizer and Initial Radius Estimation

To address the problem that exhaustive search of the complete space of transformations is prohibitively slow in practice, we propose a sparse search optimizer which sparsely searches the space of transformations to obtain the optimal registration position that maximizes the scoring function. The assumptions of this optimizer are that, 1) the solution space is bounded and discrete; and 2), the global maximum is surrounded by a peak of a certain width (Figure S2, illustrated as a minimization rather than maximization problem).

The basic idea of this optimizer is that sudden discontinuities of the function value in the transformation space are hardly observed (which, in fact, is further supported by slightly over-segmenting the two images). In other words, we assume that the target function at one position is at least slightly correlated to the target function values of its neighboring positions. This allows optimizers to focus searching for optima in the surrounding of high scoring candidate positions after sparsely chosen evaluations.

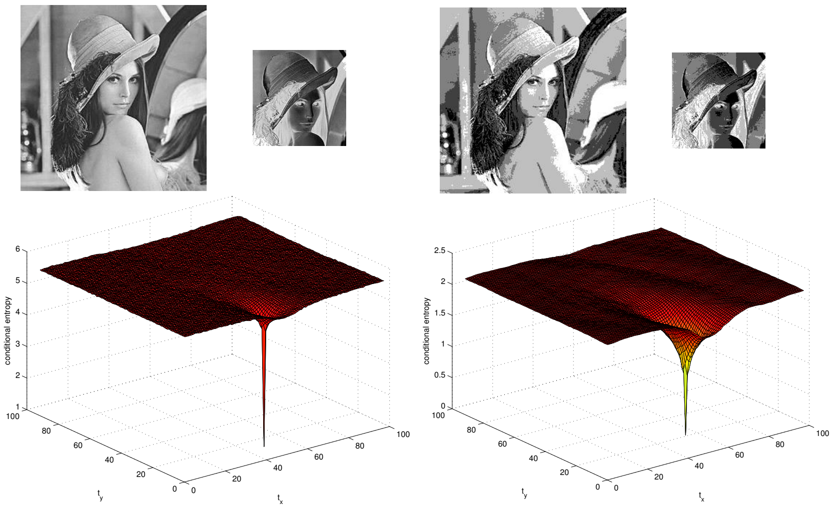

(a) (b)

Supplementary Figure S2. *Width of local optima in solution space.* (a) The conditional entropy map under 2D translations when searching the subimage in the reference image using the gray-scale image (with 256 bins). (b) When using an index image with 5 values, the basin around the true location widens significantly. Note that for illustration purposes, the example presented here considers minima of conditional entropy rather than maxima of (restricted) mutual information.

To describe our sparse search method, we briefly introduce some notation. Suppose we are maximizing the objective function $f(\boldsymbol{\tau})$ from the transformation domain $\boldsymbol{\tau} \in \mathscr{T}$. First, we define an initial radius $\boldsymbol{r}_0$. Based on the value $\boldsymbol{r}_0$, we impose an equidistant grid $E_0 \subseteq \mathscr{T}$ onto the bounded parameter space $\mathscr{T}$ of all transformations

Algorithm: Sparse Search

Initialization:

$\boldsymbol{r} = \boldsymbol{r}_0$
$k = k_0$
$E = E_0$

Iteration:

while any($\boldsymbol{r} > \boldsymbol{r}_{tol}$) **do**
  $v_{min} = \min_{\boldsymbol{\tau} \in E} f(\boldsymbol{\tau})$
  $v_{max} = \max_{\boldsymbol{\tau} \in E} f(\boldsymbol{\tau})$
  $v_{thres} = v_{min} + (v_{max} - v_{min}) * (1 - k)$
  $K = \{\boldsymbol{\tau} | f(\boldsymbol{\tau}) > v_{thres}, \boldsymbol{\tau} \in E\}$
  $E = E \cup (\bigcup_{\boldsymbol{\tau} \in K} N_{\boldsymbol{r}}(\boldsymbol{\tau}))$
  $\boldsymbol{r} = \lceil \boldsymbol{r}/2 \rceil$
  $k = k * \delta k$
end while
$\tau^{\star} = \arg\max_{\boldsymbol{\tau} \in E} f(\boldsymbol{\tau})$

Supplementary Figure S3. Sparse search algorithm.

as a "sparsely" chosen subset. Next, we define two parameters k_0 and δk as the *keeping factors* which determine the threshold for keeping transformations that we will search around. Parameter k_0 is the initial keeping factor, while δk is the decrementation factor of the keeping factor at each iteration. Furthermore, we define the *neighborhood* $N_{\boldsymbol{r}}(\boldsymbol{\tau})$ of a transformation $\boldsymbol{\tau}$ as the hypercube centered at $\boldsymbol{\tau}$ with the side lengths of $2\boldsymbol{r}$.

During each iteration of the sparse search, we reduce the subset $K \subseteq \mathscr{T}$ of transformation whose neighborhoods with radius $\boldsymbol{r}$ remain to be investigated. Figure S3 shows the pseudo-code of the resulting sparse search algorithm. Furthermore, Figure 3 shows an example of the sparse search in a 2D translation only scenario with equal radius in x and y direction.

The sparse search and image pyramid representation is used to form a coarse- to-fine optimization. At fine-level registration steps, the initial points E_0 are directly provided by some promising candidates of coarse registration.

The sparse search procedure can been seen as a generalization of the image pyramid based on the fact that sometimes, the search space can be further "sub-sampled" whereas the image cannot be further subsampled due to the lack of information for metric. With sparse search, we are using the image signal on a lower level while searching the transformation space on a higher level.

The sparse search optimizer represents a compromise between heuristics and exhaustive search which highly increase the chance of finding out the correct transformation with a significant improvement of time efficiency. It is conceptually related to heuristic algorithms in path finding problems. In the terminology of genetic algorithms, E_0 can also be viewed as an initial population. Yet, our sparse search procedure remains a heuristic, as it lacks provable bounds in the spirit of a branch-and-bound algorithm. When the radius $\boldsymbol{r}_0$ is small, results approach those of exhaustive search.

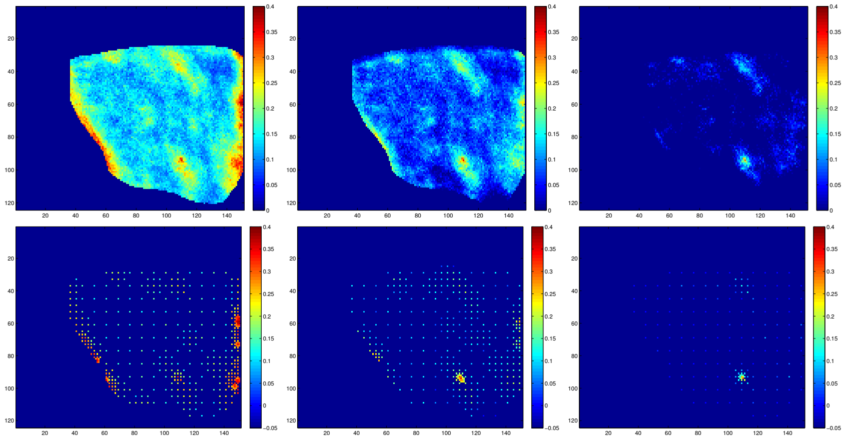

(a) (b) (c)

Supplementary Figure S4. *Restricted mutual information score maps under translations in 2D*. (a) complete map and sparse search evaluations of the function space using mutual information as metric; (b) complete map using sparse search evaluations of the function space using restricted mutual information with adjustment factor $\alpha = 0.25$; (c) same for adjustment factor $\alpha = 0.5$.

A.2.1 Transformation Space Discretization

In order to make the solution space discrete for the sparse search optimizer, we have to discretize the dimension of the rotation and scaling parameter. We define the *elementary unit* as the smallest unit of the solution space. Clearly, the elementary unit should be a small transformation that can change the point correspondence of the overlay of two images.

The elementary unit of translation is clearly 1 pixel. For the elementary unit of rotation and scaling, suppose we have point p with the coordinate (x, y). The minimum rotation needed to change the coordinate of p to another grid, can be detected using

$$\Delta\theta = \arg \max_{\delta\theta \in [0, 2\pi]} \quad \{ \\ x - 1 \leq r\cos(\theta + \delta\theta) \leq x + 1 \\ y - 1 \leq r\sin(\theta + \delta\theta) \leq y + 1 \\ \}$$

The minimum scaling needed obviously can be obtained by

$$\Delta s = \frac{1}{\min(x, y)},$$

and we define the elementary unit of rotation and scaling as the $\Delta\theta$ and Δs of the center of the fixed image.

A.2.2 Initial Radius Estimation

An essential step for the sparse search optimizer is to determine the initial radius $\boldsymbol{r}_0$. If the initial radius is too large, no transformation "inside the peak" of the optimal transformation will be contained in E_0, so that the global optimum will be missed. Contrarily, if the radius is too small, there will be no significant speed up compared to exhaustive search.

Intuitively, the proper initial radius depends on the width of the peak surrounding the maximum in the solution space. While it is impossible to know the width of this peak before registration, we introduce an approach to approximate a suitable radius *from the fixed image only*, i.e. by only considering the presegmentation of the infrared image, and *not* involving the stained image at all.

To motivate our approach, consider the extreme case that the two images to be registered are perfectly correlated, i.e. $I(X, Y) = H(X) = H(Y)$. Then the peak width around the correct registration depends on the structure of the image. Intuitively, images involving high frequency components will require a smaller initial radius, whereas images exhibiting larger "low frequency" structures allow choosing a larger initial radius.

The idea proposed here is to estimate how much information a pixel can tell about its neighborhood. To this end, we can estimate the radius through a *self registration* of the target image, assuming it is representative of the registration around the true transformation. In order to increase the chance of finding the global optimum, we approximate a lower bound of the initial radius.

As a first simplifying assumption, we consider the parameters in transformation space (i.e., translation in each direction, rotation, and scaling) mutually independent from each other, so that they can be estimated separately. To motivate our approach to estimate $\boldsymbol{r}_0$, we consider the worst case scenario illustrated in Figure S5a in a single parameter setting. In the first iteration, a false optimum was evaluated while the transformations inside the peak are symmetrically located such that the objective function grows monotonically towards the peak. In this case, we must make sure that the transformations around the true optimum are kept for further consideration. To guarantee this, a bigger keeping factor or a smaller initial radius needs to be chosen. Suppose we are using an initial keeping factor slightly higher than 0.5, then the Full Width at Half Maximum (FWHM) will be a reasonable estimation of the initial radius.

Since the peak around the perfect match in a self registration is usually steeper than the peak observed in the registration between two images, we can reasonably assume that false local optima are no better than the value around the true position to get a relatively bigger estimation. Thus, we take the Full Width at Half Maximum (FWHM) of the peak (without the sharp peak) in the self registration as the estimation of the initial $\boldsymbol{r}_0$. As shown in Supplementary Figure S5b, this is actually a very conservative estimation that hugely increase the chance of finding the right registration, and the optimizer could usually work on a much bigger initial radius in practice.

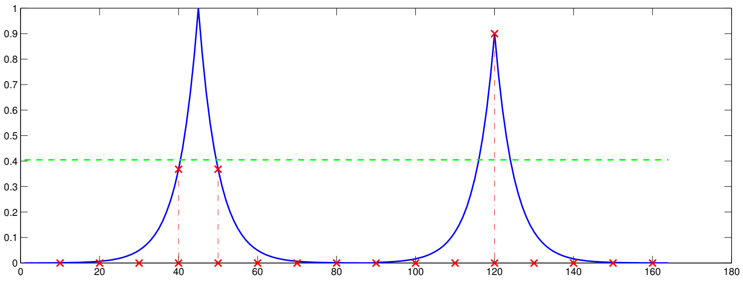

(a)

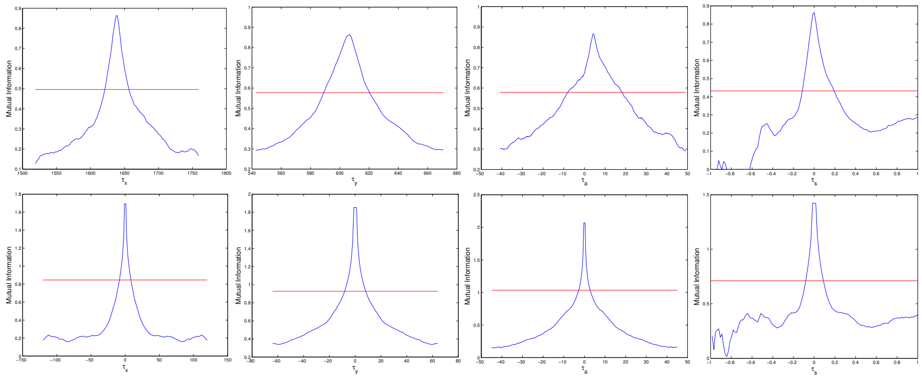

(b)

Supplementary Figure S5. *Initial radius estimation.* (a) The worst case occurs in the first round of optimization. The mark ($\times$) indicates the position evaluated during first round, the horizontal line indicates the threshold computed for keeping points to search in the next round. (b) The function value around the true location through registration (upper row) and self registration (bottom row) of translation in x and y direction, respectively, rotation, and scaling. The horizontal line indicates the FWHM.

A.2.3 Evaluation Metrics

In order to obtain an objective measure of comparability between different registration positions (without involving the scoring function used for registration), we introduce the *regression error* as a measure of accuracy as follows. For a given transformation, a multiple linear regression model can be used for learning the relationship between the high-dimensional spectra in the spectral image and the RGB colors in the stained image. We assume that for the correct transformation, the correlation between the spectra and the colors achieve an (either local or global) maximum. Consequently, the regression error should be minimized. Therefore, the regression error is used for measuring the registration accuracy, expressed as the mean distance of the predicted colors by the spectra and the real colors in the stained image in a $[0, 255]$ RGB color space. Additionally, we compare the difference of transformations by the mean distance of the mapped coordinates of predefined points through different transformations in the image coordinate space. We refer to the two metrics as RMDE (Regression mean distance er-

ror) and TMDE (Transformation mean distance error), respectively, in Supplementary Table F.2.

Supplement A.3 Virtual Staining based Registration

A.3.1 Methods

While the *RMI/sparse* approach discussed in the manuscript is based on comparing discrete presegmentations, it occurs natural to employ more immediate correlations between the pixel spectra and the staining color space in the H&E image. A straightforward way to achieve this is to perform *virtual staining* (or "stainless staining") [3], i.e., predict the pixel color in H&E color space from the corresponding pixel spectrum. This can be achieved quite simply by a regression classifier for the red, the green, and the blue channel of the virtually stained image to be predicted [3]. The general appearance of the image is visually well preserved, as shown in Supplementary Fig. S6.

The RGB image thus obtained is in principle of same (or at least comparable) modality, so that registration can be performed using conventional registration approaches. We validated two approaches for registration.

- *VS/xcorr*: We used an area-based approach using cross correlation as a metric. More precisely, we used the Pearson-correlation metric implemented in the `normxcorr2` function of the *Matlab* image processing toolbox. As we relied on implementations of the *Matlab* image processing toolbox, technical reasons prohibit combining this approach with the sparse search optimizer. Thus, for efficiency reasons, some validations could be performed under translations only rather than under rigid motions.
- *VS/feature*: We extracted SIFT features form the H&E stained and used RANSAC for determinig candidate transformations, and evaluated *absolute different area* to determine the best transformation.

A.3.2 Results

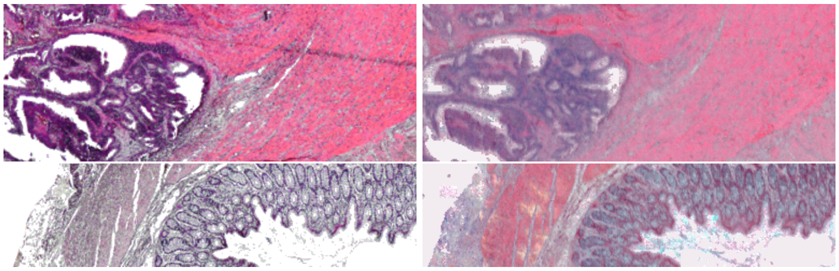

(a) (b)

Supplementary Figure S6. *Virtual staining of the spectral image by a regresison model learned from other spectral images from the same sample.* (a) Cutouts of stained images; (b) virtually stained images

		RMI/sparse		*Binary/xcorr*		*VS/feature*		*VS/xcorr**	
Sample	Type	# Correct	Time	# Correct	Time	# Correct	Time	# Correct	Time
S7	lung	83/100	146.21	32/100	513.17	22/100	91.96	42/100*	*n.a.**
S8	colon	62/100	136.53	35/100	465.78	0/100	91.88	22/100*	*n.a.**
S9	colon	60/100	134.291	25/100	418.85	5/100	93.53	38/100*	*n.a.**

* *VS/xcorr evaluated under translation only.*

Supplementary Table S1. Comparison of results and running time of different approaches on random cut outs of three samples. The setting assessed corresponds to the scenario in Figure 4 in the main manuscript. For technical and running time reasons, *VS/xcorr* could only be evaluated under translation. Accordingly, the number of cut outs registered correctly by *VS/xcorr* is relatively high, and can be expected to drop significantly if rotations would be allowed, as suggested by the results under full rigid motions on Sample S4 (Figure S7). Overall, our proposed *RMI/sparse* is clearly the most reliable approach across all data sets.

.

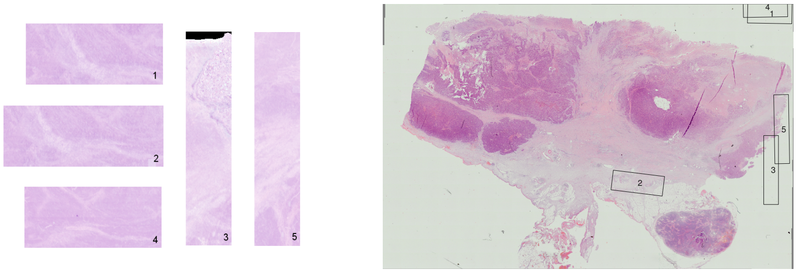

(a) (b)

Supplementary Figure S7. *Registration of the Sample S4 by virtual staining (regression model trained on sample S3) and cross correlation.* (a) Virtually stained spectra images; (b) registration results. The registered position usually falls in the background region because no highly correlated region can be found.

References

[1] Thomas M Cover and Joy A Thomas. *Elements of Information Theory 2nd Edition (Wiley Series in Telecommunications and Signal Processing)*. Wiley-Interscience, 2006.

[2] David Mattes, David R Haynor, Hubert Vesselle, Thomas K Lewellen, and William Eubank. PET-CT image registration in the chest using free-form deformations. *IEEE transactions on medical imaging*, 22(1):120–8, January 2003.

[3] David Mayerich, Michael J Walsh, Andre Kadjacsy-Balla, Partha S Ray, Stephen M Hewitt, and Rohit Bhargava. Stain-less staining for computed histopathology. *Technology*, 3(01):27–31, 2015.

[4] Josien P W Pluim, J B Antoine Maintz, and Max a Viergever. Mutual-information-based registration of medical images: a survey. *IEEE transactions on medical imaging*, 22(8):986–1004, August 2003.

[5] P Thévenaz and M Unser. Optimization of mutual information for multiresolution image registration. *IEEE transactions on image processing : a publication of the IEEE Signal Processing Society*, 9(12):2083–99, January 2000.

[6] P Viola and W.M. Wells. Alignment by maximization of mutual information. In *Proceedings of IEEE International Conference on Computer Vision*, number 1548, pages 16–23. IEEE Comput. Soc. Press, 1997.
