# Supplementary material for: Fully automated registration of vibrational microspectroscopic images in histologically stained tissue sections
Source: BMC Bioinformatics. 2015 Nov 25;16:396. doi: 10.1186/s12859-015-0804-9 (PMC4659215; doi:10.1186/s12859-015-0804-9)
Supplement: Additional file 3 — Tissue Microarray Registration Results. Detailed registration results of 56 FTIR vs. H&E TMA cores. (PDF 46387.2 kb) [file 12859_2015_804_MOESM3_ESM.pdf]

Fully Automated Registration of Vibrational Microscopectroscopic Images in Histologically Stained Tissue Sections

Additional File 3

Figure Legend

		MS/gradient	*MS/evo*	*Binary RMI/sparse*	*RMI/sparse*
H&E stained image	Fore-background segmentation of H&E image.	Overlay of binary images after registration using mean squares metric and gradient descent optimizer.	Overlay of binary images after registration using mean squares metric and evolutionary optimizer.	Overlay of binary images after registration using our pipeline with fore-background segmentation (2 indexes) only.	Overlay of binary images after registration using our pipeline with 10 clusters pre-segmentation.
sum of the spectral image	Fore-background segmentation of spectral image.	registered H&E image using mean squares metric and gradient descent optimizer.	registered H&E image using mean squares metric and evolutionary optimizer.	registered H&E image using our pipeline with fore-background segmentation (2 indexes) only.	registered H&E image using our pipeline with 10 clusters pre-segmentation.

Overlays in top row of columns 3–5 were produced using the Matlab function *imshowpair*. Purple indicates foreground in the registration transformed H&E image, green foreground in the FTIR image, and white areas indicate matching foreground areas in both modalities. (Note that the H&E image takes the role of the moving image.) Correct results can be seen from overlapping foreground (white) and background (black). In the top row of column 6, color coding is transformed from index colors to intensities following the *impshowpair* function. Correct results can be seen from overlapping background area (black).

Among the 56 tissue microarray spots under consideration, the *MS/evo* produced 15 wrong registrations (spots A3, B6, C1, C10, E6, F7, G5, G6, G7, G8, I2, I7, J3, J4, J5), the *MS/evo* 3 wrong results (spots A3, G6, G8), while both *Binary RMI/sparse* and *RMI/sparse* produced one wrong result (both on spot G8).

Correctness of each registration result was determined visually and is indicated by the **correct** or **wrong** labels in columns 3–6.

Acronyms of the different registration approaches are used as introduced in the paper, namely *RMI/sparse* (restricted mutual information between clustering-based presegmentations in combination with our newly proposed sparse search optimizer); *Binary RMI/sparse* (restricted mutual information in combination with binary foregound/background segmentations and the sparse search optimizer); *Binary MS/gradient* (mean-squares metric on binary foreground-background segmentations in combination with a gradient optimizer); *Binary MS/evo* (registrations under the binary sum-of-squares metric in combination with an evolutionary optimizer).

Spot A2

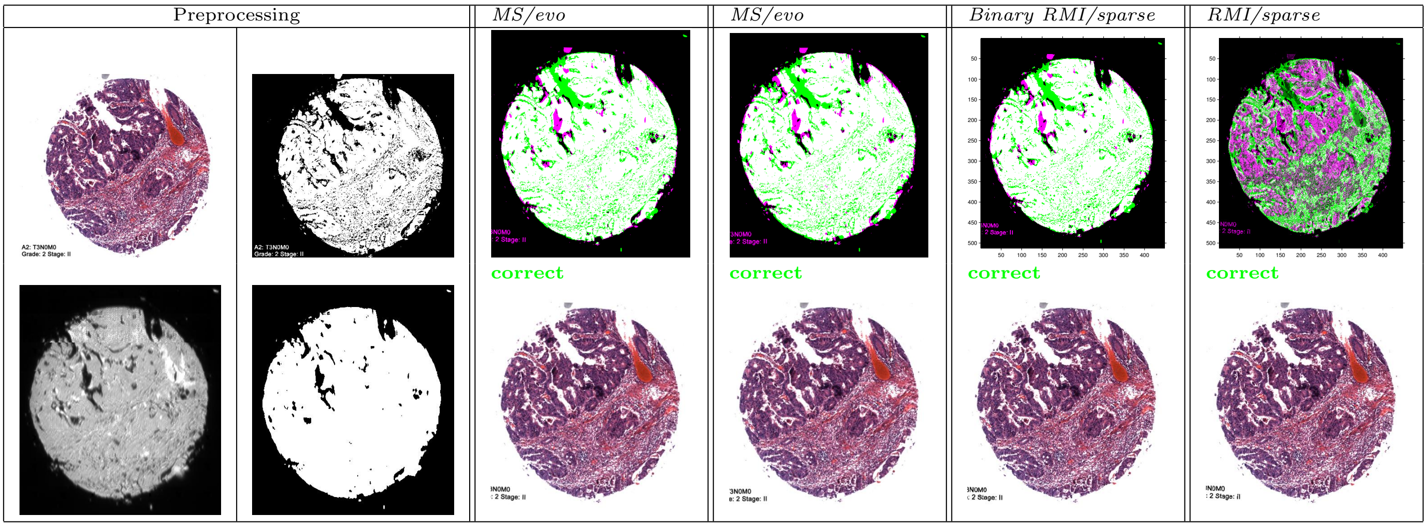

Spot A3

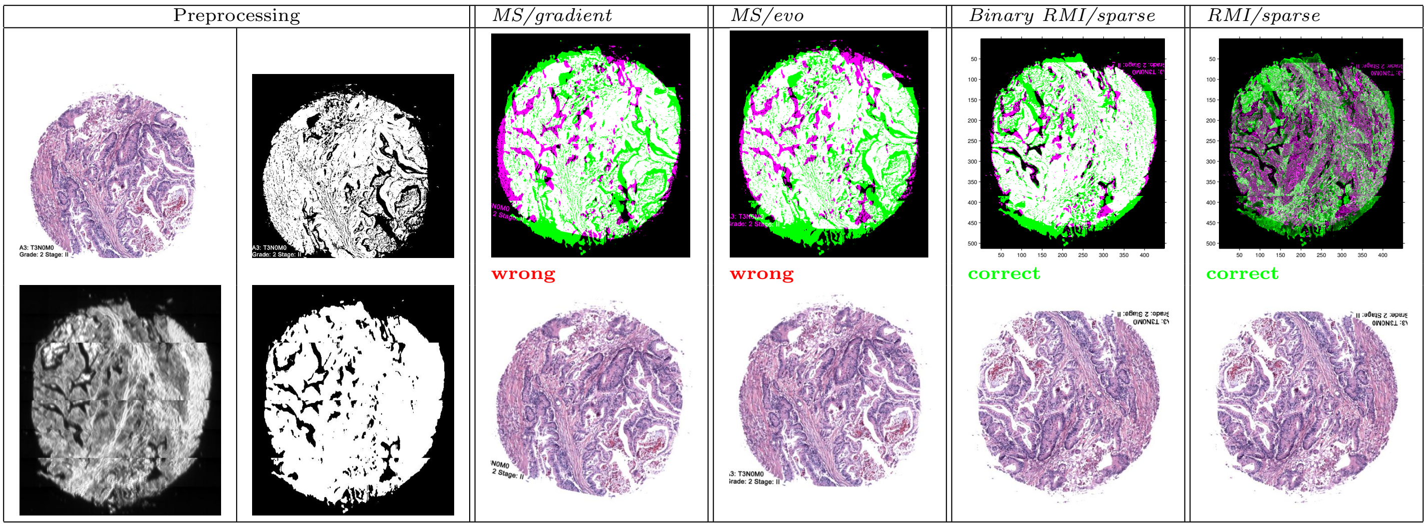

Spot A4

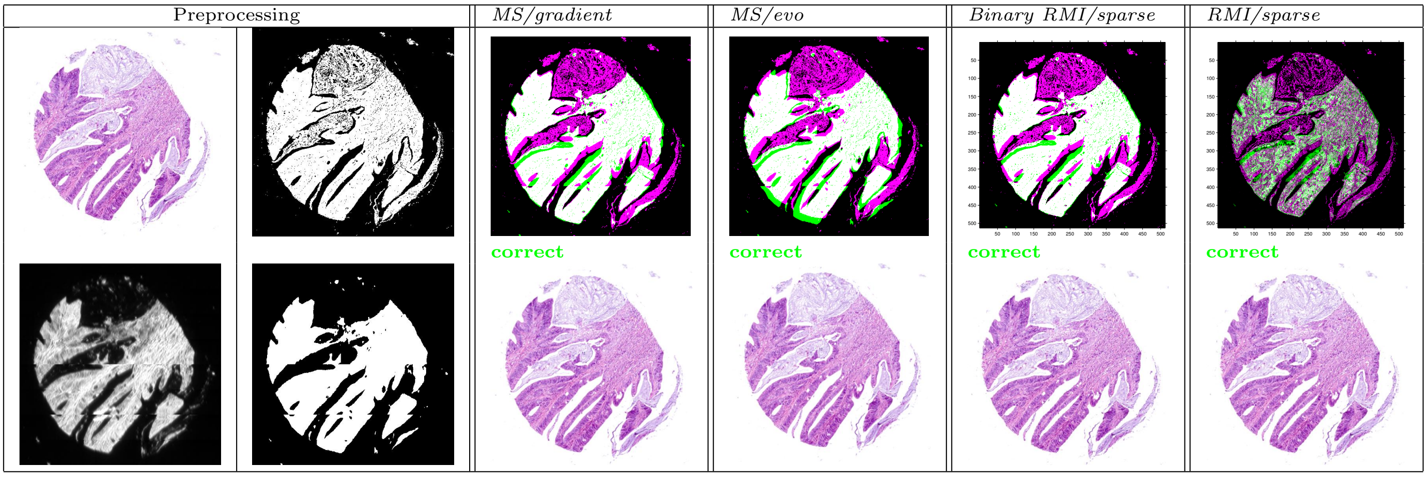

Spot A5

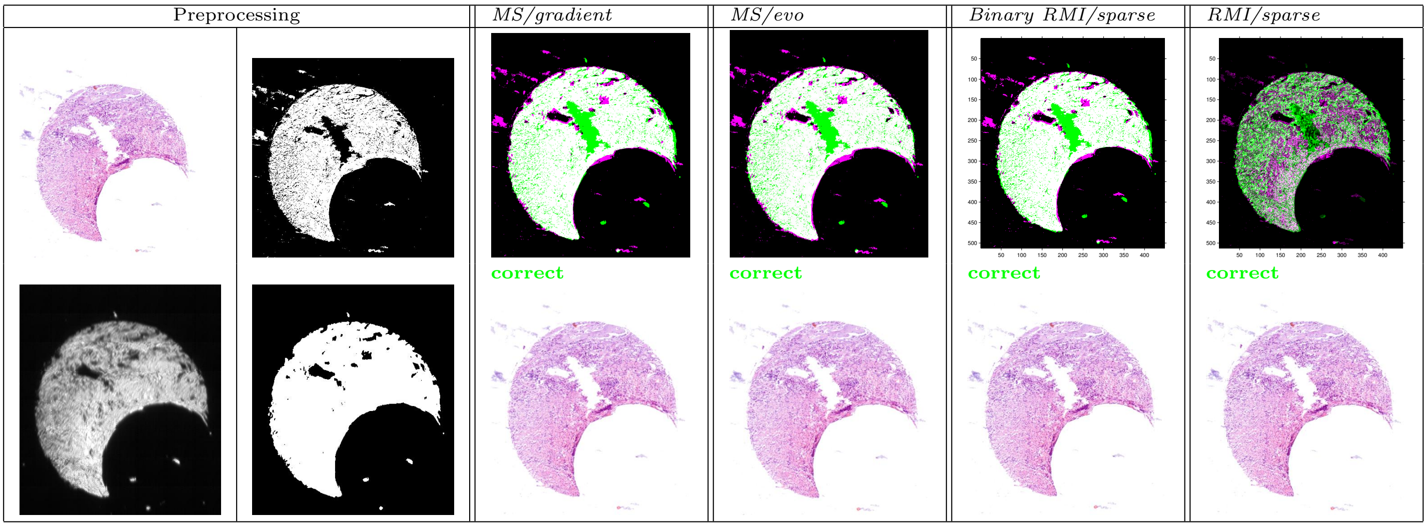

Spot A6

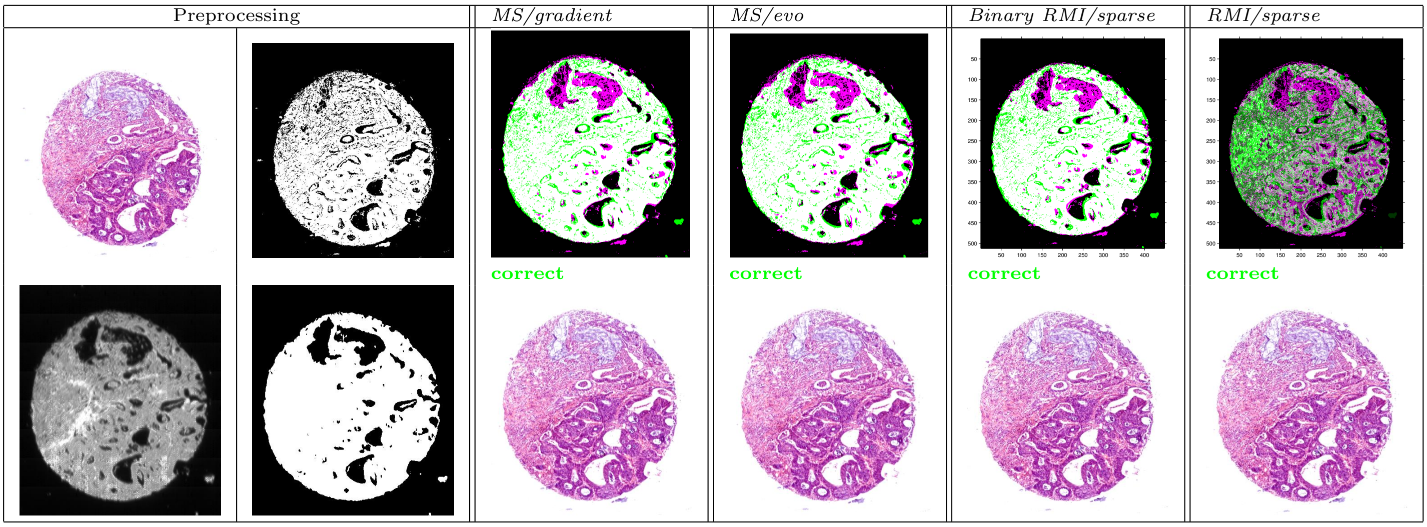

Spot A7

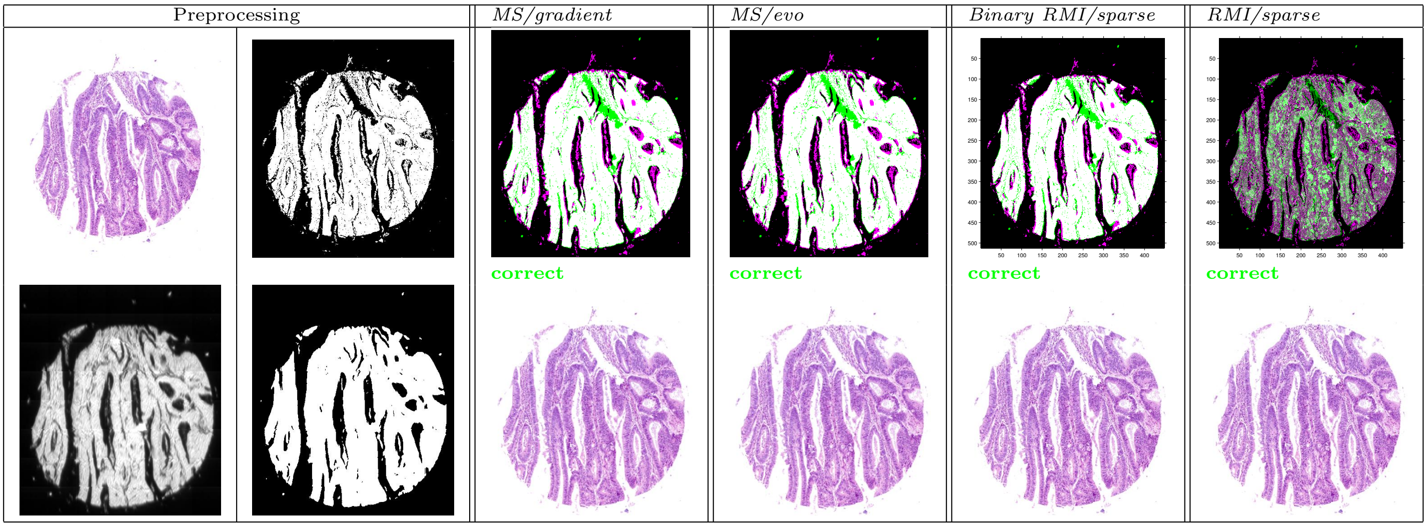

Spot A8

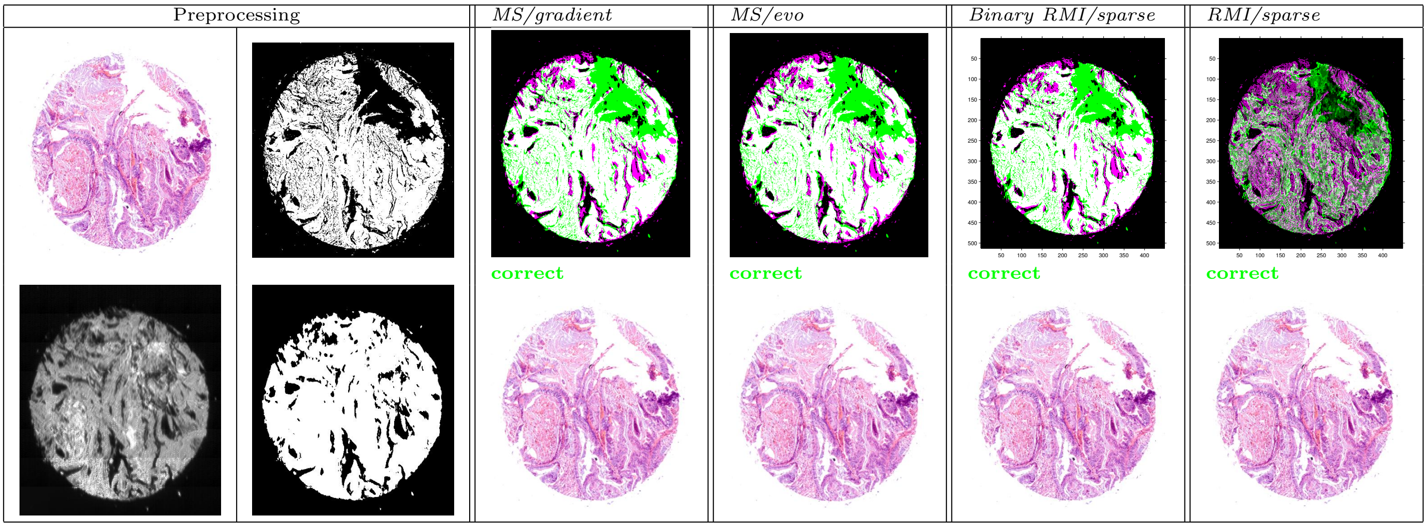

Spot A9

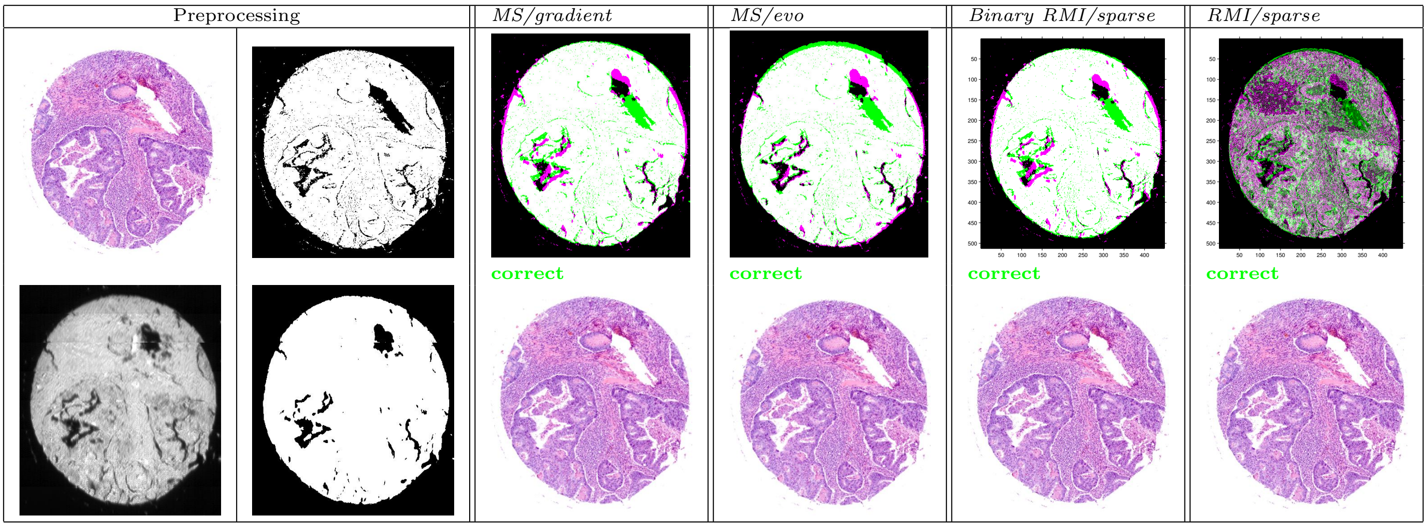

Spot A10

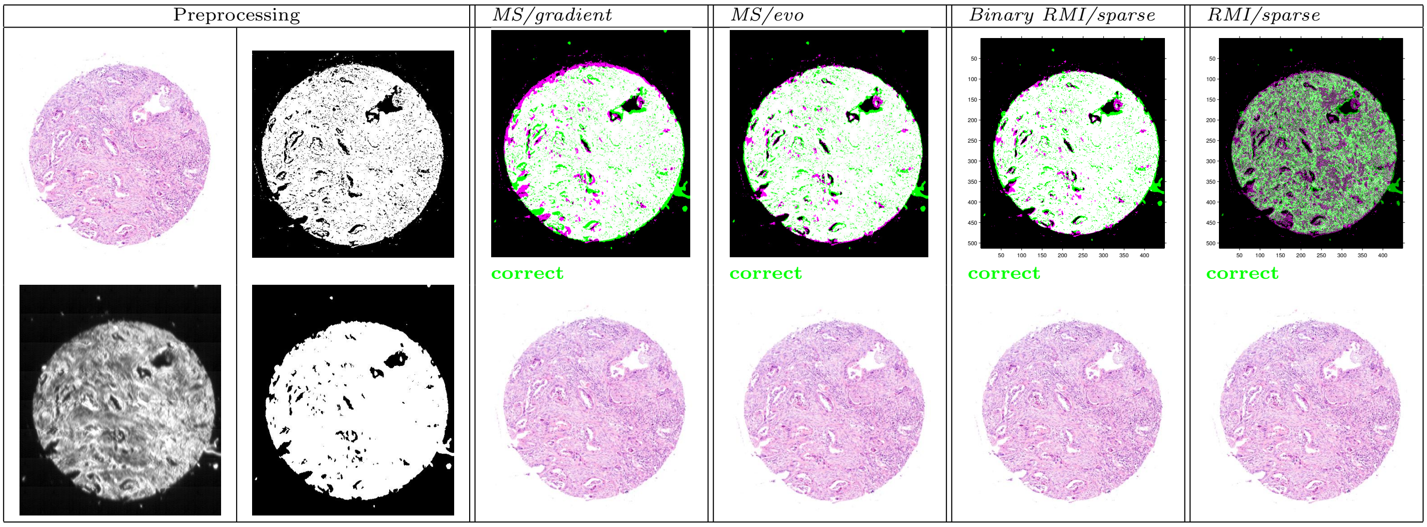

Spot B1

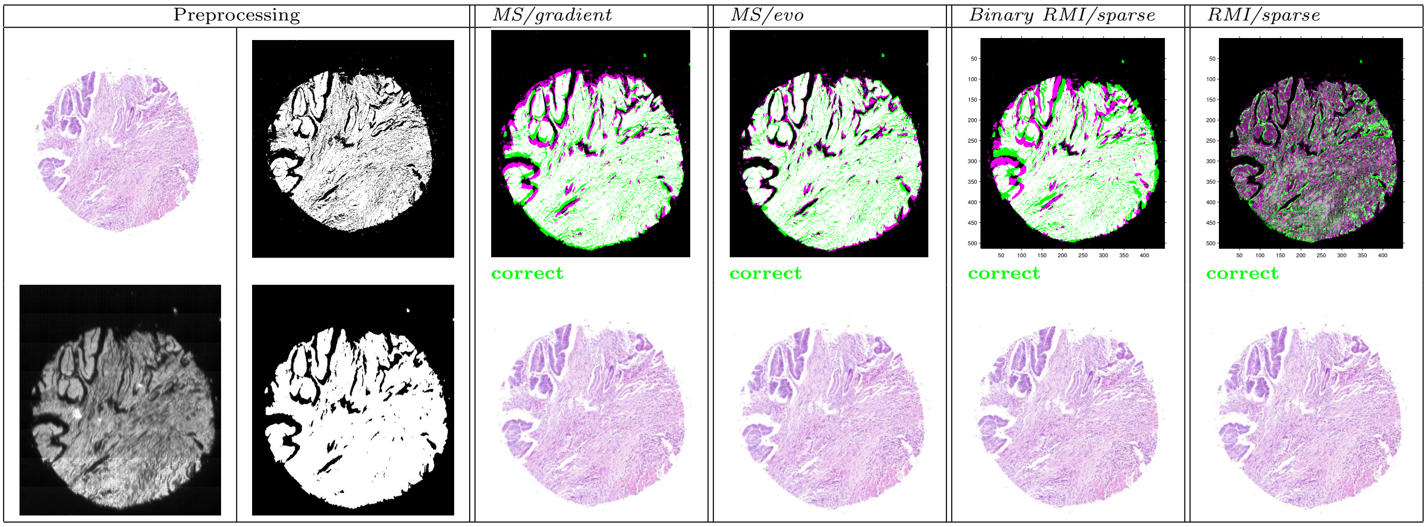

Spot B2

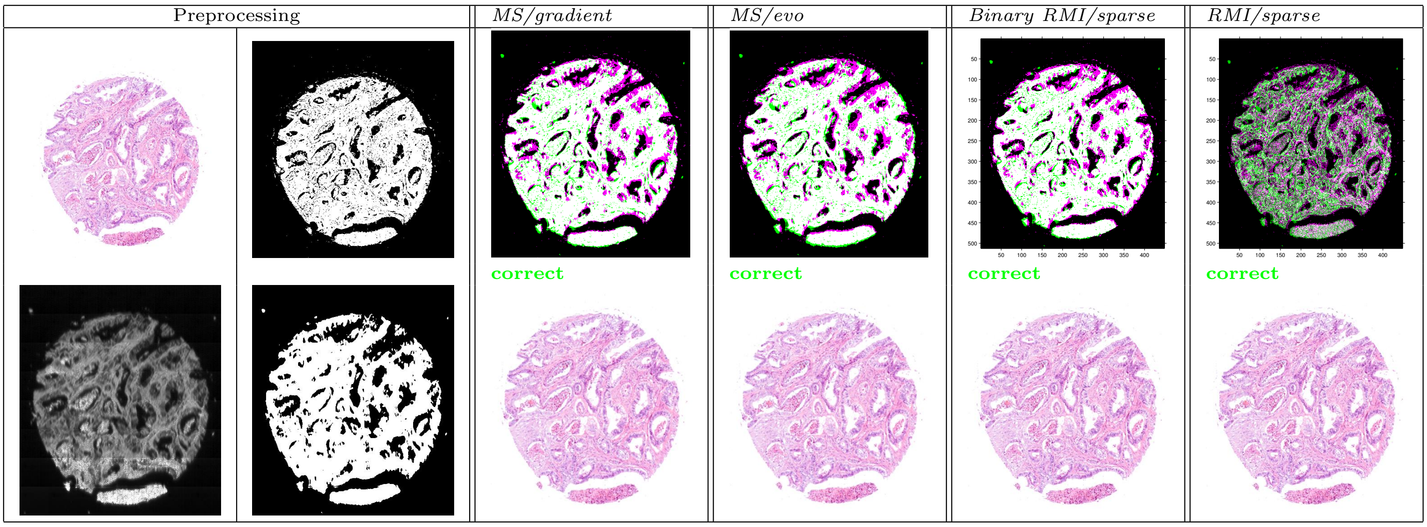

Spot B3

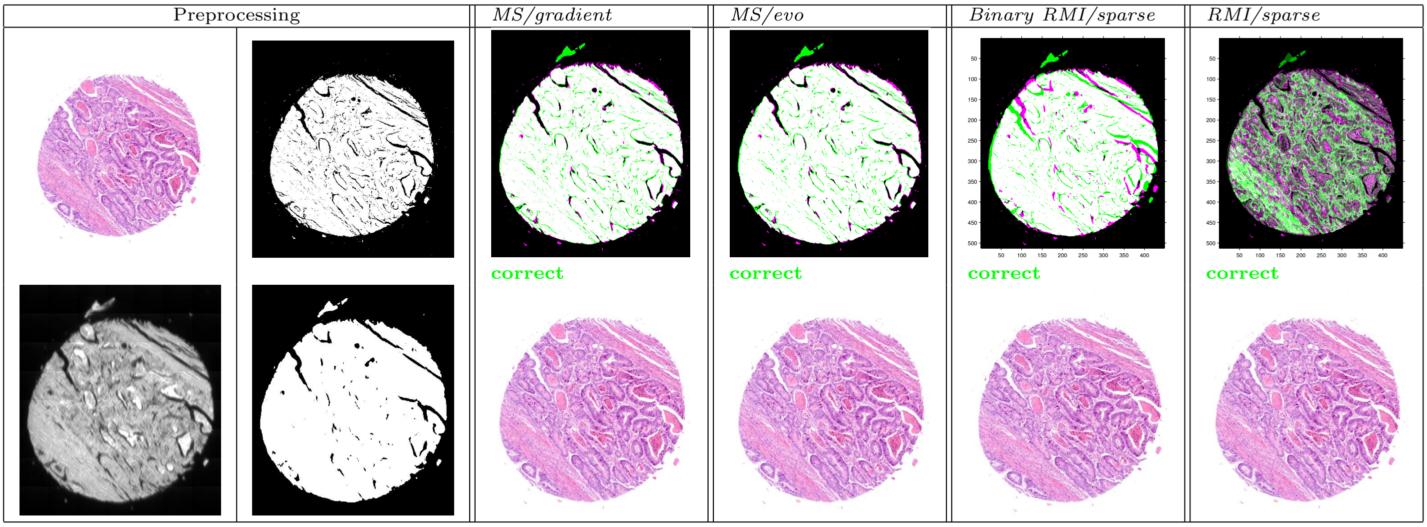

Spot B4

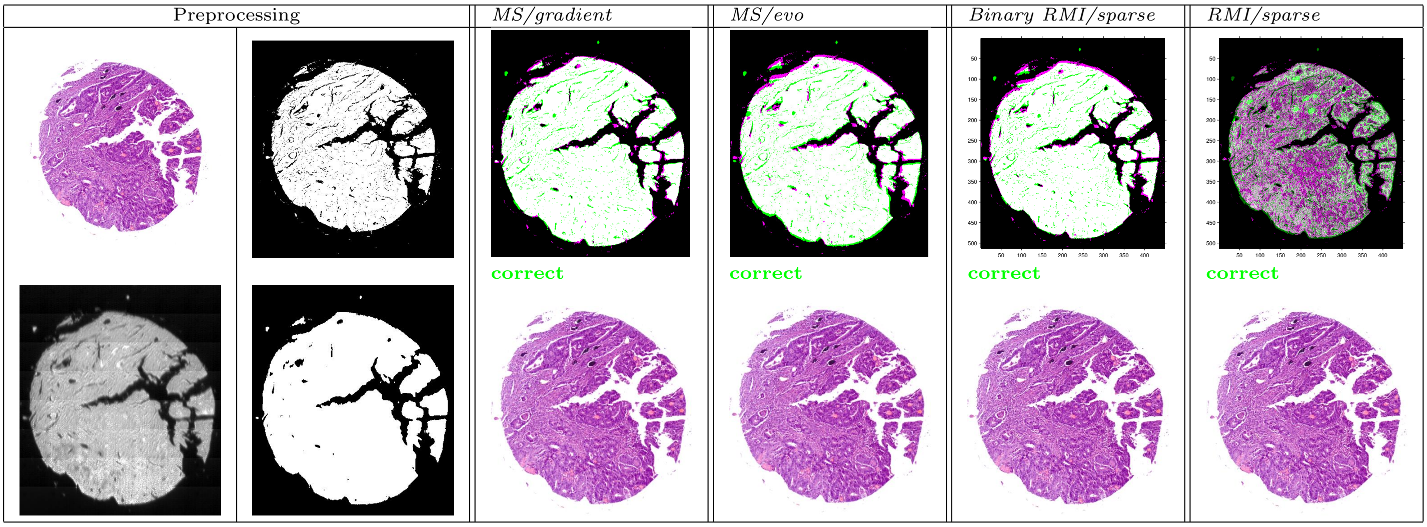

Spot B5

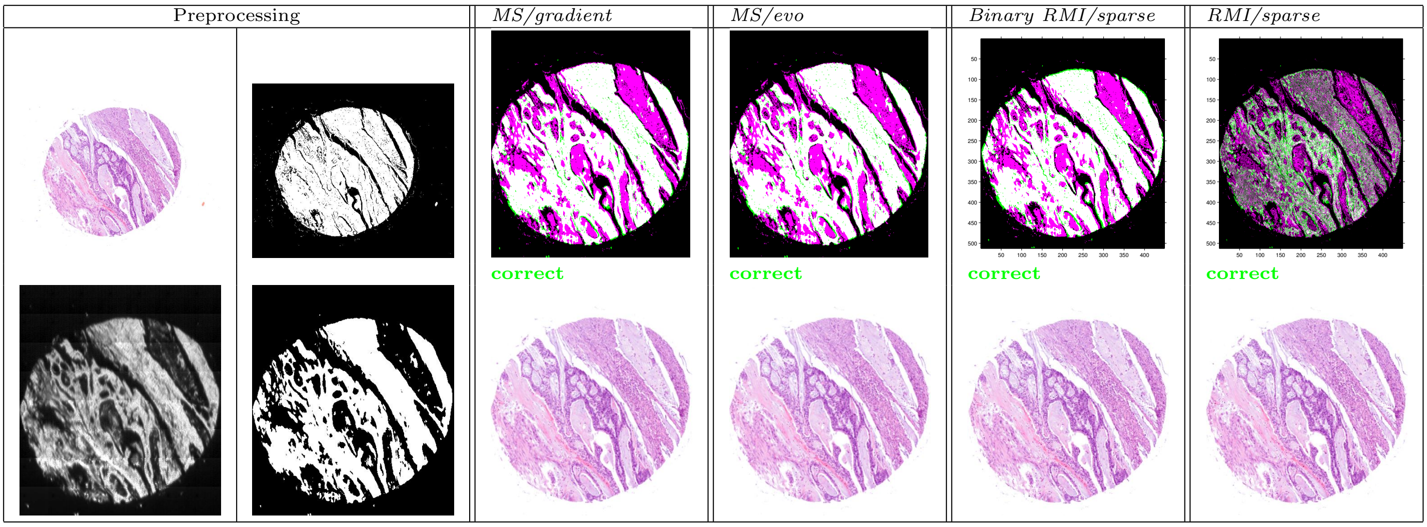

Spot B6

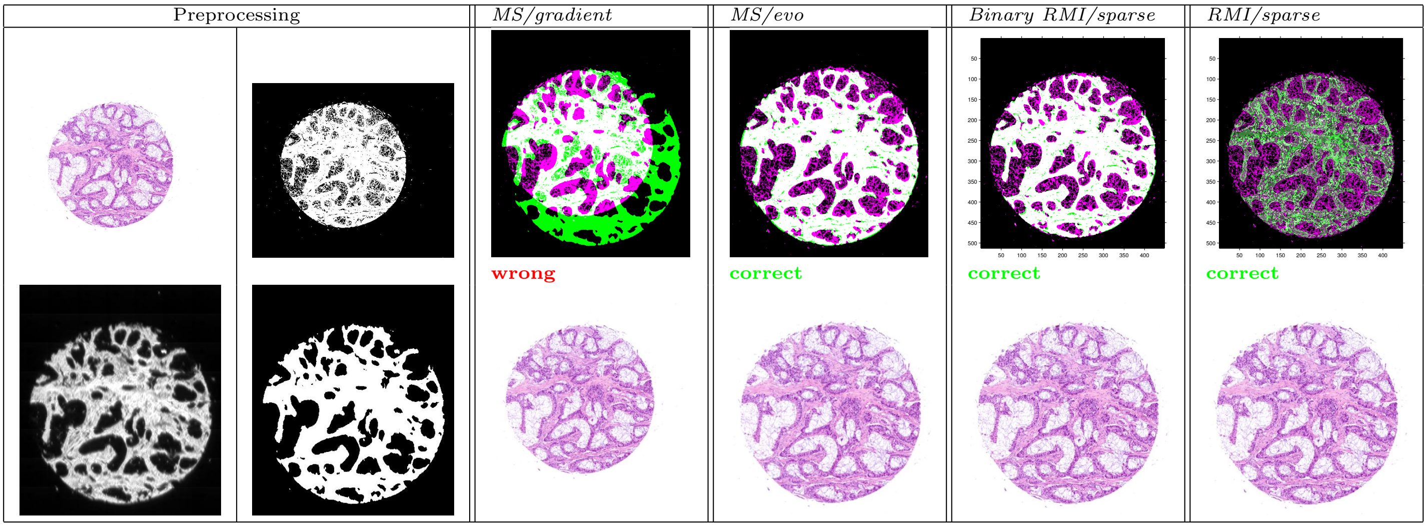

Spot B7

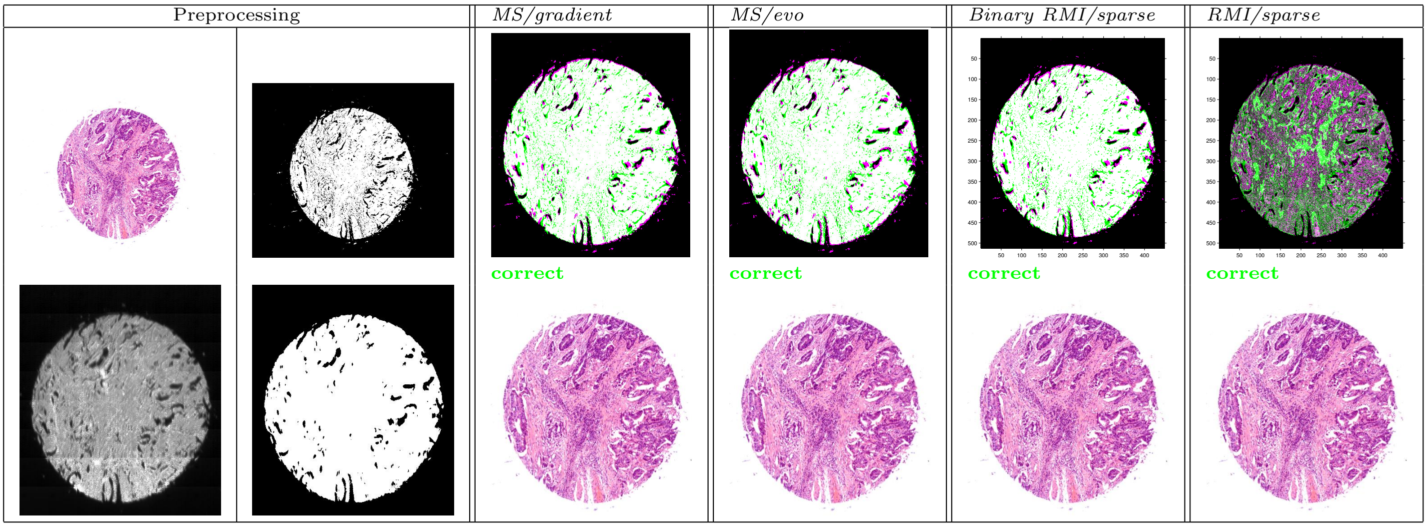

Spot B8

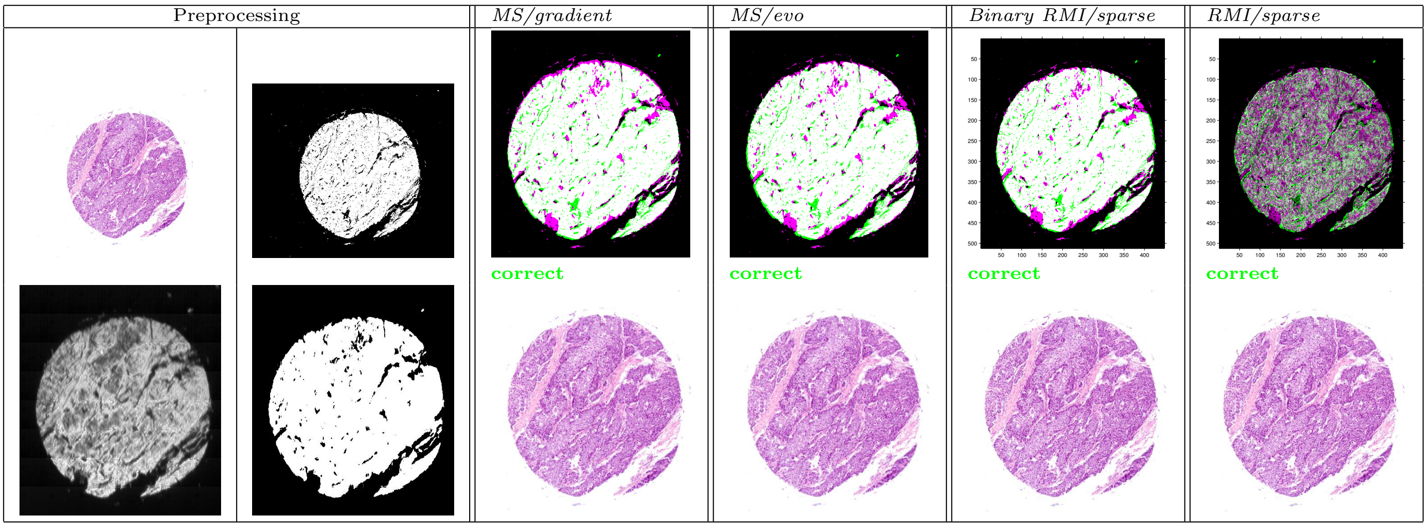

Spot B9

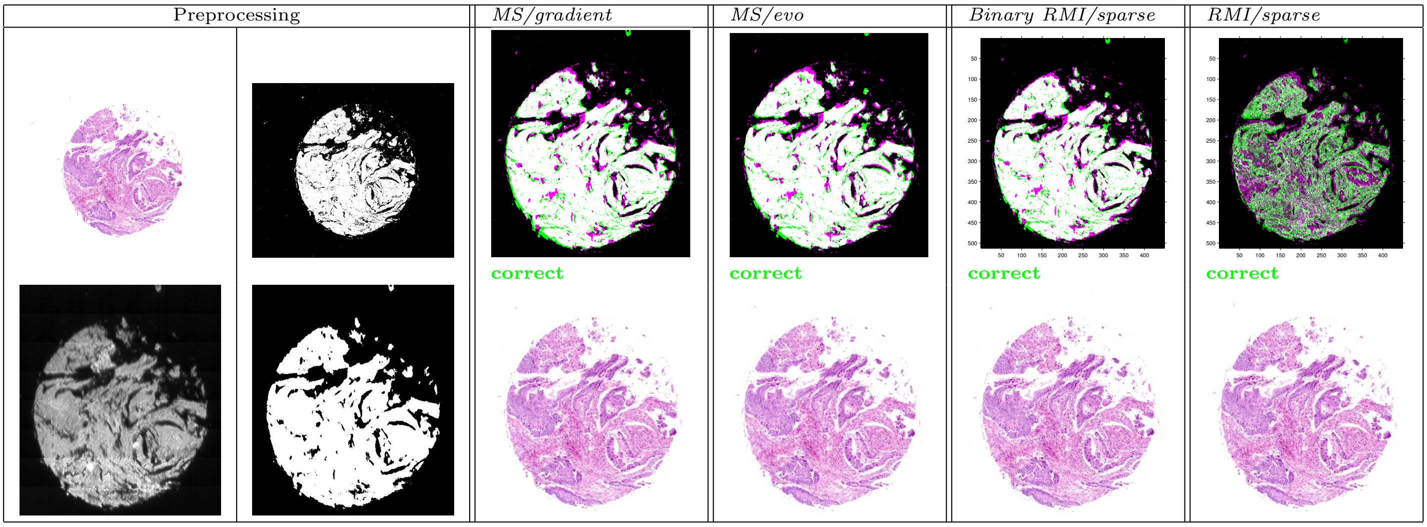

Spot B10

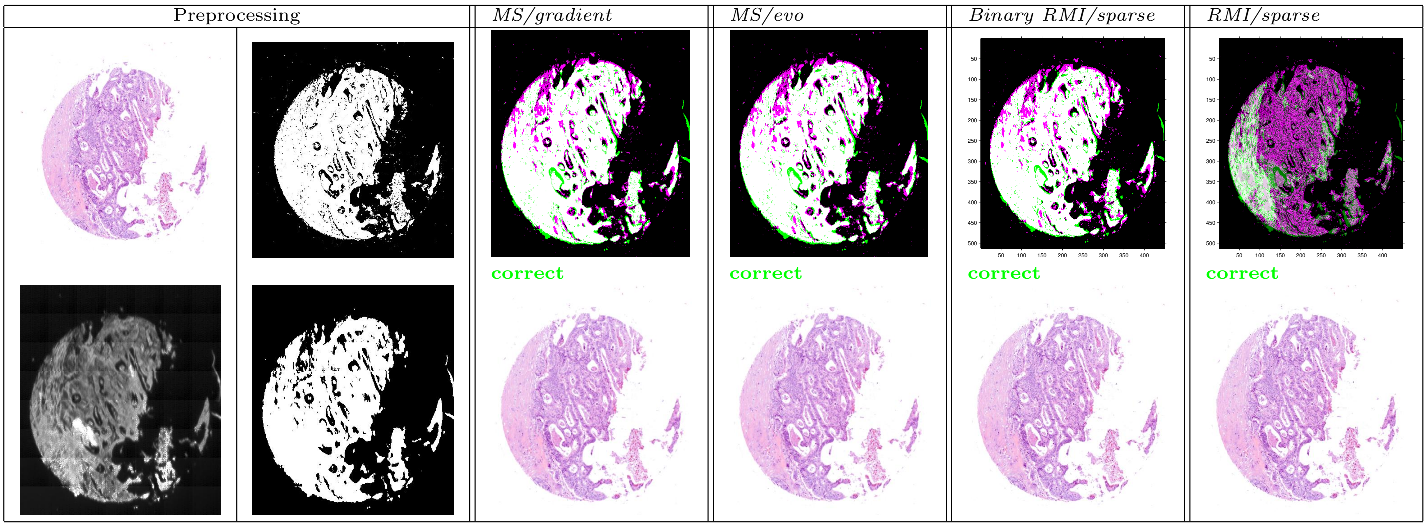

Spot C1

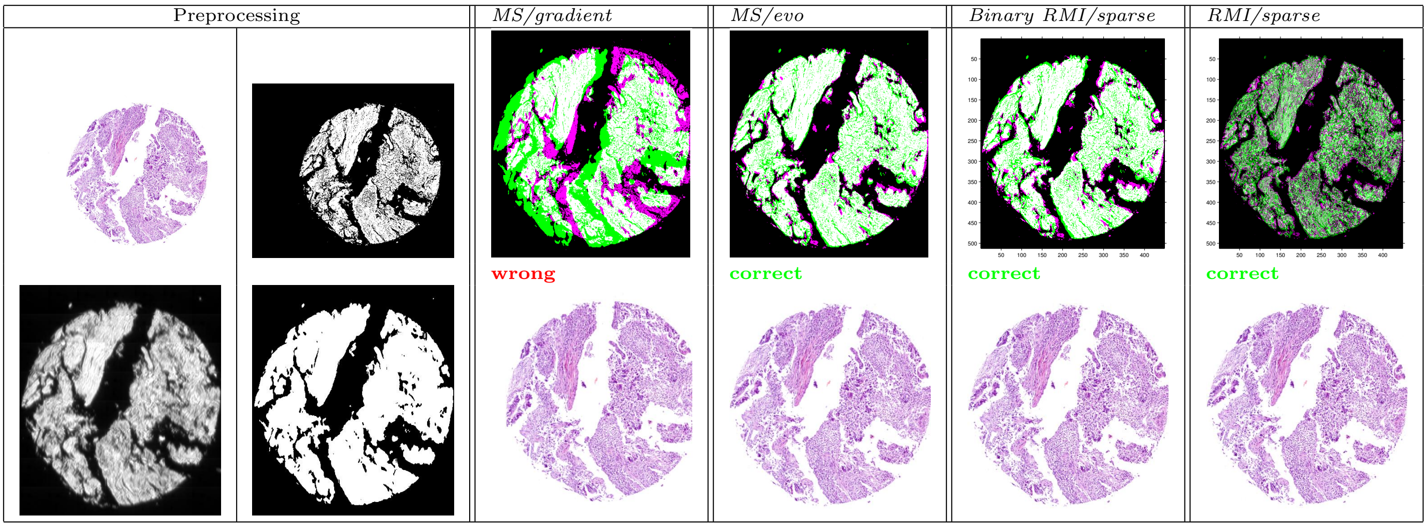

Spot C2

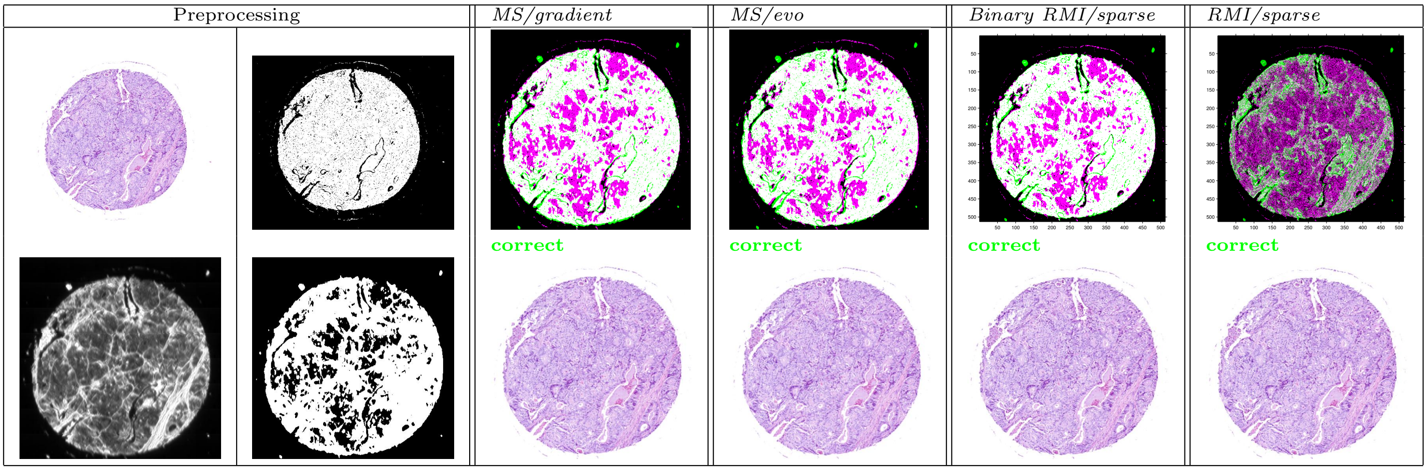

Spot C4

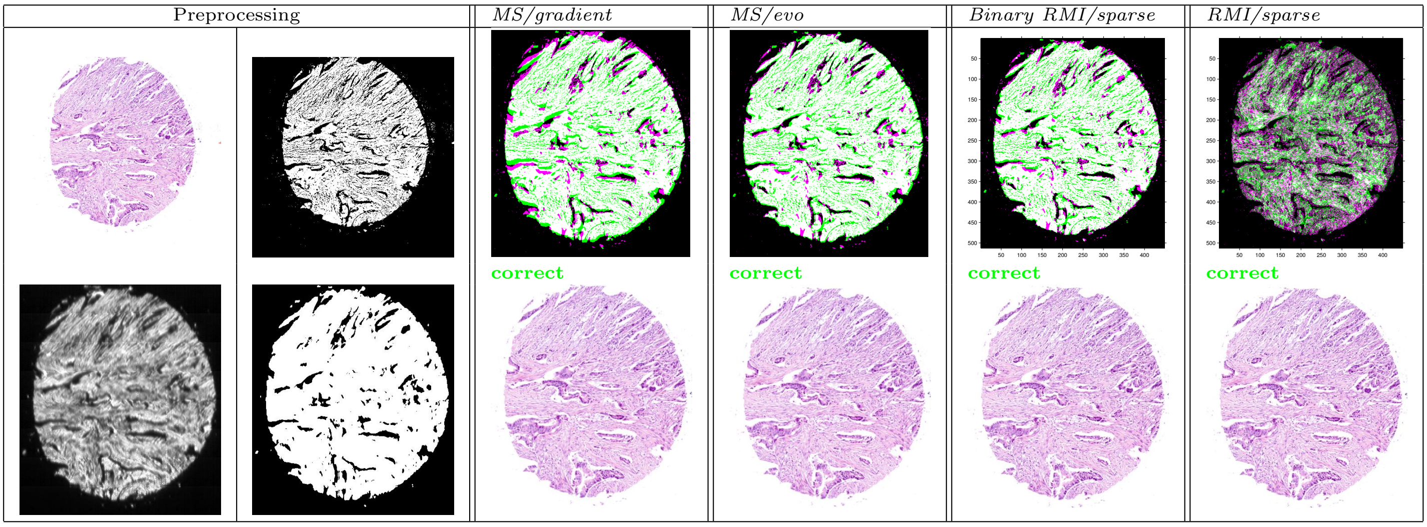

Spot C5

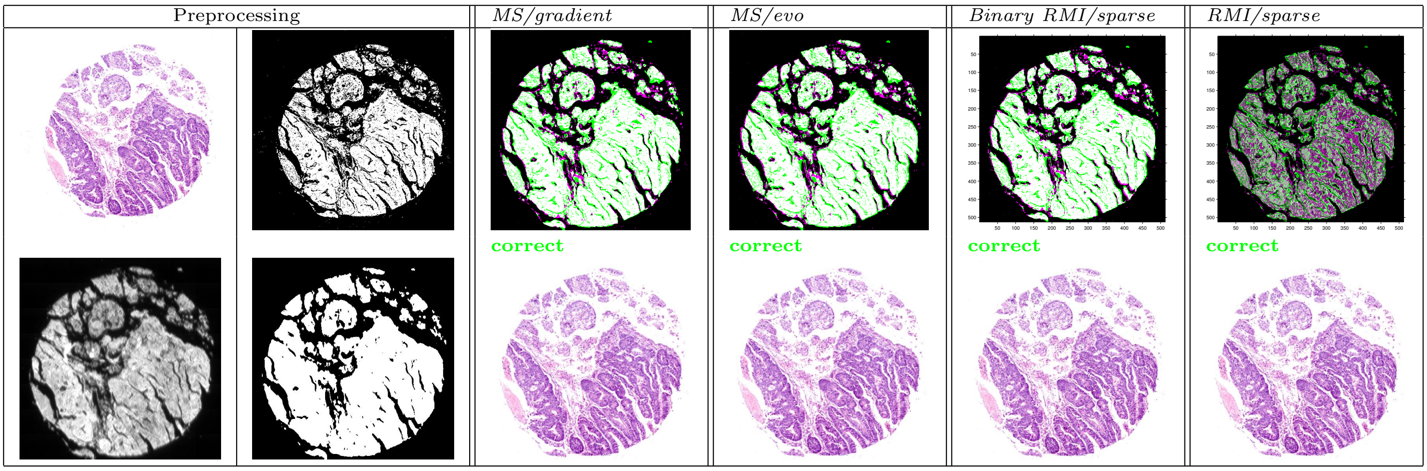

Spot C6

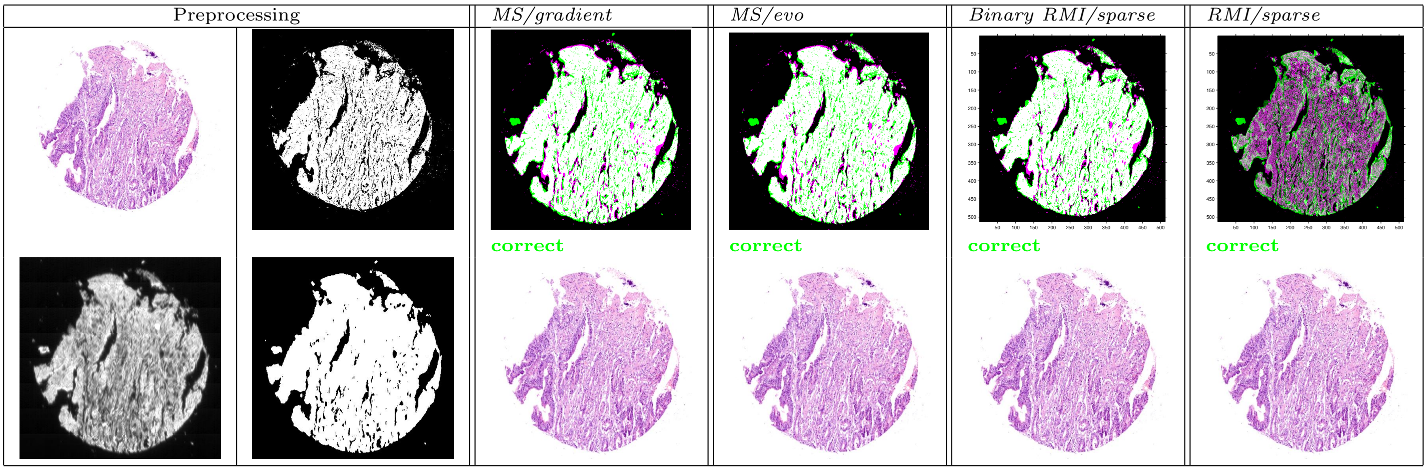

Spot C7

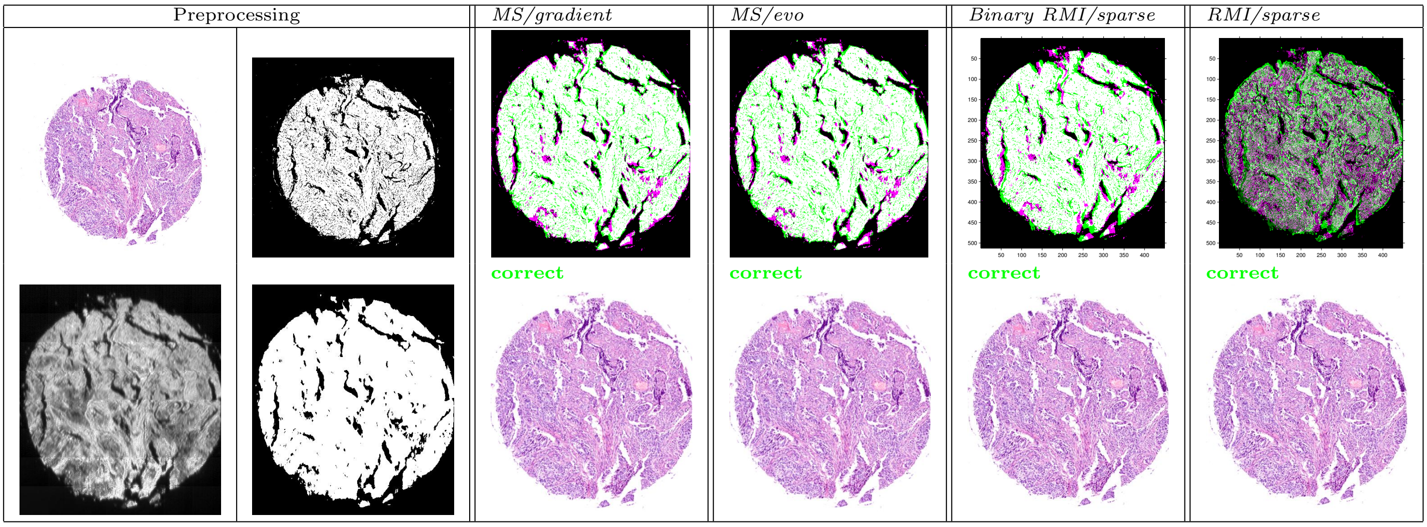

Spot C8

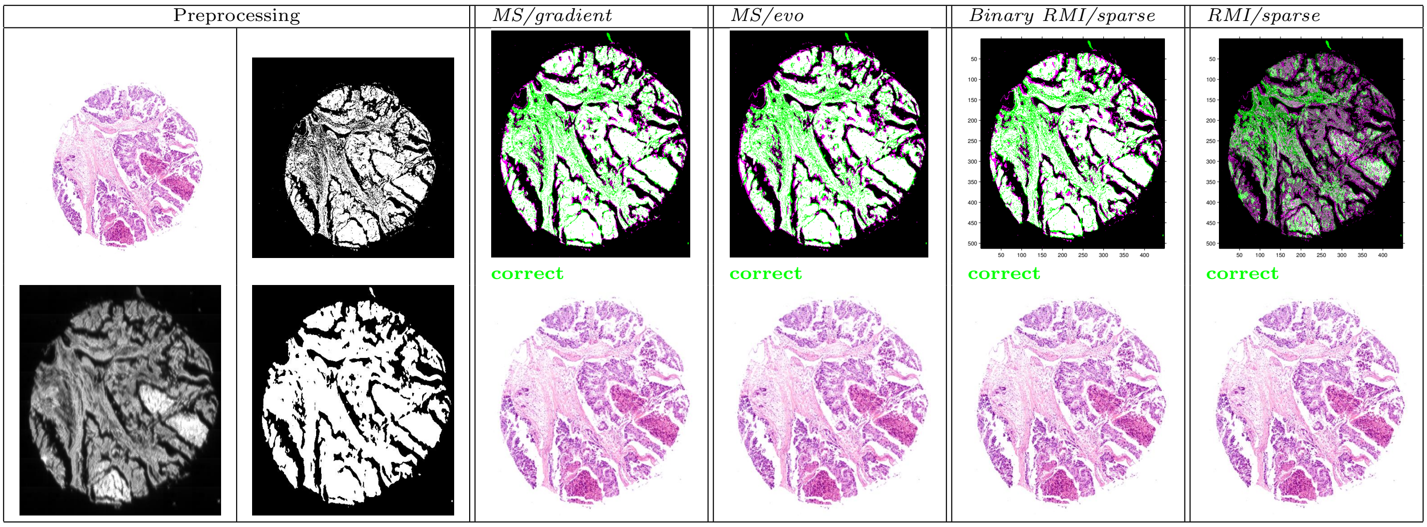

Spot C9

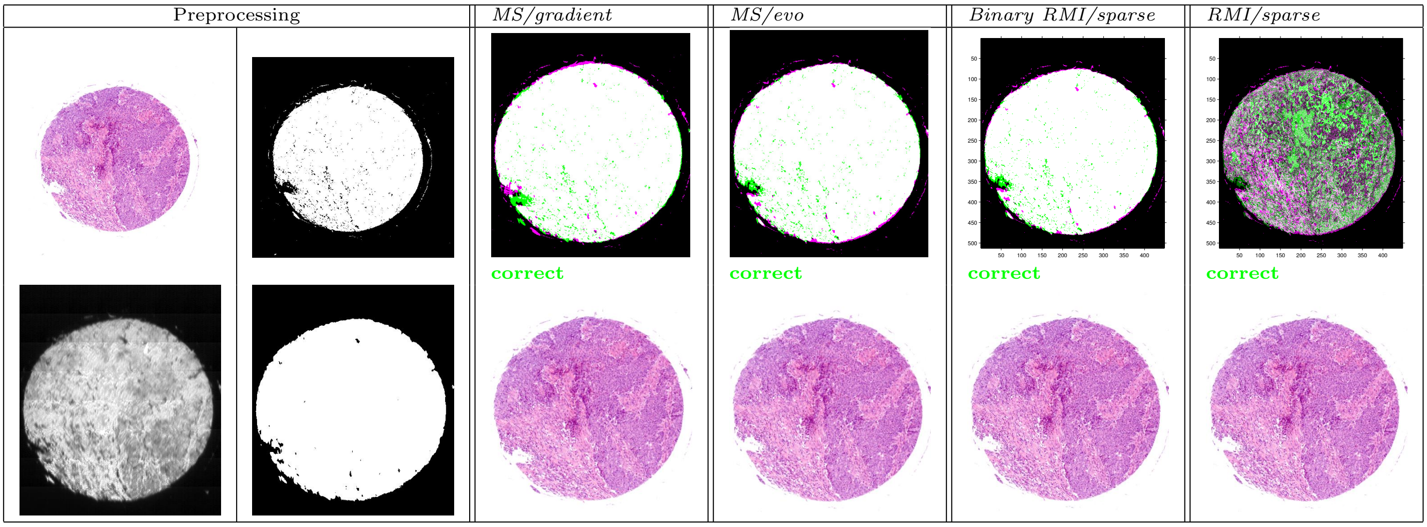

Spot C10

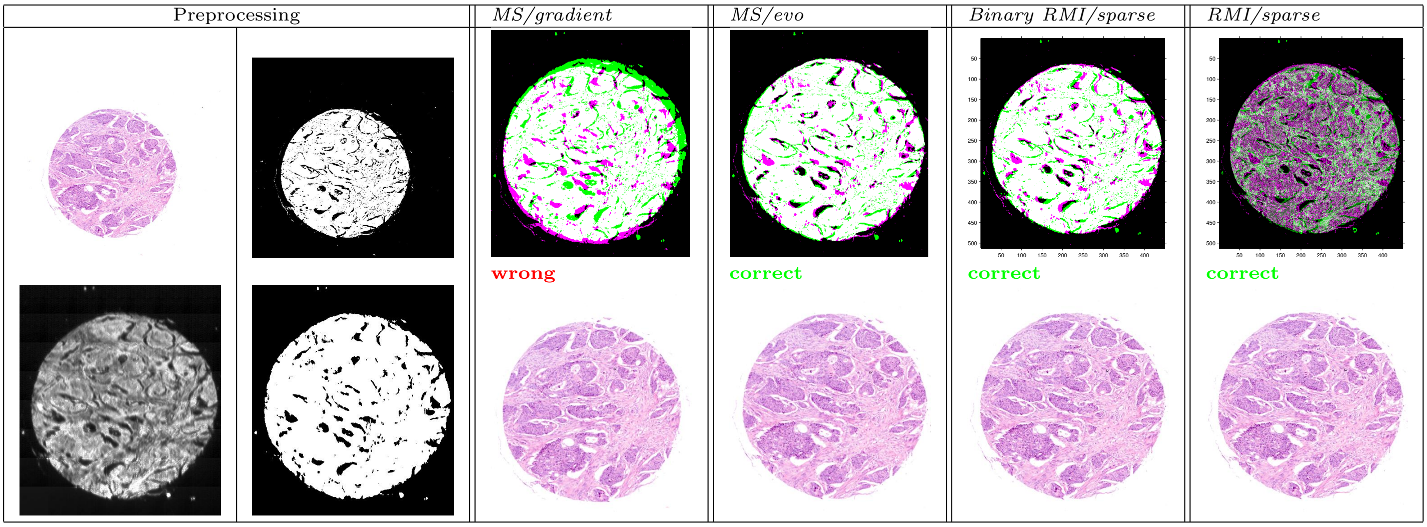

Spot E6

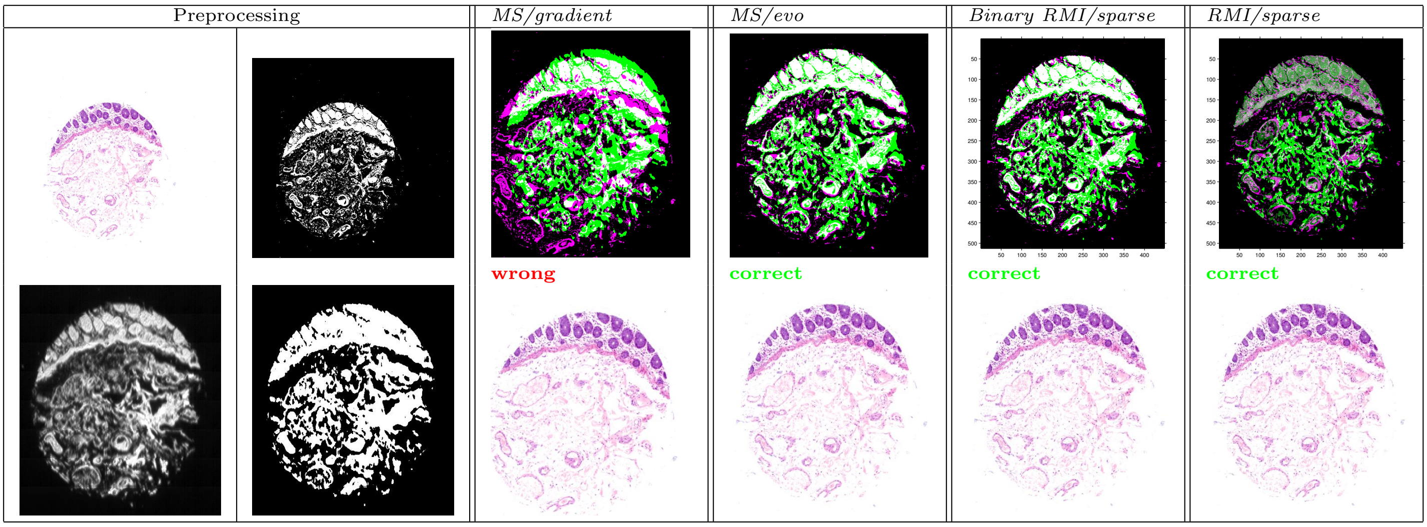

Spot F1

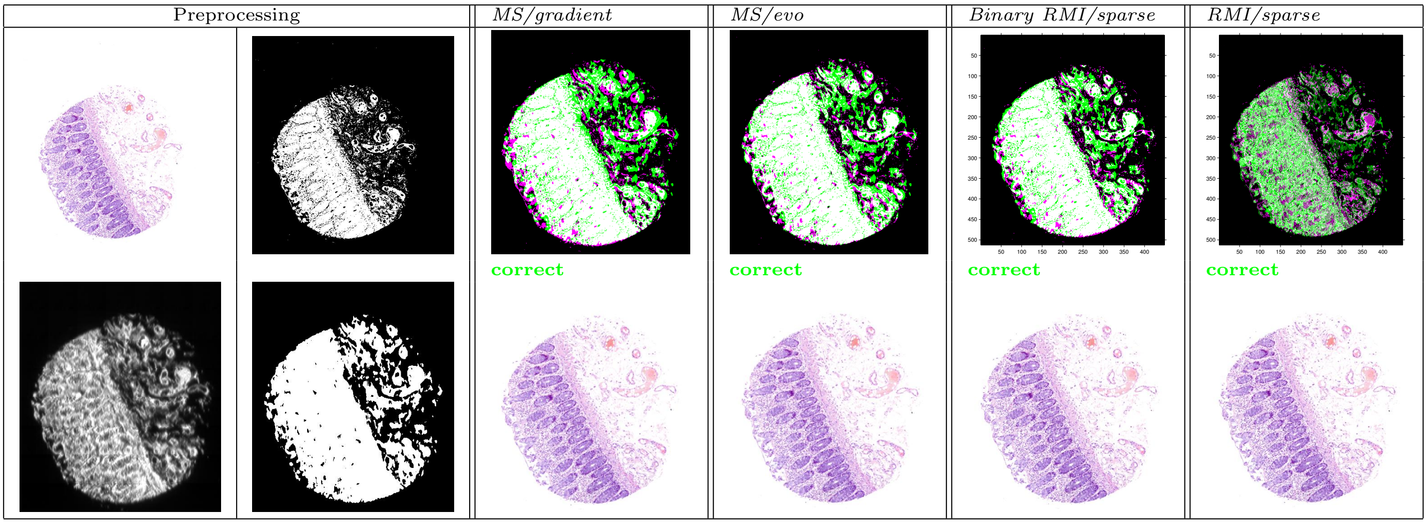

Spot F2

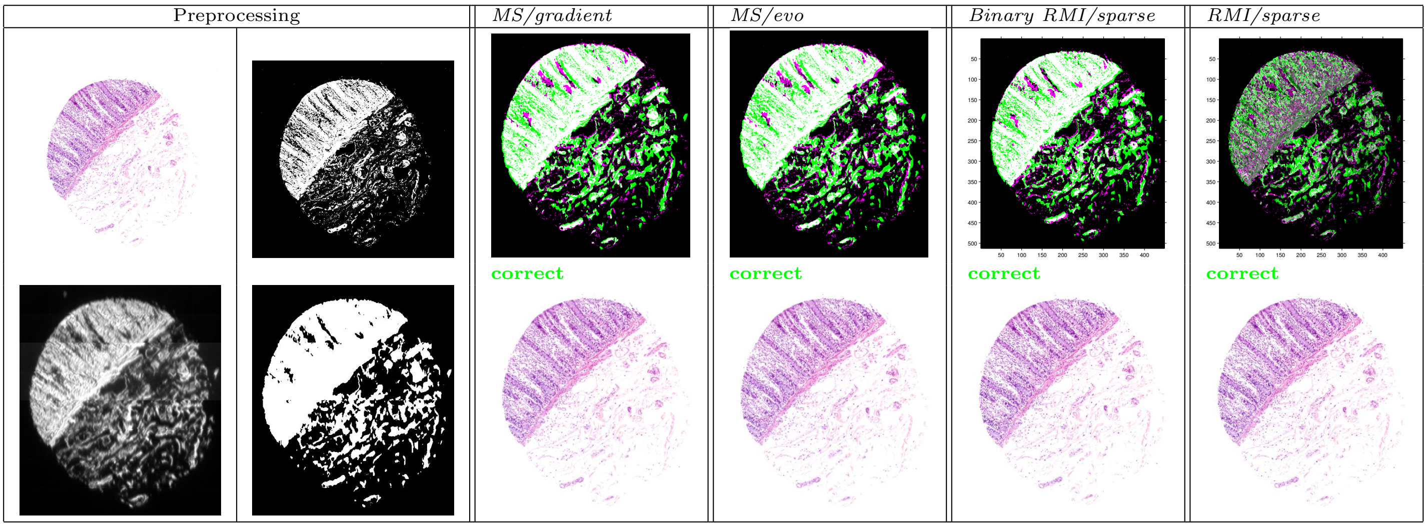

Spot F3

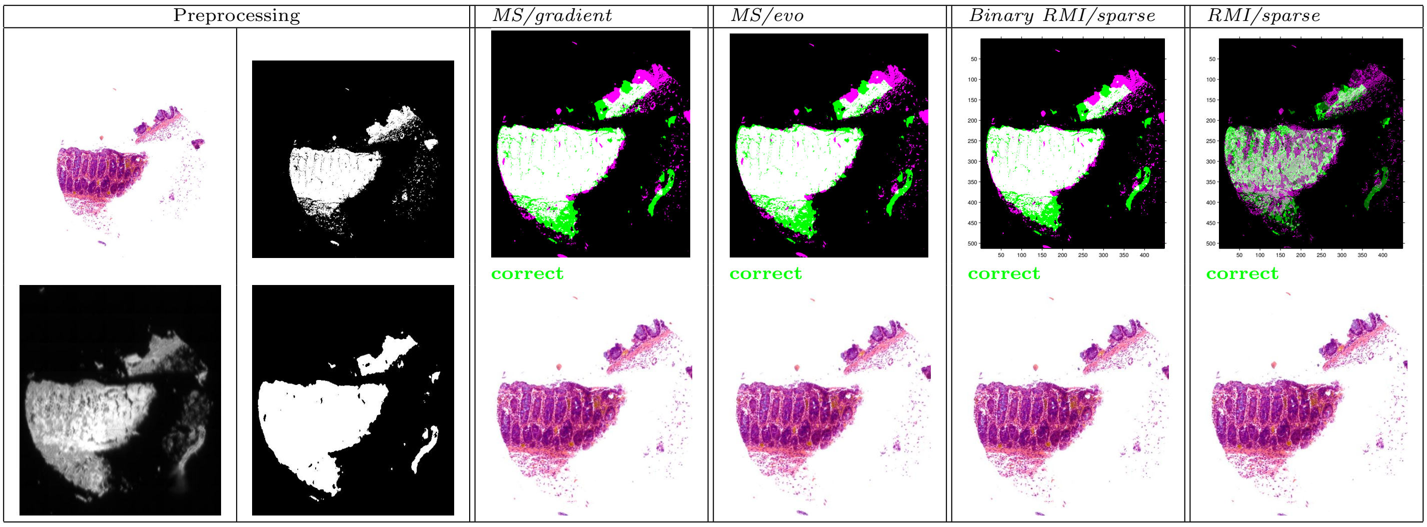

Spot F4

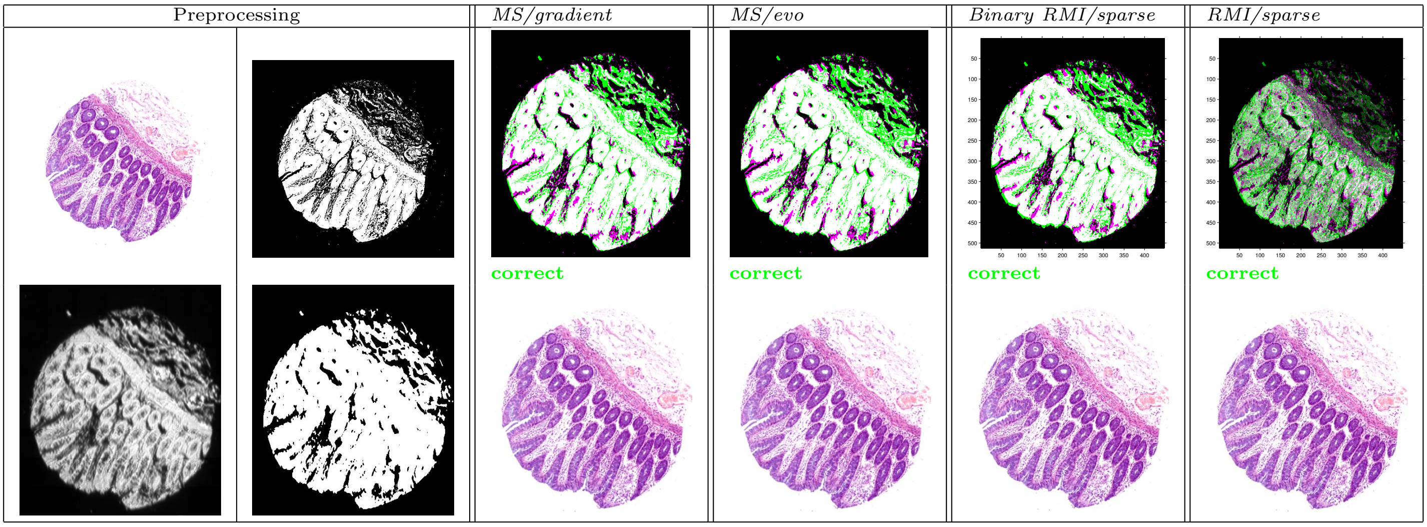

Spot F5

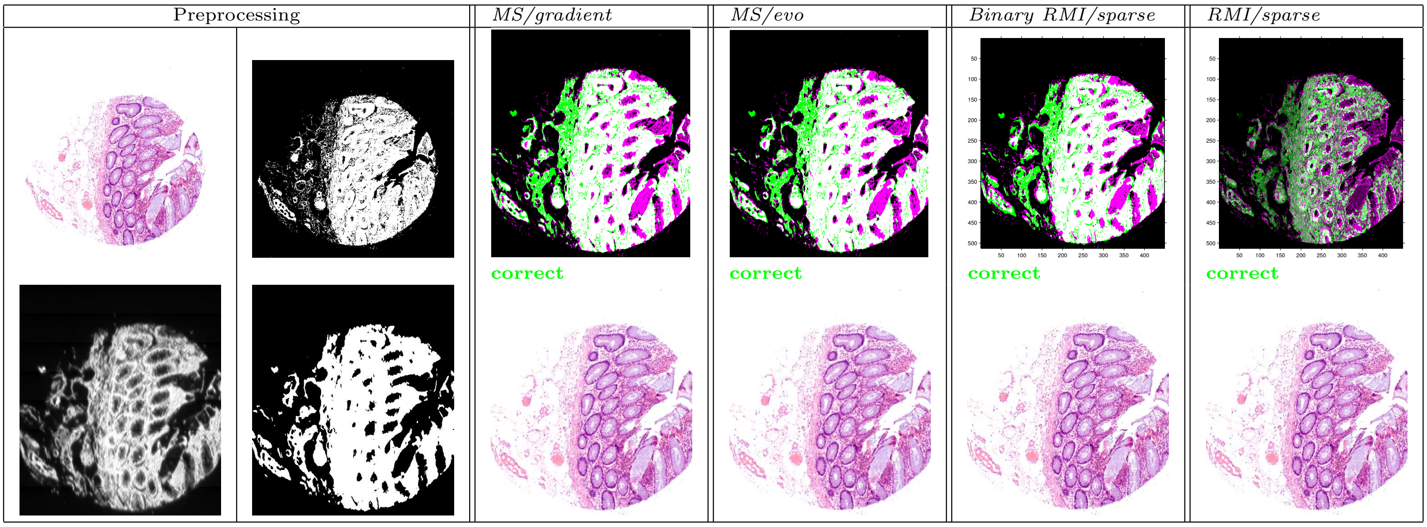

Spot F6

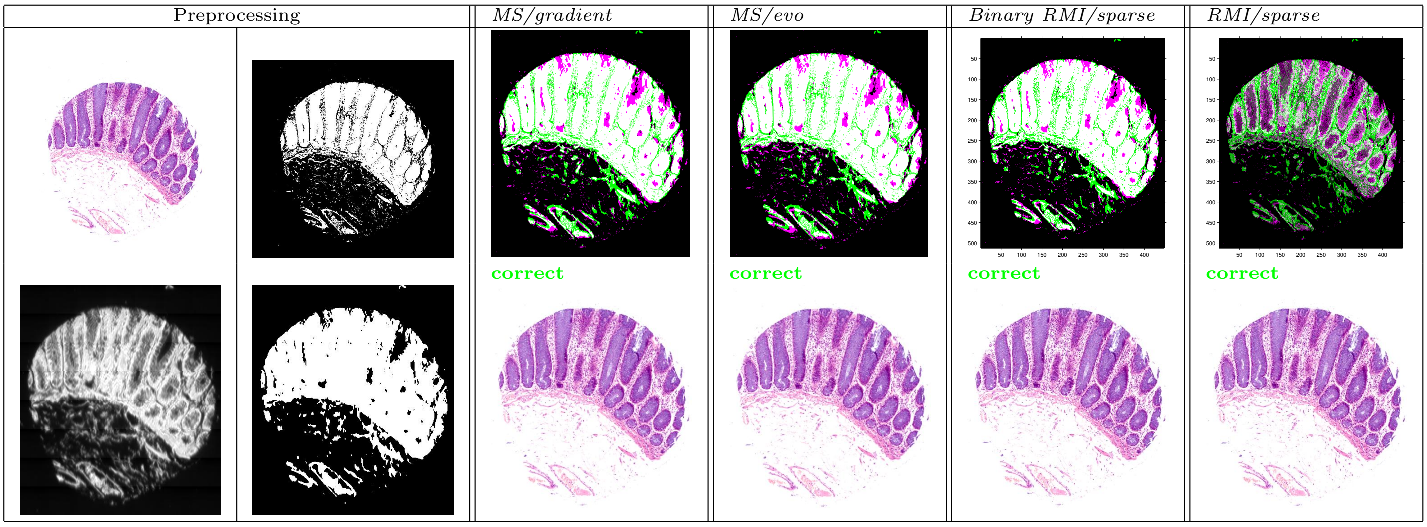

Spot F7

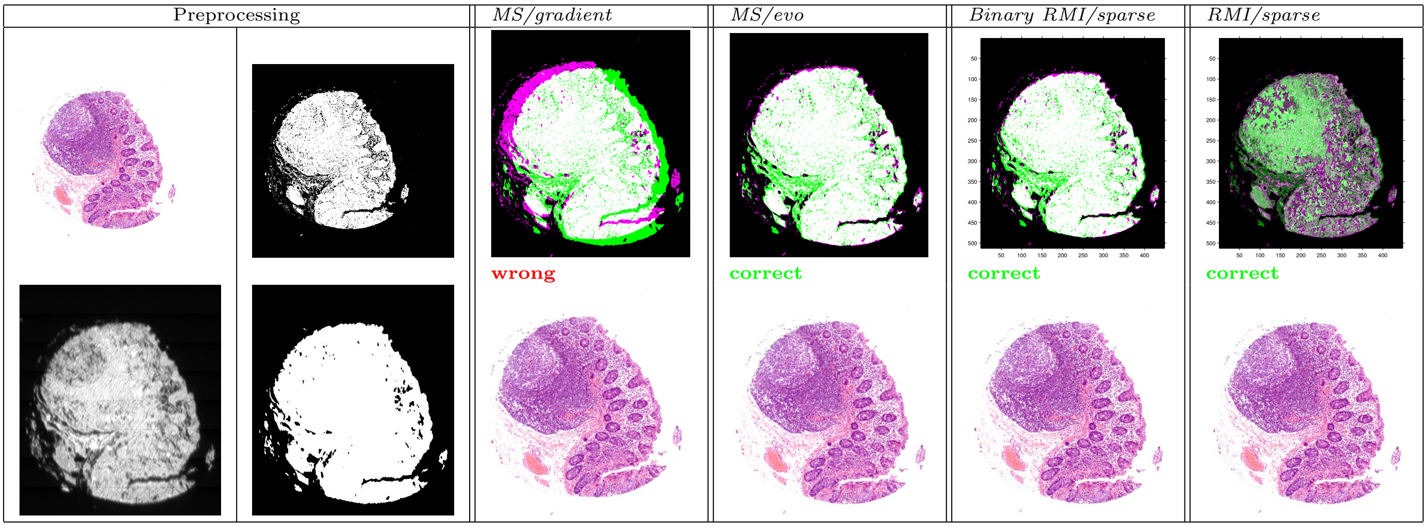

Spot G3

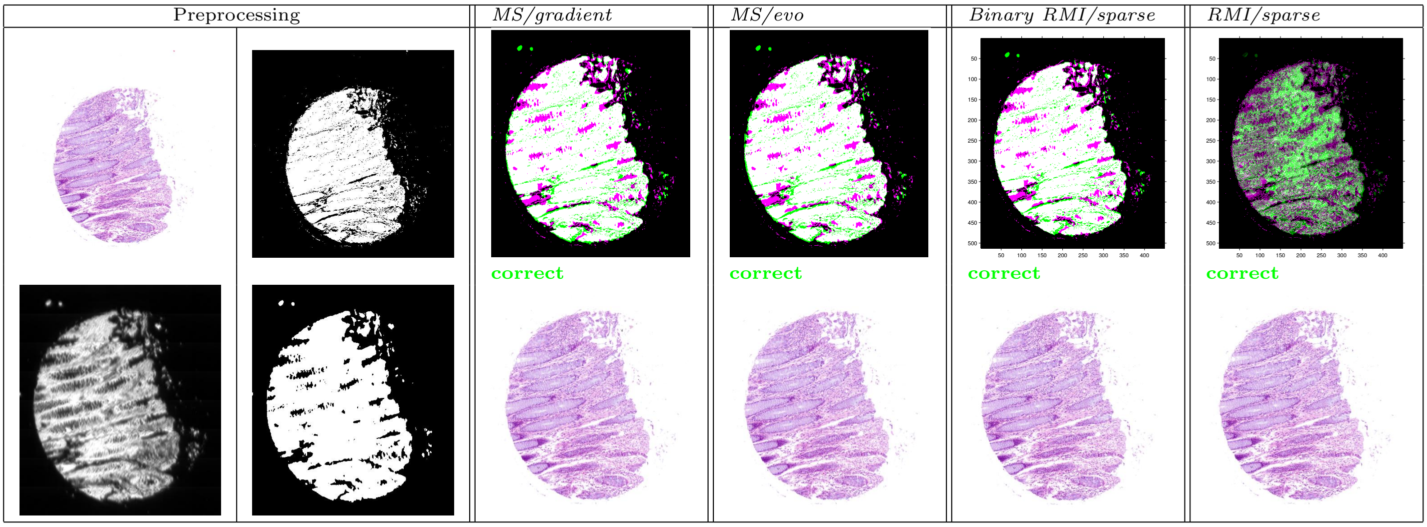

Spot G4

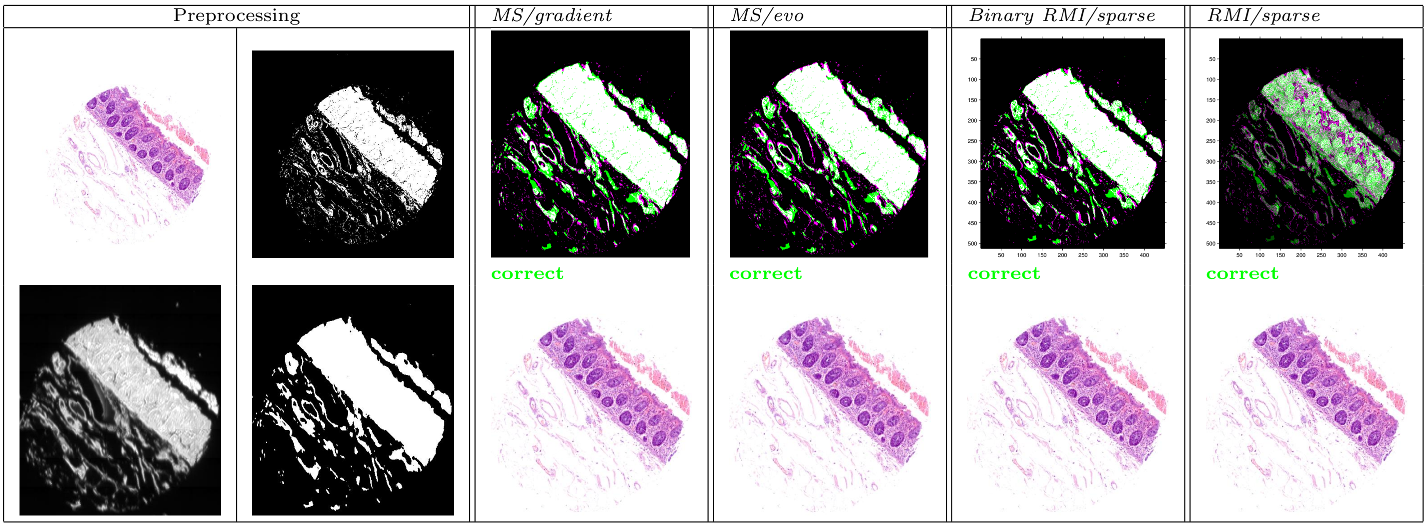

Spot G5

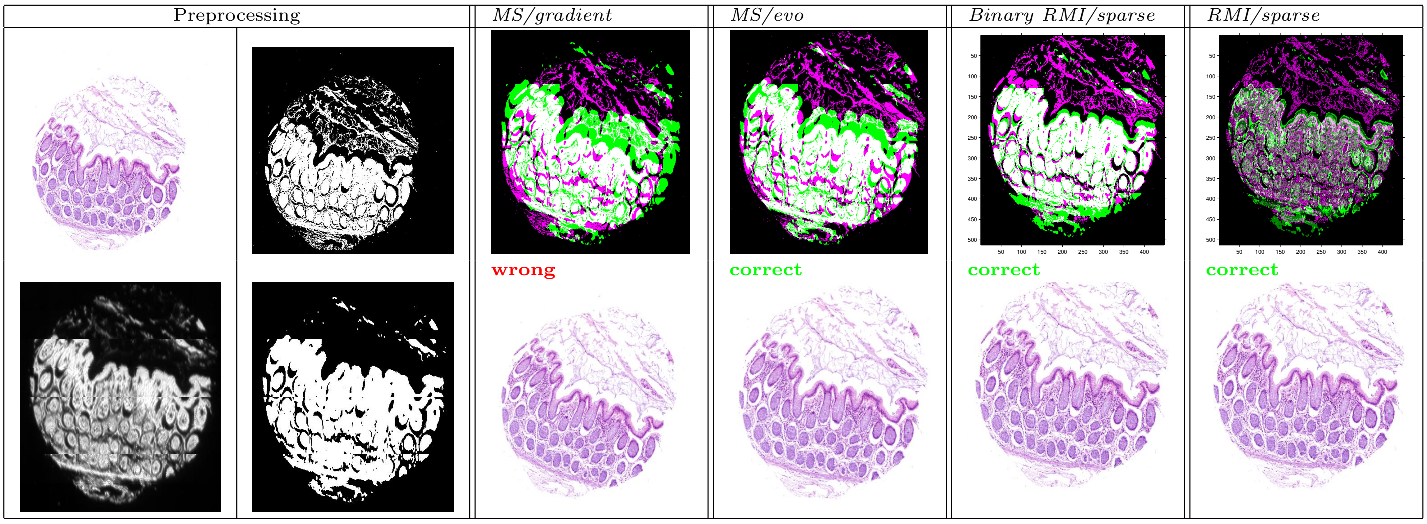

Spot G6

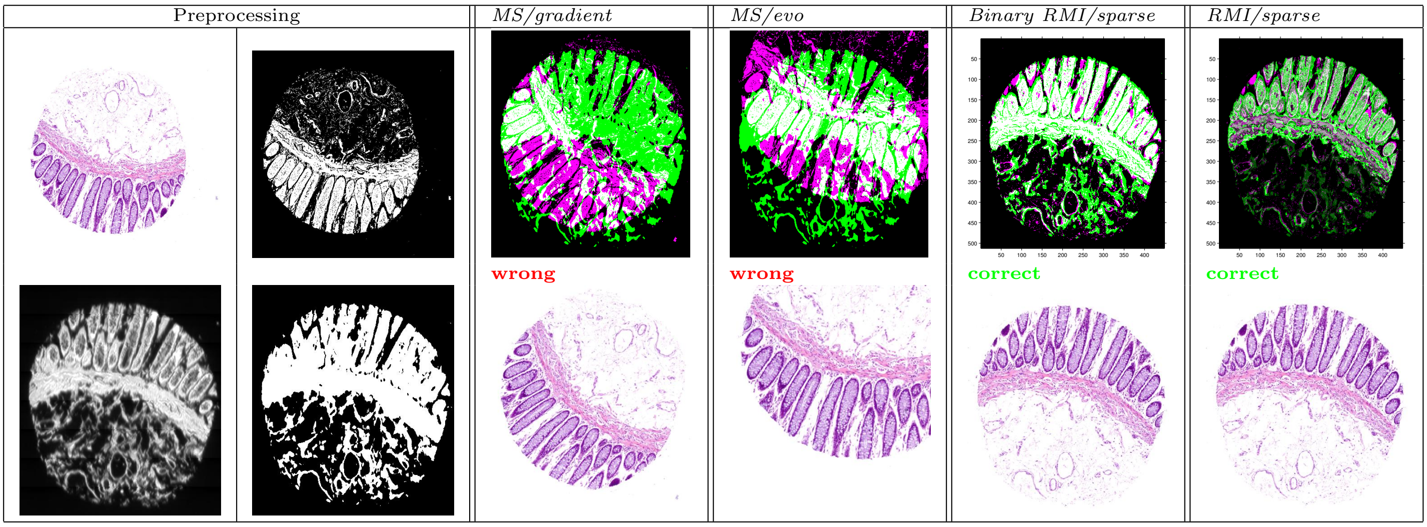

Spot G7

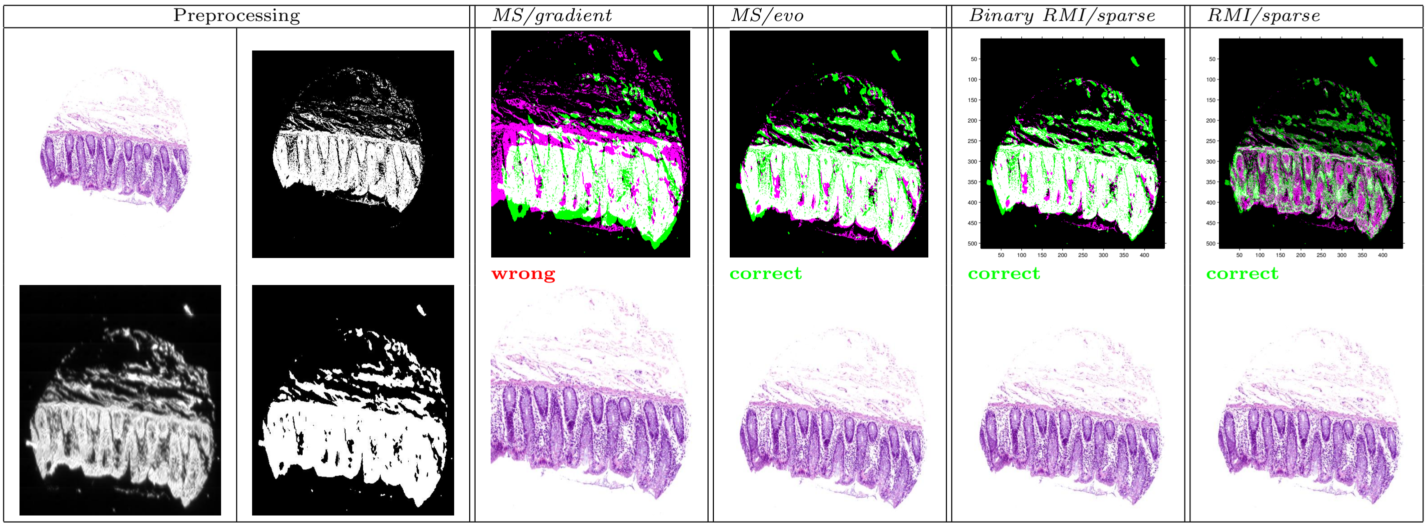

Spot G8

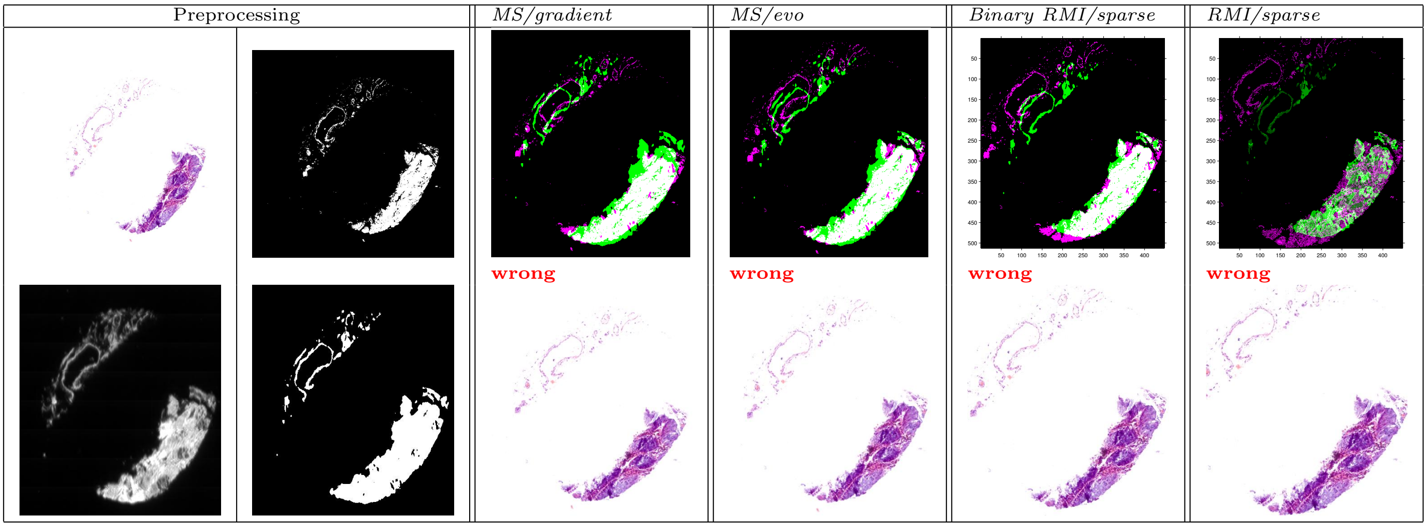

Spot G9

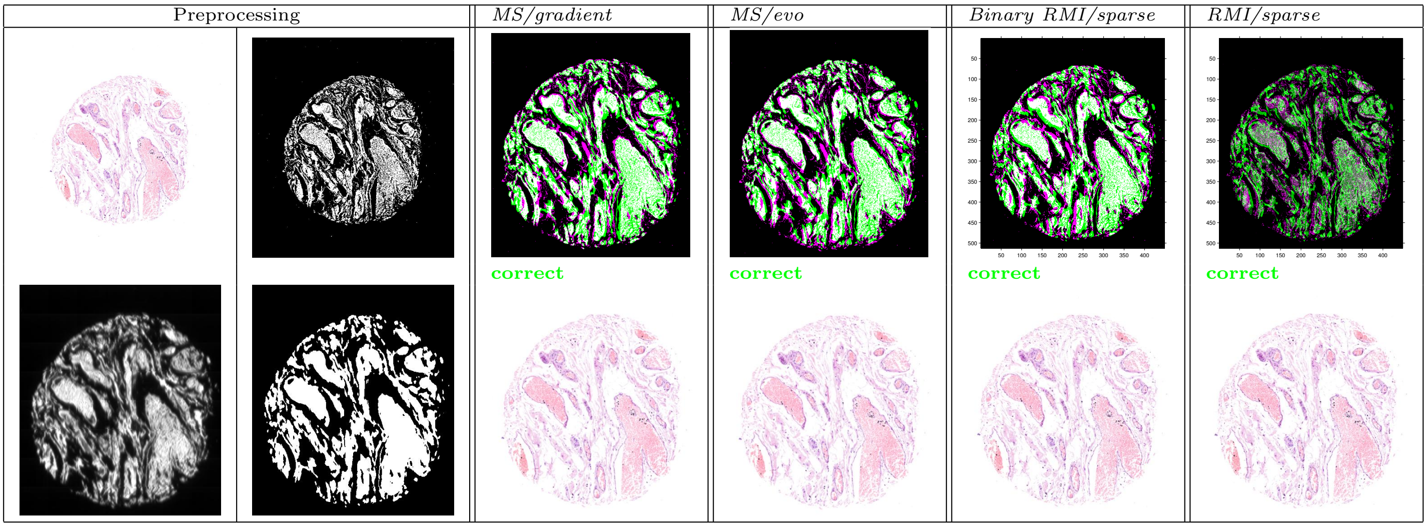

Spot I2

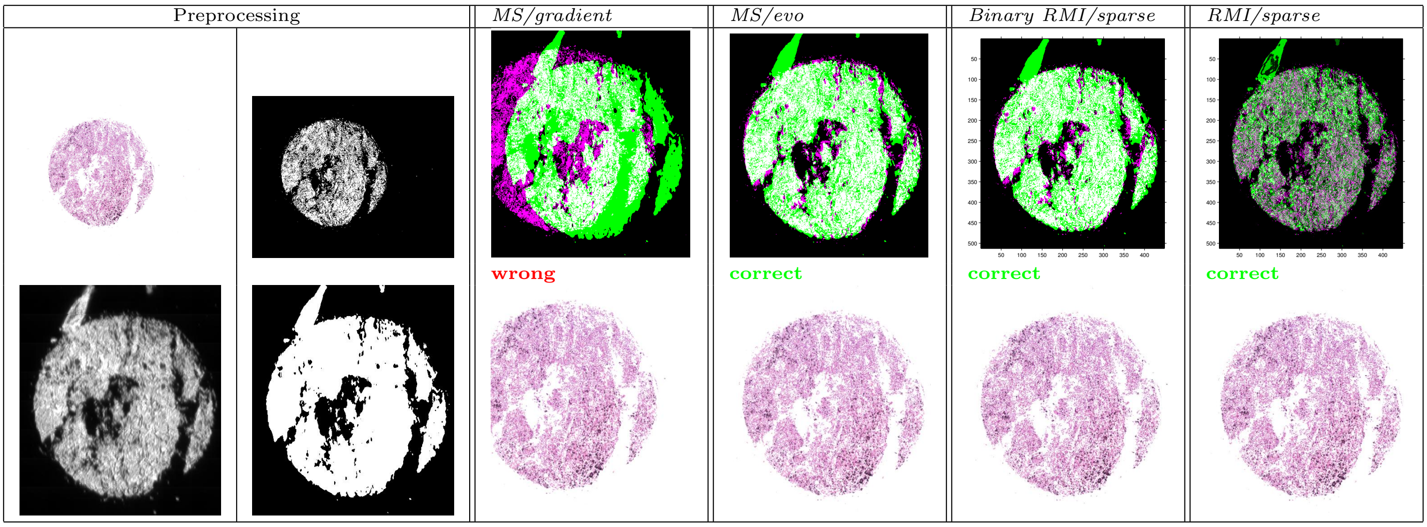

Spot I3

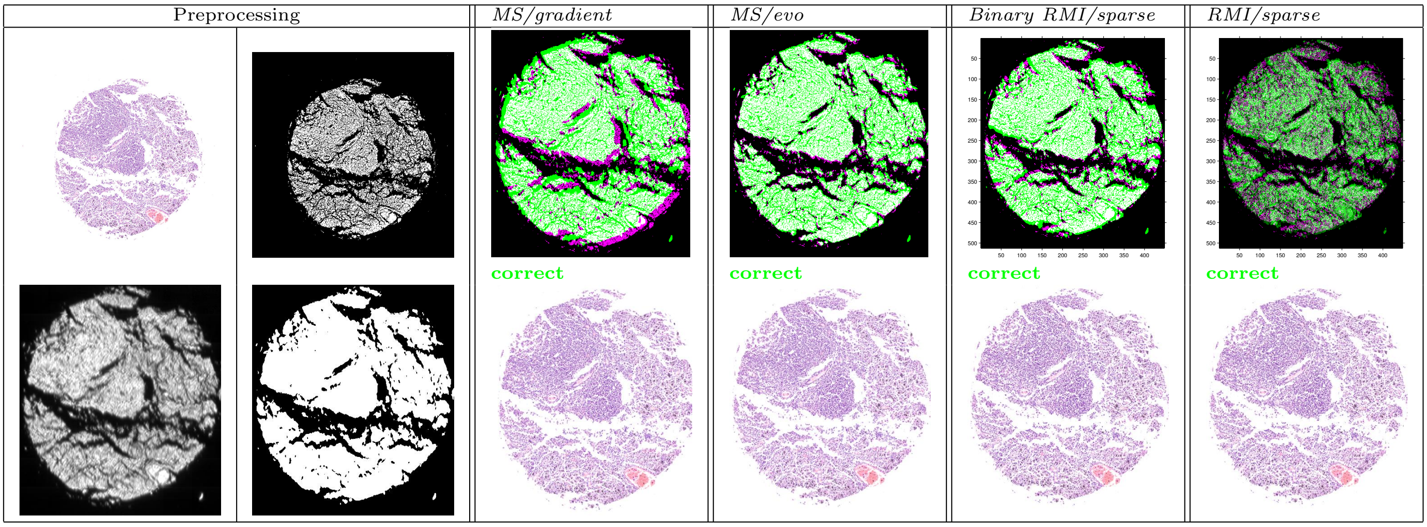

Spot I4

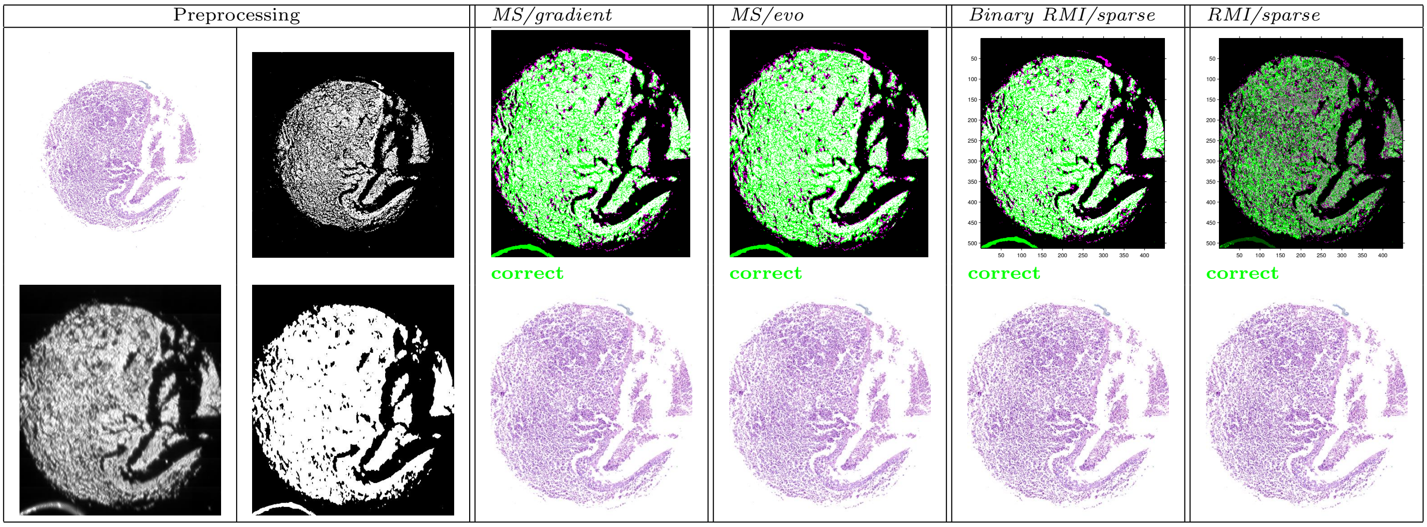

Spot I5

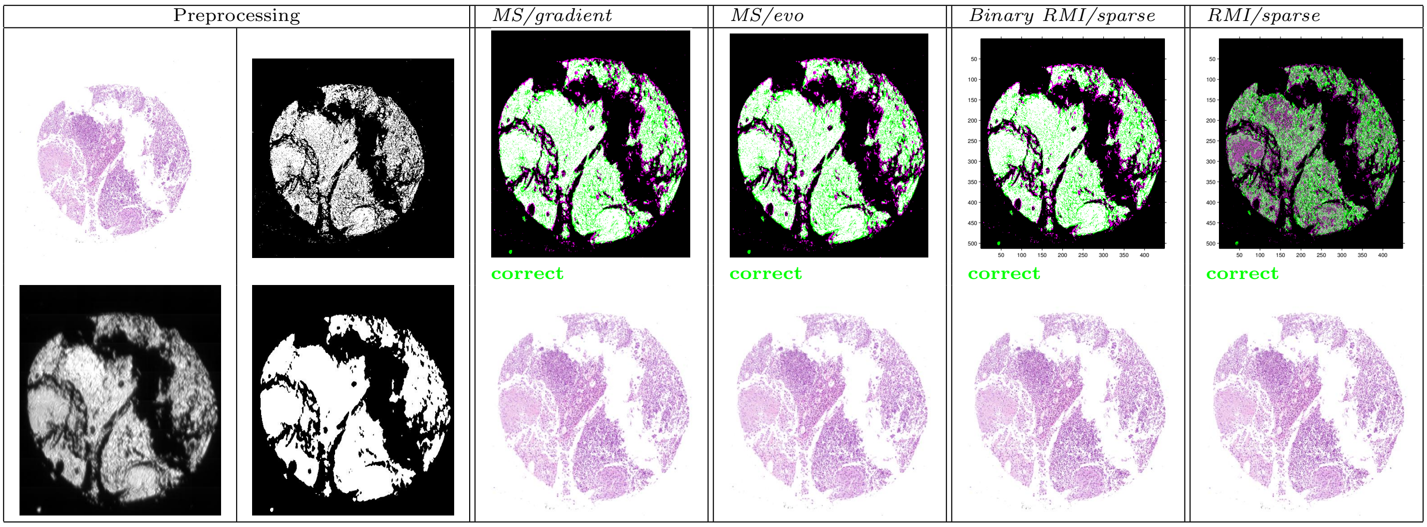

Spot I6

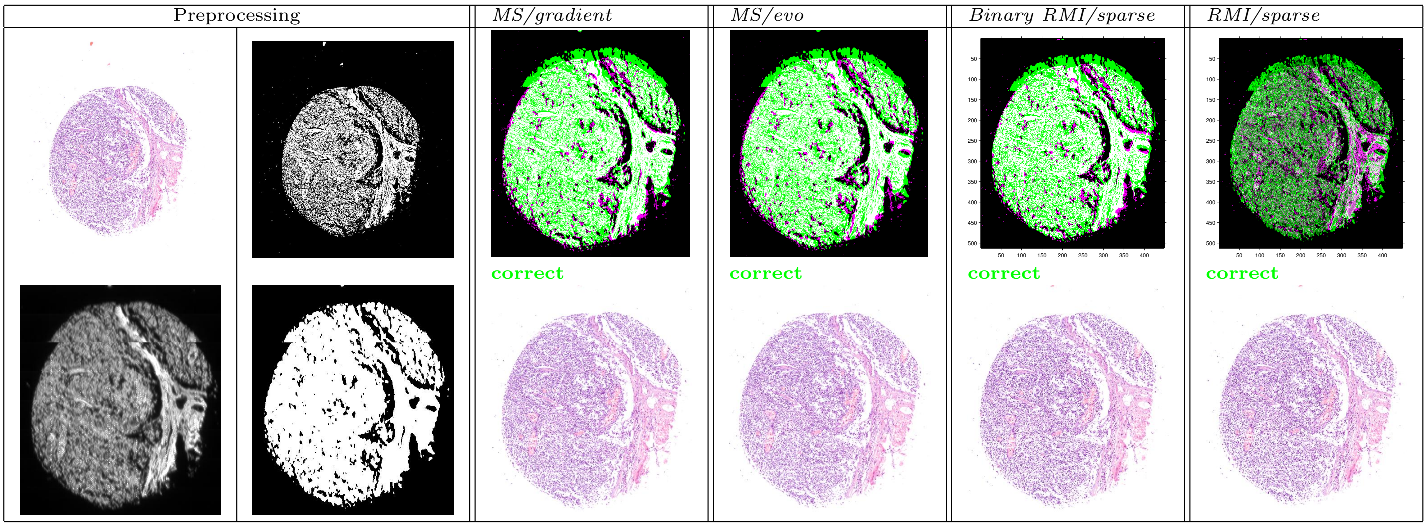

Spot I7

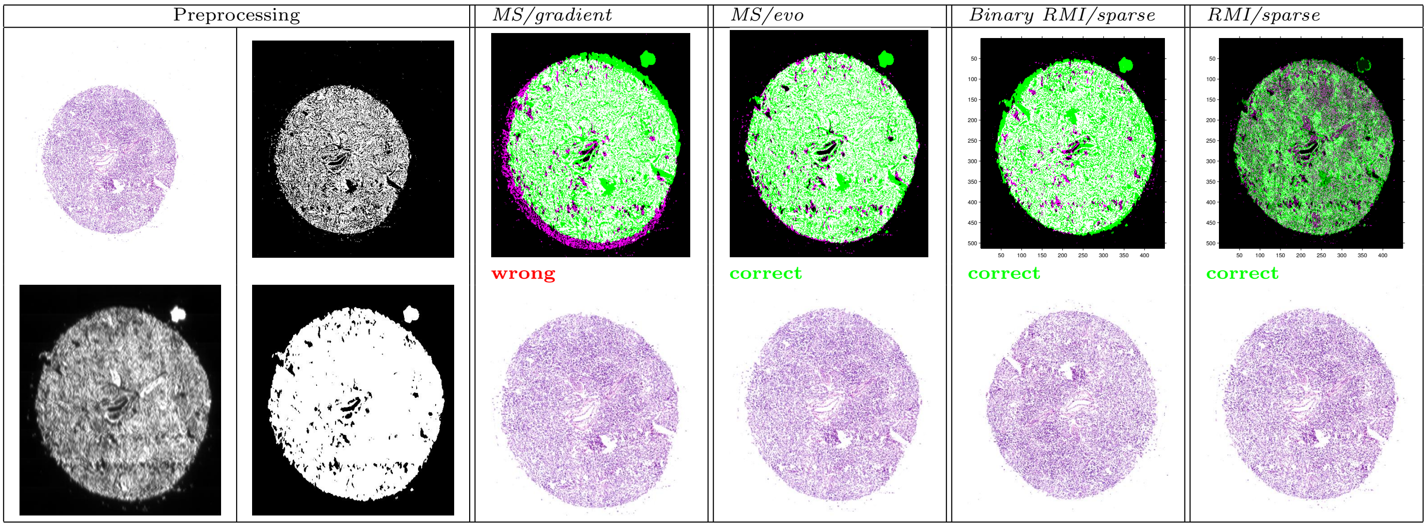

Spot I8

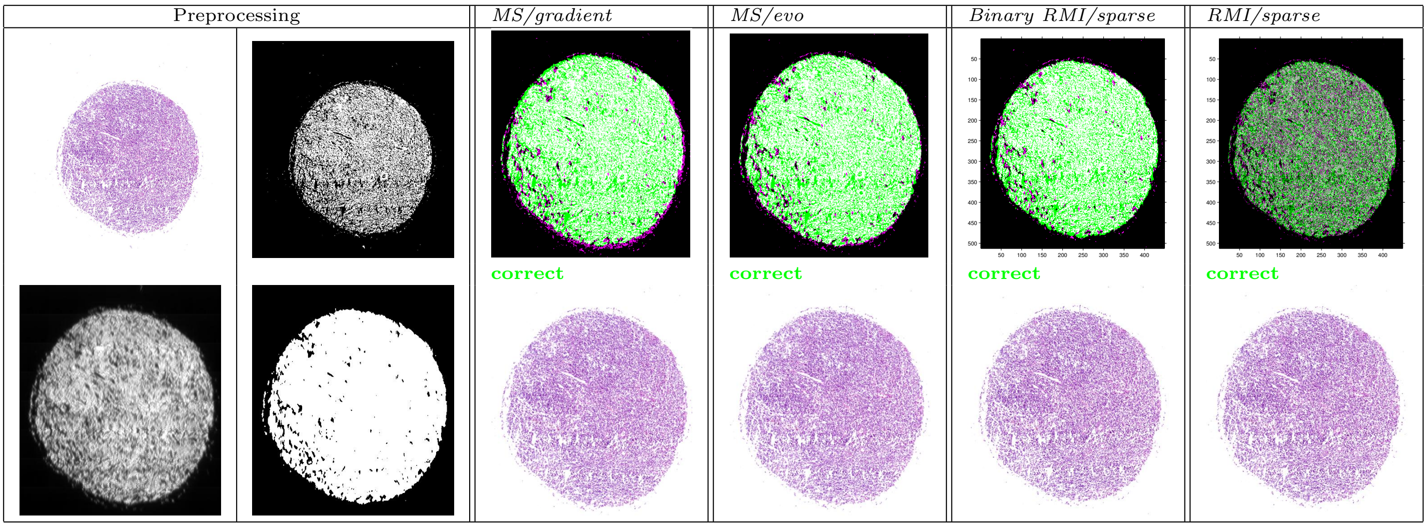

Spot I9

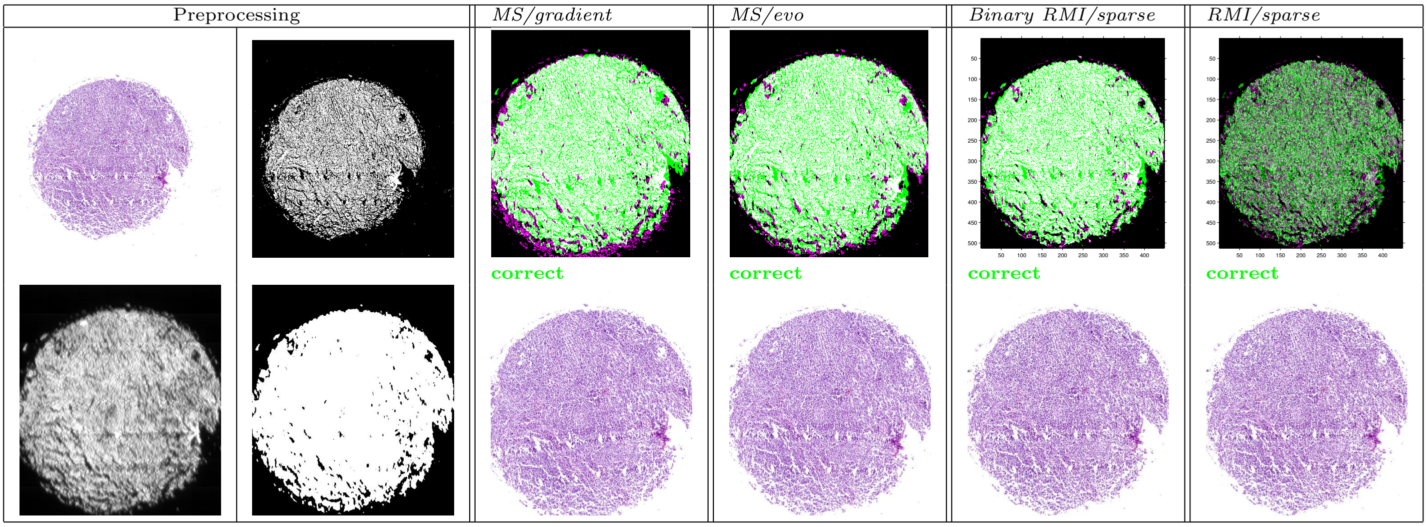

Spot J3

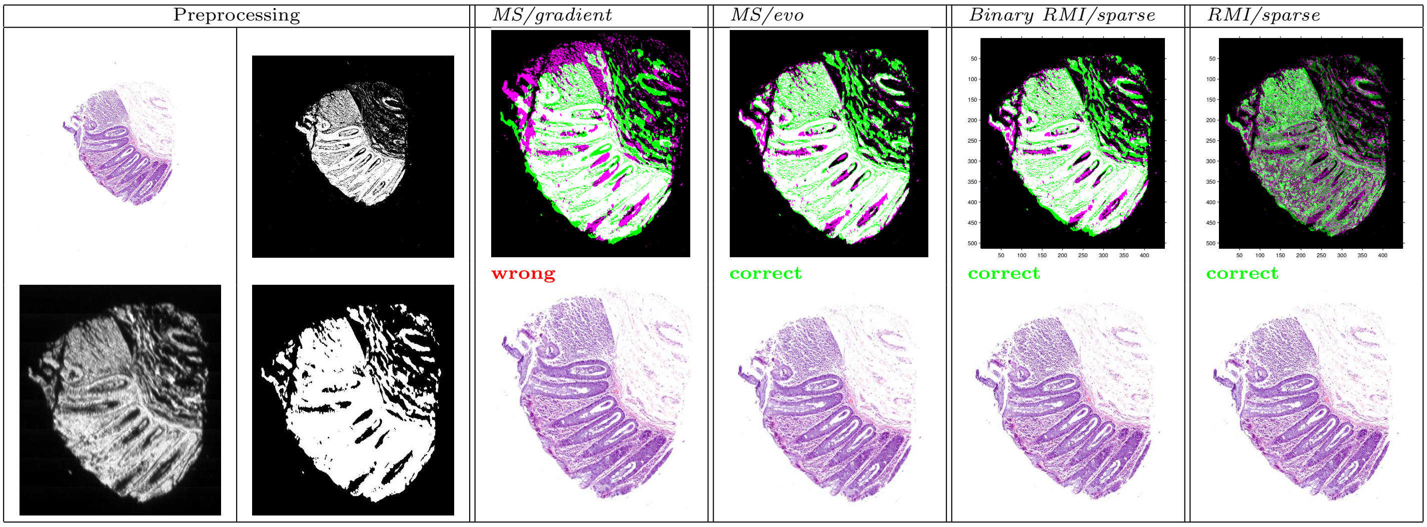

Spot J4

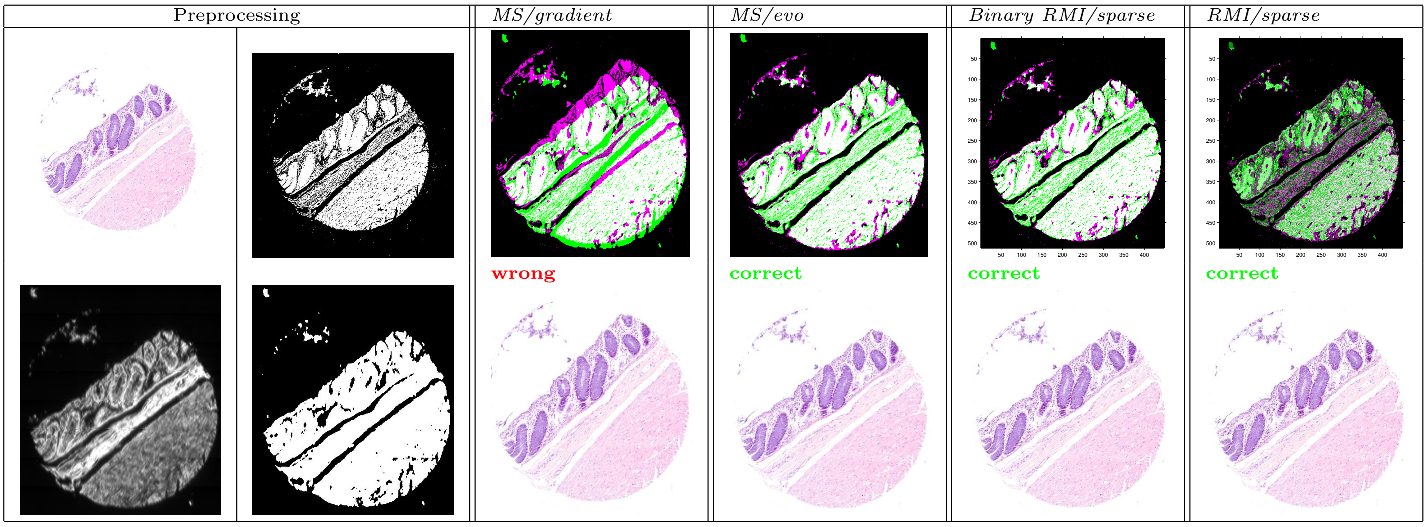

Spot J5

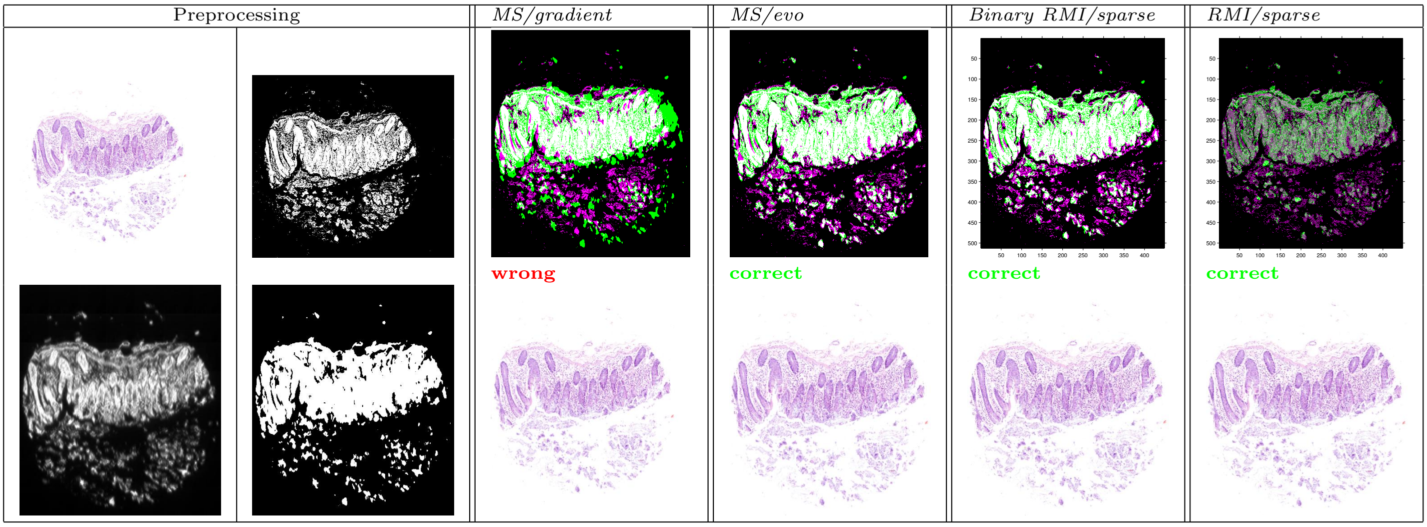

Spot J7

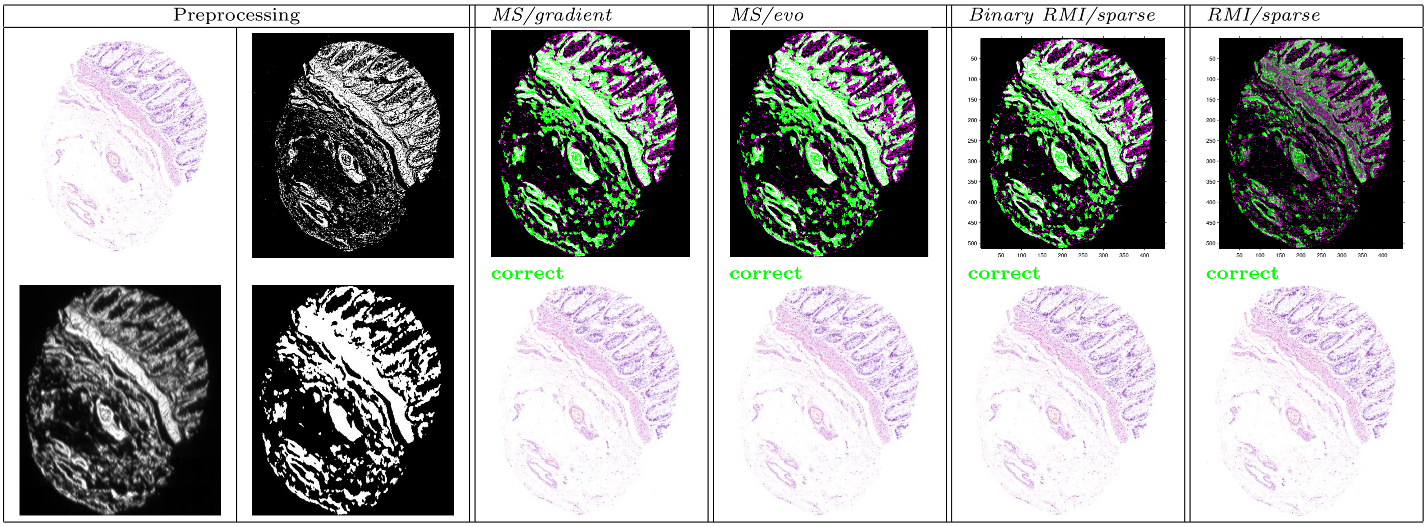

Spot J8

Preprocessing		*MS/gradient*	*MS/evo*	*Binary RMI/sparse*	*RMI/sparse*
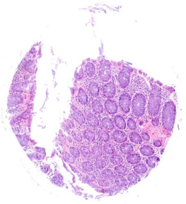	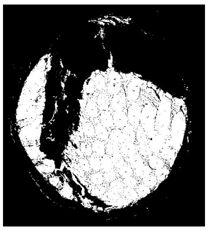	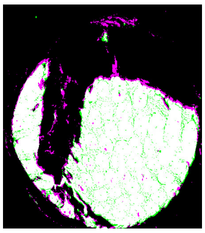 correct	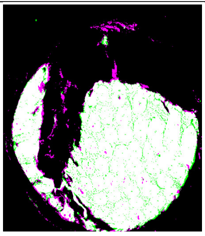 correct	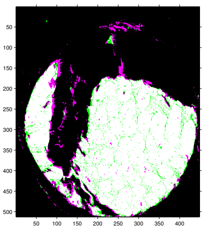 correct	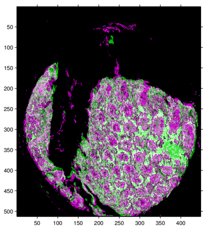 correct
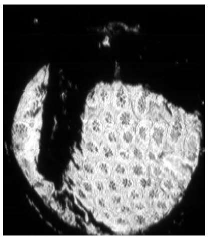	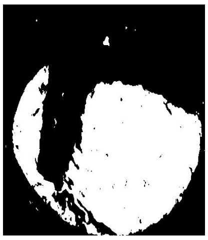	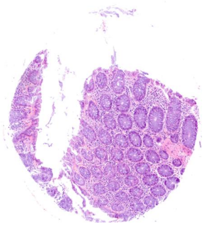	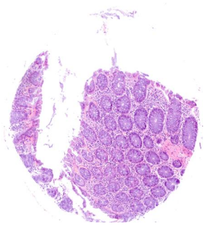	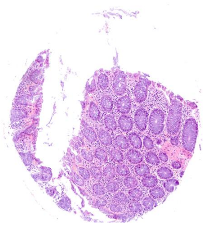	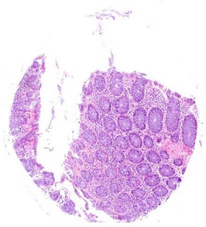
